# Endowing universal CAR T-cell with immune-evasive properties using TALEN-gene editing

**DOI:** 10.1038/s41467-022-30896-2

**Published:** 2022-06-30

**Authors:** Sumin Jo, Shipra Das, Alan Williams, Anne-Sophie Chretien, Thomas Pagliardini, Aude Le Roy, Jorge Postigo Fernandez, Diane Le Clerre, Billal Jahangiri, Isabelle Chion-Sotinel, Sandra Rozlan, Emilie Dessez, Agnes Gouble, Mathilde Dusséaux, Roman Galetto, Aymeric Duclert, Emanuela Marcenaro, Raynier Devillier, Daniel Olive, Philippe Duchateau, Laurent Poirot, Julien Valton

**Affiliations:** 1 Cellectis, Inc., 430 East 29th Street, New York, NY 10016 USA; 2 Institut Paoli-Calmettes; Aix-Marseille Université UM105, CNRS UMR 7258, 13009 Marseille, France; 3 Cellectis, 8 rue de la Croix Jarry, 75013 Paris, France; 4 Department of Experimental Medicine, University of Genova, Genova, Italy

**Keywords:** Tissue engineering, Immune evasion, Allotransplantation, Acute lymphocytic leukaemia

## Abstract

Universal CAR T-cell therapies are poised to revolutionize cancer treatment and to improve patient outcomes. However, realizing these advantages in an allogeneic setting requires universal CAR T-cells that can kill target tumor cells, avoid depletion by the host immune system, and proliferate without attacking host tissues. Here, we describe the development of a novel immune-evasive universal CAR T-cells scaffold using precise TALEN-mediated gene editing and DNA matrices vectorized by recombinant adeno-associated virus 6. We simultaneously disrupt and repurpose the endogenous TRAC and B2M loci to generate TCRαβ- and HLA-ABC-deficient T-cells expressing the CAR construct and the NK-inhibitor named HLA-E. This highly efficient gene editing process enables the engineered T-cells to evade NK cell and alloresponsive T-cell attacks and extend their persistence and antitumor activity in the presence of cytotoxic levels of NK cell in vivo and in vitro, respectively. This scaffold could enable the broad use of universal CAR T-cells in allogeneic settings and holds great promise for clinical applications.

## Abstract (web summary)

Host versus graft reaction is a major impediment to CAR-T cell immune therapy in allogeneic settings. Authors show here that CAR-T cells, engineered to be deficient in MHC I expression but to express the NK inhibitor HLA-E, are resistant to destruction by both T and NK cells of the host.

## Introduction

Universal chimeric antigen receptor (CAR) expressing T cells have great potential to democratize and improve the treatment of cancer patients worldwide. Reasons for such potential are multiple but all stem from the source of the biological material used to produce them. Because universal CAR T cells are engineered out of third-party healthy donor T cells, donors can be carefully chosen for potency, and cells can be manufactured, formulated, and controlled thoroughly before being adoptively transferred to multiple patients in an allogeneic setting.

To realize their full potential in an allogeneic setting, universal CAR T cells must not induce two detrimental and potentially toxic phenomena: the graft-versus-host (GvH) reaction and the Host versus Graft (HvG) reaction. The GvH reaction can be readily addressed by the transient or constitutive inactivation of T-cell receptor αβ (TCRαβ) expression in CAR T cell^1–4^. In contrast, preventing the depletion of CAR T cells due to the HvG reaction is less straightforward. The preconditioning regimen that is typically used to lymphodeplete patients prior CAR T-cell transfer (cyclophosphamide and fludarabine)^5,6^ can delay the HvG reaction and create a first window of opportunity for CAR T-cell engraftment. However, because this lymphodepletion is transient, it may not fully prevent the HvG reaction. In addition to human leukocyte antigen (HLA) matching between CAR T-cell donors and recipients^7,8^, two main engineering strategies have been thoroughly assessed for their ability to further inhibit or delay the HvG reaction. The first strategy relies on developing drug-resistant CAR T cells wherein TCRαβ and genes that modulate sensitivity to lymphodepleting drugs (CD52 and dCK which are responsible for alemtuzumab binding and fludarabine metabolism^2,9^, respectively) are inactivated. This strategy, which is designed to allow for CAR T-cell engraftment and proliferation under prolonged lymphodepletion of the allogeneic host, showed encouraging antitumor potency in clinical trials in the presence of alemtuzumab^10,11^. The second strategy relies on the genetic inactivation of beta-2 microglobulin (B2M) in CAR T cells. The inactivation of B2M prevents the expression of the HLA Class-I surface marker that is responsible, in part, for the host T-cell-mediated HvG reaction^12–14^. TCRαβ(−) HLA-ABC(−) CAR T cells can be efficiently generated via several gene-editing methods, are hypoimmunogenic with respect to alloresponsive T cells^12–15^, and are currently being evaluated in a phase I clinical trial.

This second approach, often presented as the next generation of universal CAR T-cell treatment, offers the potential advantage of extending the engraftment of CAR T cells without relying on prolonged lymphodepletion which heightens the risk of opportunistic infections and reduces any potential benefits provided by endogenous immune effectors. However, while highly attractive, this strategy is likely to be impaired by the presence of host NK cells, which recognize and readily deplete HLA-ABC(−) T cell through the missing self-response^12^. Clinical studies investigating the antitumor potential of HLA-ABC(−) CAR T cells will be informative but it is difficult to predict whether NK cells will recover in number and fitness to mediate a missing self-response following lymphodepletion. Therefore, realizing the full potential of universal HLA-ABC(−) CAR T cells will require new engineering strategies that can enable them to evade host NK-cell attacks.

Here, we report the development of an immune-evasive universal CAR T-cell scaffold named ΔTRAC_CAR_ΔB2M_HLAE_, which incorporates disruptive insertions of a CAR and HLA-E, a non-polymorphic NK inhibitor^16^ into the TRAC and B2M loci, respectively. Using a combination of multiplex TAL Effector Nucleases (TALEN) and recombinant adeno-associated virus 6 (AAV6) treatments, we show that ΔTRAC_CAR_ΔB2M_HLAE_ can be efficiently produced, displays antitumor activity, and resists primary alloresponsive T cells and primary NK cells sourced from healthy donors and from acute myeloid leukemia (AML) patients. These findings demonstrate the immune-evasive properties of ΔTRAC_CAR_ΔB2M_HLAE_ and support its utilization as an off-the-shelf universal CAR T-cell product that is compatible with adoptive cell transfer in allogeneic settings. Further process development of ΔTRAC_CAR_ΔB2M_HLAE_ production needs to be performed with relevant CAR constructs, GMP media, and materials before moving forward to clinical evaluation.

## Results

### Efficient production of ΔTRAC_CAR_ΔB2M_HLAE_ by disruptive insertions of CAR and HLA-E at the TRAC and B2M loci, respectively

**Fig. 1:**
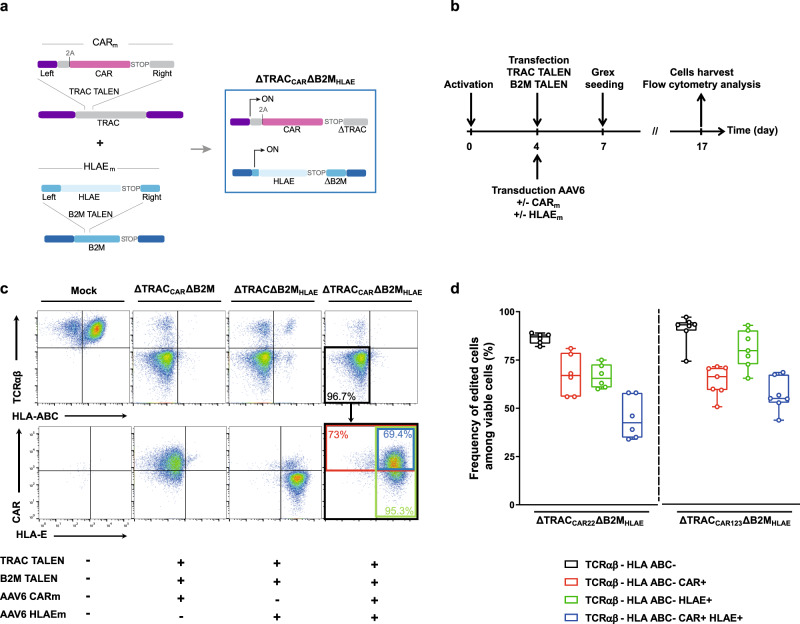
TALEN-mediated multiplex editing enables efficient CAR and HLA-E expression in TCRαβ/B2M double knockout T cells. **a** Schematic showing the editing strategy at the TRAC and B2M loci to generate ΔTRAC_CAR_ΔB2M_HLAE_ T cells from wild-type T cells. **b** Experimental design for multiplex editing of TRAC and B2M loci using TALEN and adeno-associated viral (AAV6) particles and analysis of the resulting engineered T cells. **c** Representative flow-cytometry analysis of mock-transfected T cells and T cells engineered with different combinations of TALEN and AAV6 particles (CAR_123m_ and HLA-E_m_) **c**, top panel, flow-cytometry plots showing the frequency of TCRαβ and HLA-ABC expression in viable engineered T cells. **c** bottom panel, flow-cytometry plots showing the frequency of CAR and HLA-E expression detected among TCRαβ(−)/HLA-ABC(−) viable engineered T cells. The black bold box depicts the parent population. **d** Box plot showing the frequency of different subpopulations detected in engineered ΔTRAC_CAR22_ΔB2M_HLAE_ T cells (left) and ΔTRAC_CAR123_ΔB2M_HLAE_ T cells (right). The gating strategy used to obtain the frequency of each subpopulation is illustrated by the black, red, green, and blue bold boxes in (**c**), right flow-cytometry panel). In each box plot, the central mark indicates the median, the bottom and top edges of the box indicate the interquartile range (IQR), and the whiskers represent the maximum and minimum data point. Each point represents one experiment performed with a given donor (*n*  =  6 for ΔTRAC_CAR22_ΔB2M_HLAE_ and *n* = 7 for ΔTRAC_CAR123_ΔB2M_HLAE_). Source data are provided as a Source Data file.

Multiple approaches could be used to inhibit the cytolytic function of NK cells toward HLA-ABC(−) T cells^17–19^. To develop a robust and straightforward approach that is compatible with clinical applications, we chose to use the B2M gene to re-express the engineered NK inhibitor HLA-E, via a disruptive gene insertion approach (i.e., a knockout-by-knock-in). Based on the approach of ref. ^16^, we derived an AAV6 promoter-less DNA repair matrix that is specific for the B2M locus; we named this matrix HLA-E_m_. The HLA-E_m_ matrix includes a nonameric peptide derived from HLA-G (VMAPRTLIL^16,20,21^), codon-optimized B2M, and HLA-E domains covalently linked together by GS linkers (HLA-E^16,22^, Fig. 1a), and 300-bp left and right homology arms that are specific for exon 1 of the B2M locus. HLA-E_m_ was designed to be used in combination with a B2M-specific TALEN that would inactivate the endogenous B2M gene and repurpose its regulatory elements and reading frame to express the engineered HLA-E. In the following, the B2M-specific TALEN and HLA-E_m_ will be used in combination with the TRAC TALEN and the AAV6 promoter-less CAR matrix (CAR_m_, Fig. 1a), described earlier to mediate the disruptive insertion of a CAR construct at the TRAC locus^23^.

To assess the efficiency of TALEN-mediated CAR_m_ and HLA-E_m_ insertion at their respective loci, we simultaneously transfected T cells with mRNA encoding the TRAC and B2M TALEN and transduced them with CAR_m_ and HLA-E_m_ using the protocol described in Fig. 1b^23^. For this proof of concept, two different CAR_m_-encoding CAR tool constructs specific for antigens that are expressed in hematological malignancies (tool CAR_123_ and tool CAR_22_, which are specific for CD123 and CD22, respectively) were used to assess the robustness of our engineering strategy. Control samples transduced with each single repair matrix after TALEN transfection were included to accurately identify gene-edited cell subpopulations using flow cytometry. TALEN efficiently co-inactivated the TRAC and B2M genes with up to 96% of TCRαβ(−) HLA-ABC(−) T cells obtained in the presence of both repair matrices (Fig. 1c, top panel, black box, Fig. 1d black box plots, median ~90%). CAR and HLA-E expression were observed in TCRαβ(−) HLA-ABC(−) T cells (Fig. 1c, red or green boxes, respectively, Fig. 1d, red or green box plot, respectively) indicating successful disruptive insertion of both transgenes at TRAC and B2M loci. The efficiency of double targeted insertion reached as much as 68% with the two different CAR constructs (Fig. 1c blue box and 1d, blue box plots, median ~50%). Therefore, this TALEN/AAV6-mediated editing strategy enables the efficient expression of CAR and HLA-E by TCRαβ(−) HLA-ABC(−) cells after a single-transfection/transduction step. For the sake of clarity, we refer to this engineered T-cell scaffold as ΔTRAC_CAR_ΔB2M_HLAE_ in the following sections. Of note, similar levels of CAR expression and TRAC and B2M inactivations were reached using a CAR construct vectorized by recombinant lentivirus particle (rlv, Supplementary Fig. 1, TLA sample 8).

### Specificity of TALEN- and AAV6-mediated engineering of ΔTRAC_CAR_ΔB2M_HLAE_

To investigate the specificity of TALEN cleavage and AAV6 matrix insertion, we performed an oligo capture assay (OCA)^23^ and targeted locus amplification (TLA)^24^ analysis of engineered T cells. These methods enable unbiased identification of off-site TALEN activity and of the integration sites of CAR_m_ and HLA-E_m_. We also quantified translocations between B2M and TRAC loci by qPCR. OCA analysis of TRAC and B2M TALEN co-treated T cells identified candidate off-target sites that were then validated or invalidated using high-throughput DNA sequencing of amplicon-specific PCRs (Supplementary Fig. 1a and Supplementary Table 1). High-throughput DNA sequencing of the 22 top-scoring candidate off-target sites showed insertions/deletions (indels) frequencies falling under the threshold of relevant detection (threshold = 0.16, see “Methods”), indicating that the TRAC and B2M TALEN co-treatment does not promote significant off-site targeting. As expected^2,25^, simultaneous transfection of T cell by both TALEN, promoted translocation between the TRAC and B2M loci in up to 4% of cells (T2-TCR centromeric, Supplementary Fig. 1b and Supplementary Tables 3 and 4). When the CAR_m_ and HLA-E_m_ AAV6 matrices were added to the TALEN treatment, translocations were detected at a level similar to that observed in mock-treated T cells. Because our qPCR method could not amplify translocation events containing the HLA-E_m_ or CAR_m_ matrices, we cannot draw conclusions about the presence of translocation events integrating all or part of the AAV6 payload.

TLA analysis of engineered T cells with the double insertion of CAR_m_ and HLA-E_m_ at the TRAC and B2M loci showed that the transgenes were precisely inserted at their proper locations (CAR_m_ at chr14 and HLA-E_m_ at chr15, Supplementary Fig. 1d, f, Sample 7, red boxes), as reported earlier with similar editing strategies^23,26,27^. We also observed rare CAR_m_ and HLA-E_m_ integrations at chr15 and ch14, respectively (CAR_m_ at chr15 and HLA-E_m_ at chr14, Supplementary Fig. 1d,f, Sample 7, blue box). This suggests that a small number of integrations arose from the homology-independent insertion of AAV6 matrices, as documented in former reports^23,26,27^. Notably, such homology-independent insertion events were found to occur at markedly lower rates than those observed in control experiments performed with a single AAV6 matrix and an unpaired TALEN (CAR_m_ with B2M TALEN or HLA-E_m_ and TRAC TALEN, compare the blue boxes in Sample 2 or 4 from Supplementary Fig. 1c, f to Sample 7 from Supplementary Fig. 1d). Of note, homology-independent insertion events obtained with AAV6 were found to occur at markedly lower rates than those obtained using CAR construct control vectorized by lentivirus particles along with B2M and TRAC TALEN co-transfection (Compare blue boxes in sample 7 to sample 8 Supplementary Fig. 1d, e).

### ΔTRAC_CAR_ΔB2M_HLAE_ cells display antitumor activity in vivo and in vitro

**Fig. 2:**
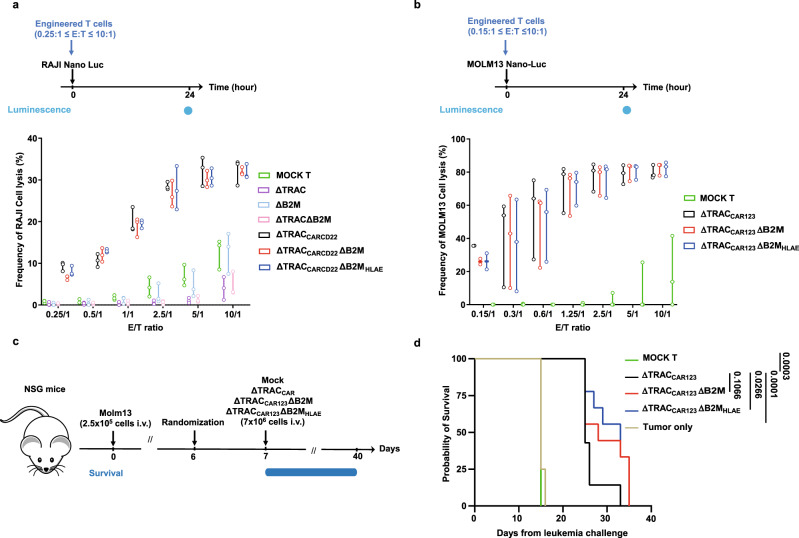
The disruptive targeted insertion of HLA-E at the B2M locus does not affect the antitumor activity of ΔTRAC_CAR_ΔB2M_HLAE_. **a**, **b** Antitumor activity of engineered T cells in vitro. T cells edited as indicated and expressing a CAR specific for either CD22 (**a**) or CD123 (**b**) were co-cultivated in vitro, with nano-luciferase-expressing RAJI or MOLM13 leukemia cell lines, respectively, using variable effector to target (E/T) ratios. The nano-luciferase signal obtained after co-culture was normalized to the one obtained for tumor cells fully depleted by Zwittergent^®^ to determine the frequency of tumor cells lysis. On each box plot, the central mark indicates the median, the bottom and top edges of the box indicate the interquartile range (IQR), and the whiskers represent the maximum and minimum data point. Each point represents one experiment performed with a given T-cell donor (*n* = 3 biological replicate for CAR T-cell specific for CD22 and CD123, *n* = 3 and *n* = 1 technical replicates for CAR T-cell-specific CD22 and CD123, respectively). *p*-values are documented in the source file for clarity. **c** Schematic showing the experimental design to investigate the antitumor activity of ΔTRAC_CAR123_ΔB2M_HLAE_ in the xenograft mouse model. Immunodeficient NSG mice were adoptively transferred with MOLM13-Luc-GFP leukemia cells (2.5 × 10^5^ cells/mouse in 100 μL of PBS via i.v.). On day 7, leukemia-bearing mice were adoptively transferred (i.v.) to randomized mice with mock-transduced T cells (*n* = 7), ΔTRAC_CAR123_T cells (*n* = 7), ΔTRAC_CAR123_ΔB2M T cells (*n* = 9), ΔTRAC_CAR123_ΔB2M_HLAE_ T cells (*n* = 9) or no T cells (*n* = 8) (7 × 10^6^ viable CAR^+^ cells/mouse in 100 μL of PBS via i.v. injection). Mice were monitored and followed for overall survival. **d** Kaplan–Meier plot obtained for different mouse cohorts. Log-rank (Mantel–Cox) tests were used for statistical analysis. *P* values are indicated on the figures. Source data are provided as a Source Data file.

To investigate the impact of B2M depletion and HLA-E expression on CAR T-cell antitumor activity^28^, we first determined whether CAR T cells engineered with these changes showed cytotoxic activity against leukemia cell lines in vitro. Engineered CAR T cells specific for CD22 or CD123 showed similar antitumor activity toward their respective leukemia cell line targets (RAJI and MOLM13 tumor cells, respectively, Fig. 2a, b) regardless of the number of edited features (ΔTRAC_CAR_, ΔTRAC_CAR_ΔB2M, and ΔTRAC_CAR_ΔB2M_HLAE_). This activity was found significantly higher than MOCK control cells lacking CAR expression. By design, the background antitumor activity of ΔTRAC and ΔTRACΔB2M toward RAJI cells was significantly lower than Mock and ΔB2M control groups, in agreement with the low, nonspecific and TCR dependent-cytolytic activity of T cells (Fig. 2a). We then evaluated the antitumor activity of TRAC_CAR123_ΔB2M_HLAE_ T cells in vivo using MOLM13 tumors xenografted in immunodeficient NSG mice (Fig. 2c). A single administration of ΔTRAC_CAR123_ΔB2M_HLAE_ T cells led to significantly extended survival in leukemia-bearing mice (Fig. 2d). Consistent with our in vitro results in the absence of host T cells and NK cells, we observed no significant difference in overall survival between mice treated with ΔTRAC_CAR123_ T cells and mice treated with ΔTRAC_CAR123_ΔB2M T cells or between those treated with ΔTRAC_CAR123_ΔB2M T cells and ΔTRAC_CAR123_ΔB2M_HLAE_ T cells. Altogether, these in vitro and in vivo results indicate that neither the inactivation of B2M nor the disruptive targeted insertion of HLA-E at the B2M locus affect the antitumor activity of engineered CAR T cells. This conclusion was reached with two different tool CAR constructs indicating that our engineering process could be used with other CAR constructs including those used in the clinic.

### Targeted knock-out of the B2M locus enables engineered T cells to resist alloresponsive T-cell attacks

We next verified that the depletion of HLA-ABC from the surface of engineered T cells prevented their elimination by alloresponsive T cells. Alloresponsive T cells primed for 3 weeks to recognize and attack T cells from three different donors were used to perform mixed lymphocyte reactions (MLR) with TRAC and B2M TALEN-engineered T cells (Supplementary Fig. 2a). T cells were intentionally engineered to obtain a mixture of engineered T-cell subpopulations (~50% HLA-ABC(−) and 50% HLA-ABC(+)) and to assess their relative sensitivity to alloresponsive T-cell attack within each sample. The HLA-ABC(−) subpopulation of engineered T cells (Supplementary Fig. 2b) was significantly enriched by up to 23-fold over the HLA-ABC(+) subpopulation in the presence of alloresponsive T cells in MLR assays (Supplementary Fig. 2c). Consistent with former reports^12,13^, these results indicate that the HLA-ABC(−) subpopulation is resistant to allogeneic T-cell-mediated cytolytic attack and confirms the hypoimmunogenic properties of HLA-ABC(−) cellular scaffold.

### Targeted insertion of HLA-E at the B2M locus enables engineered ΔTRAC_CAR_ΔB2M_HLAE_ to resist NK-cell attacks in vitro

**Fig. 3:**
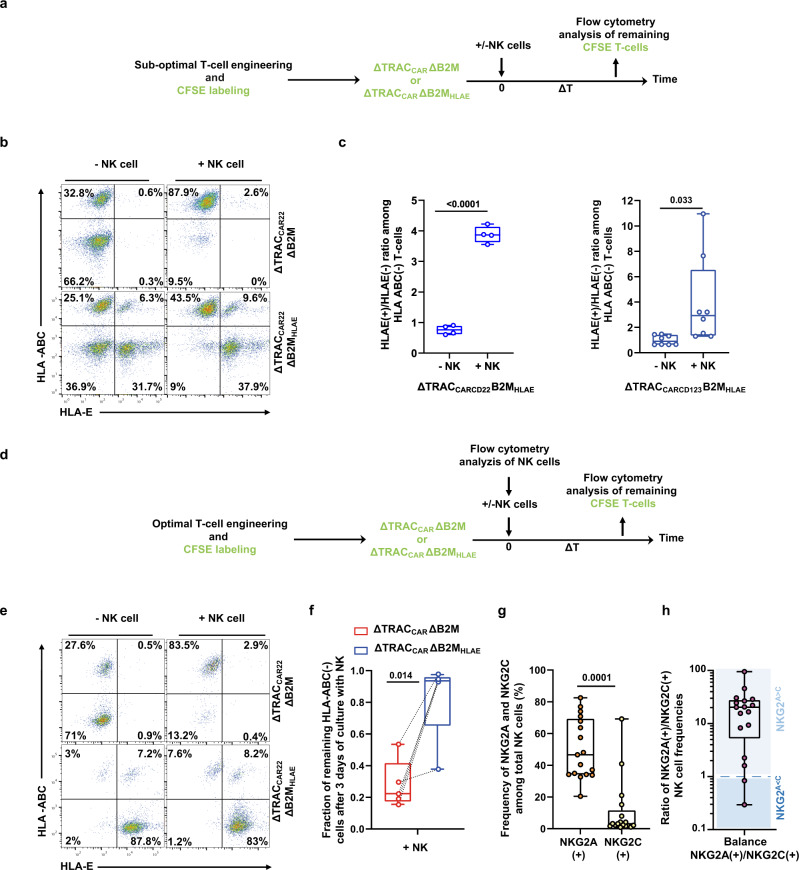
Targeted insertion of HLA-E at the B2M locus efficiently prevents NK-cell-mediated depletion of ΔTRAC_CAR_ΔB2M_HLAE_ in vitro. **a** Schematic showing the experimental design to investigate the susceptibility of ΔTRAC_CAR_ΔB2M_HLAE_ cells, engineered under suboptimal conditions, to NK-cell-dependent depletion in vitro. **b** Representative flow-cytometry plots showing the frequency of HLA-ABC(+)/(−) and HLA-E(+)/(−) subpopulations of ΔTRAC_CAR22_ΔB2M and ΔTRAC_CAR22_ΔB2M_HLAE_ T cells obtained after co-culture. **c** Box plots representing the ratio of HLA-E(+) to HLA-E(−) computed from the remaining HLA-ABC(−) subpopulation of ΔTRAC_CAR_ΔB2M_HLAE_ T cells cultured with or without NK cells; ΔTRAC_CAR22_ΔB2M_HLAE_ T cells (left panel, T-cell donor (*n* = 2) and NK-cell donor (*n* = 1)); ΔTRAC_CAR123_ΔB2M_HLAE_ T cells (right panel, T-cell donor (*n* = 2) and NK-cell donor (*n* = 4)). Each point in the box plot represents one experiment performed independently with a given T-cell donor and NK-cell donor. **d** Schematic showing the experimental design to investigate the susceptibility of ΔTRAC_CAR_ΔB2M_HLAE_ cells, engineered using optimal conditions, to NK-cell-dependent depletion in vitro. **e** Representative flow-cytometry plots showing the frequency of HLA-ABC(+)/(−) and HLA-E(+)/(−) subpopulations of ΔTRAC_CAR22_ΔB2M and ΔTRAC_CAR22_ΔB2M_HLAE_ T cells obtained after co-culture. **f** Box plots representing the fraction of HLA-ABC(−) cells of ΔTRAC_CAR22_ΔB2M and ΔTRAC_CAR22_ΔB2M_HLAE_ T cells remaining after co-culture (*n* = 5 NK donors, *n* = 1 T-cell donor with two technical duplicates averaged). **g** Box plot representing the frequencies of NKG2A(+), NKG2C(+) cells detected by flow cytometry in healthy NK-cell donors (*n* = 17). **h** Box plot representing the ratio of NKG2A(+)/NKG2C(+) cell frequencies detected by flow cytometry in healthy donors (*n* = 17). NKG2^A>C^ and NKG2^C>A^ donors are shown in the light-blue and dark-blue fields, respectively. In each box plot, the central mark indicates the median, the bottom and top edges of the box indicate the interquartile range (IQR), and the whiskers represent the maximum and minimum data point. Two-tailed paired *t* test with a confidence interval of 95% was used to compute statistics for the dataset illustrated in (**c**, **f**, **g**). *P* values are indicated in the figures. Source data are provided as a Source Data file.

To assess whether HLA-E expression can prevent the depletion of HLA-ABC deficient T cells by NK cells, we compared the sensitivity of ΔTRAC_CAR22_ΔB2M and ΔTRAC_CAR22_ΔB2M_HLAE_ cells to the cytotoxic activity of healthy donor NK cells in vitro (Fig. 3a). First, as a proof of principle, we intentionally engineered T cells in suboptimal conditions to obtain balanced engineered CAR T-cell subpopulations (HLA-ABC(−)/(+) and HLA-E(−)/(+)). This allowed us to assess the relative sensitivity of these subpopulations to NK-cell attack. After 3 days in culture, HLA-ABC(−) HLA-E(−) T cells were selectively and markedly depleted by NK cells in both ΔTRAC_CAR22_ΔB2M T cells and ΔTRAC_CAR22_ΔB2M_HLAE_ T cells, consistent with the missing self-mediated activation of NK cells (Fig. 3b). Such depletion was correlated with significant enrichment of the HLA-ABC(−) HLA-E(+) T-cell subpopulation of ΔTRAC_CAR22_ΔB2M_HLAE_ T cells, as demonstrated by a fourfold increase in the HLA-E(+) to HLA-E(−) ratio among HLA-ABC(−) T cells compared to untreated control (Fig. 3c, left panel). This result indicates that the expression of HLA-E at the surface of T cell successfully inhibited the cytolytic activity of NK cells. HLA-E(+) T cells were also enriched among HLA-ABC(−) T cells when the CD123 tool CAR was substituted for a CD22 tool CAR (about a threefold increase, ΔTRAC_CAR123_ΔB2M and ΔTRAC_CAR123_ΔB2M_HLAE_, Fig. 3c, right panel), confirming the protective role of HLA-E against NK-cell activity and demonstrating the transposable nature of this feature. It is to be noted that the values of the HLA-E(+)/HLA-E(−) ratio is more dispersed in the dataset for CD123 CAR, possibly due to a larger dataset (8 points vs. 4 points) where the variability in the cytolytic activity of NK cells is more likely to be observed from donor to donor.

To further confirm that HLA-E expression efficiently protects HLA-ABC-deficient T cells from NK-cell attack, we next engineered T cells to obtain optimal HLA-ABC inactivation and HLA-E expression (Fig. 3d). The resulting ΔTRAC_CAR22_ΔB2M_HLAE_, harboring a majority of HLA-ABC(−)/HLA-E(+) subpopulation (~90%) were then co-cultured with NK cells for 3 days and the remaining HLA-ABC(−) subpopulation was quantified. Consistent with the data described in Fig. 3b, c, the HLA-ABC(−) subpopulation of ΔTRAC_CAR22_ΔB2M control T cells was significantly depleted (80% depletion compared to untreated control). The extent of this depletion was variable, suggesting that some NK-cell donors may be tolerant toward HLA-ABC(−) HLA-E(−) T cells. In stark contrast, the HLA-ABC(−) subpopulation of ΔTRAC_CAR22_ΔB2M_HLAE_ T cells remained resistant to NK-cell attack, confirming the protective role of HLA-E (Fig. 3e, f and supplementary Fig. 3). As expected^29,30^, ΔTRAC_CAR22_ΔB2M_HLAE_ T cells were not killed by NK cells harvested from donors with more NKG2A(+) NK cells than NKG2C(+) NK cells (NKG2^A>C^ donors, see Supplementary Fig. 3, donor 1, 3, 4, and 5), but from those harboring more NKG2C(+) NK cells than NKG2A(+) NK cells (NKG2^C>A^ donors, Supplementary Fig. 3, donor 2). This trend was confirmed by investigating the level of CD107a/b degranulation of total NK cells, NKG2A(+) NK cells or NKG2C(+) NK cells subpopulations after being co-cultivated with ΔTRAC_CAR_ΔB2M_HLAE_ (Supplementary Fig. 4). This phenomenon is consistent with the dual specificity of HLA-E for NKG2C and NKG2A, two orthogonal surface-exposed NK receptor that are known to activate and inhibit NK cytotoxic activity, respectively, upon HLA-E engagement^31,32^. Noteworthy, NKG2^A>C^ donors (*n* = 15 out 17, 88%, Fig. 3g, h) were significantly more prevalent than NKG2^C>A^ donors (*n* = 2 out 17, 12%, Fig. 3g, h), suggesting that ΔTRAC_CAR_ΔB2M_HLAE_ T cells are widely hypoimmunogenic toward healthy donor NK cells.

### Targeted insertion of HLA-E at the B2M locus prolongs the antitumor activity engineered ΔTRAC_CAR_ΔB2M_HLAE_ in the presence of activated NK cell in vitro

**Fig. 4:**
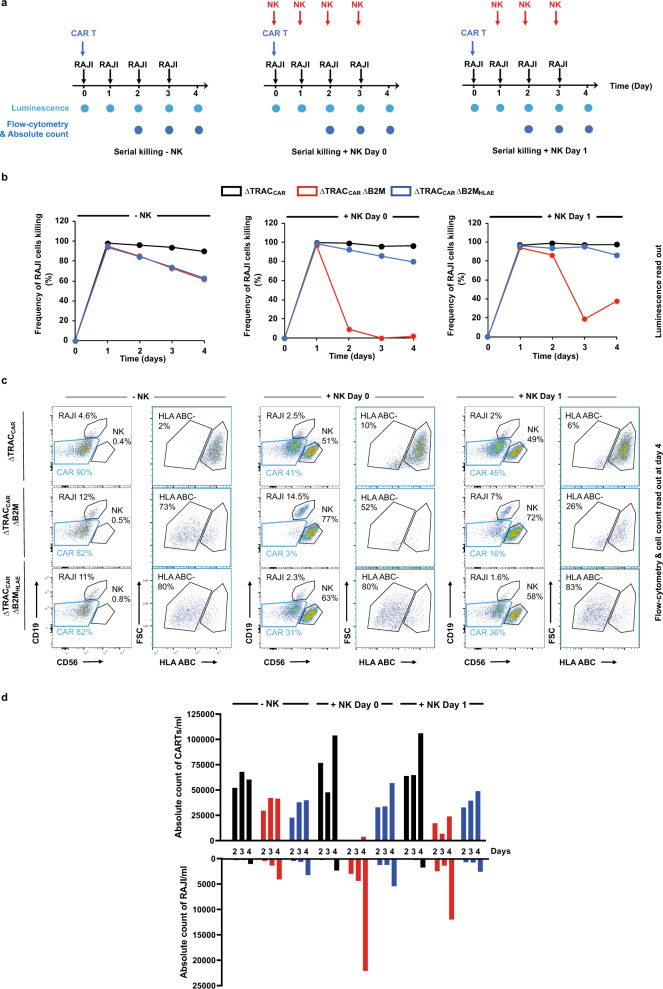
Targeted insertion of HLA-E at the B2M locus enables the efficient and prolonged antitumor activity of ΔTRAC_CAR_ΔB2M_HLAE_ in the presence of activated NK cells in vitro. **a** Schematic showing the serial killing assay designed to investigate the long-term antitumor activity of ΔTRAC_CAR22_ΔB2M_HLAE_ cells toward RAJI-Luc cells in the presence or absence of activated NK cells. The three different serial killing assay scenarios investigated are illustrated (no NK, NK day 0, and NK day 1). Black, blue, and red arrows indicate addition of RAJI cells, CAR T cells (ΔTRAC_CAR22_, ΔTRAC_CAR22_ΔB2M and ΔTRAC_CAR22_ΔB2M_HLAE,_ *n* = 1 donor) and NK cells (*n* = 1 donor), respectively. Luminescence and flow-cytometry analysis of cell populations are indicated at the different measurement time points. **b** Frequency of RAJI-Luc cells killing observed by luminescence with the three different serial killing scenarios performed with a CAR T cell to RAJI ratio of 2.5:1 and NK to CAR T-cell ratio of 1:1. Each point represents one experiment performed with a given T-cell donor and a given NK donor. **c** Representative flow-cytometry plots showing the different cell populations remaining at the end of the serial killing assay in absence of NK cell (left panel) and in the presence of NK cells added at day 0 and at day 1 (middle and right panel, respectively). The gating strategy is indicated by blue boxes. **d** Absolute CAR T cells and RAJI cells counts obtained by flow-cytometry analysis and cell counts in the same condition as in (**b**). Source data are provided as a Source Data file.

We demonstrated in two independent sets of experiments that targeted insertion of HLA-E at the B2M locus did not affect the antitumor activity of ΔTRAC_CAR_ΔB2M_HLAE_ and allowed them to resist to NK-cell attack. We then sought to test if these two properties held true when CAR T cell were repeatedly co-challenged by tumor and NK cells (Fig. 4). To do so, we set up a serial killing assay where the antitumor activity of the three different engineered versions of CAR T cell (ΔTRAC_CAR22_, ΔTRAC_CAR22_ΔB2M, ΔTRAC_CAR22_ΔB2M_HLAE_) were challenged over 4 days, by daily addition of RAJI and NK cells (NKG2^A>C^ donor, Fig. 4a). Two scenarios of NK-cell addition were investigated (+NK day 0 or +NK day 1) to mimic the different physiological conditions that may be encountered by CAR T cell following patients’ preconditioning. Our results showed that while ΔTRAC_CAR_ΔB2M and ΔTRAC_CAR_ΔB2M_HLAE_ showed similar antitumor activity in the absence of NK cells (Fig. 4b, left panel), daily addition of NK led to complete or partial abolition of ΔTRAC_CAR_ΔB2M activity (Fig. 4b, middle and right panels, respectively). In stark contrast, ΔTRAC_CAR_ΔB2M_HLAE_ remained highly active and behaved similarly to ΔTRAC_CAR_. By design, the NK-dependent drop of ΔTRAC_CAR_ΔB2M activity was correlated to a marked decrease CAR T-cell counts and to an increase of RAJI cell counts (Fig. 4c, d). Further analysis of the cell populations remaining at the end of the serial killing assay showed that the B2M(−) subpopulation of ΔTRAC_CAR_ΔB2M was selectively depleted by NK cells while remaining constant in the case of ΔTRAC_CAR_ΔB2M_HLAE_. Similar observations were made with different CAR to RAJI and CAR to NK-cell ratio (Supplementary Fig. 5). Taken together, these results indicate that the targeted insertion of HLA-E at the B2M locus prolongs the antitumor activity of engineered ΔTRAC_CAR_ΔB2M_HLAE_ in the presence of cytotoxic levels of activated NK cell.

### NK cells from AML/ALL patients and healthy donors display similar phenotypical characteristics

**Fig. 5:**
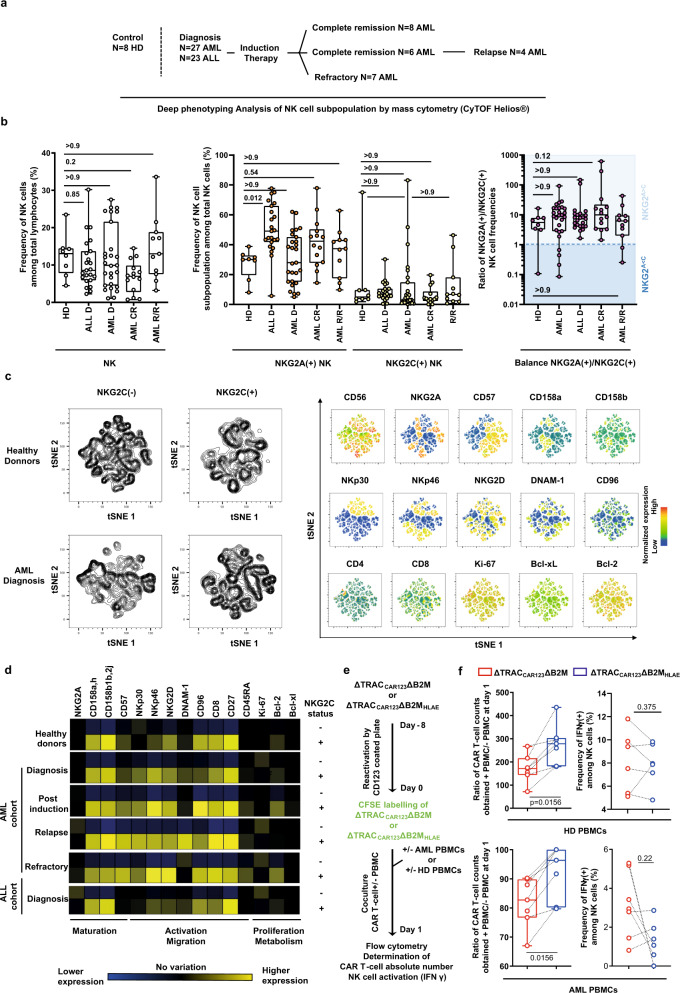
Deep phenotyping and functional characterization of NK cells from healthy donors and AML or ALL patients undergoing standard induction chemotherapy. **a** Total lymphocytes obtained from healthy donors (HD, *n* = 8), newly diagnosed ALL patients (ALL D, *n* = 23) and AML patients selected at the time of diagnosis (AML D, *n* = 27), complete remission (AML CR, *n* = 8), and in relapse/refractory status (AML R/R, *n* = 11) were characterized using mass cytometry. **b** Frequencies of NK-cell subsets among total lymphocytes (left panel), frequencies of NKG2A(+) and NKG2C(+) subpopulations among total NK-cell subset (middle panel), and ratio of NKG2A(+) over NKG2C(+) subpopulations (right panel) characterized by mass cytometry. For quantitative comparisons, data were analyzed using an ordinary one-way ANOVA test, a tukey’s multiple comparison test with single pooled variance and a confidence interval of 95%. **c**, **d** For each group described in **a**, NKG2C(−) and NKG2C(+) NK cells from each individual were exported and concatenated in order to generate consensus files. **c** The optimized parameters for T-distributed stochastic neighbor embedding (opt-SNE) algorithm were used to cluster NK-cell populations based on the expression of markers of interest in healthy donors and in AML patients at the time of diagnosis (left panel). Expression of markers of interest defining the different clusters are projected on opt-SNE maps (right panel). **d** The heatmap displays the mean frequencies of NK-cell markers in NKG2C(−) and NKG2C(+) NK cells, relative to pooled NKG2C(−) and NKG2C(+) NK cells. **e** Schematic showing the experimental design to investigate the susceptibility of ΔTRAC_CAR123_ΔB2M and ΔTRAC_CAR123_ΔB2M_HLAE_ cells (*n* = 1 T-cell donor), engineered using optimal conditions, to NK-cell-dependent depletion in vitro using PBMC from healthy donors (*n* = 7) AML patient (*n* = 7) at diagnostic. **f** Box plots represent the ratio of ΔTRAC_CAR123_ΔB2M and ΔTRAC_CAR123_ΔB2M_HLAE_ counts obtained in the presence of PBMCs over the counts obtained in the absence of PBMCs. Dotted lines indicate data obtained from the same PBMC donor. In each box plot, the central mark indicates the median, the bottom and top edges of the box indicate the interquartile range (IQR), and the whiskers represent the maximum and minimum data point. For quantitative comparisons, data were analyzed using a two-sided non-parametric Wilcoxon matched-pairs signed-rank test with a confidence interval of 95%. *P* values are indicated on the figures. Source data are provided as a Source Data file.

Cancer patient NK cells may display different fitness and cytotoxic activity compared to healthy donor NK cells. Thus, their ability to attack and deplete the ΔTRAC_CAR_ΔB2M_HLAE_ scaffold should be evaluated to assess the robustness and potential clinical translatability of the B2M/HLA-E engineering embedded in this scaffold. To gauge the magnitude of this attack, we further investigated the phenotype, fitness, and cytotoxicity of clinically relevant primary NK cells obtained from several ALL and AML patients. Because NK-cell numbers and fitness can be affected by previous treatment and by the nature and stage of each disease, we selected patient samples obtained before and after conventional frontline therapy. We first selected a cohort 23 ALL patients and 27 AML patients treated with induction therapy (7 + 3 anthracycline, cytarabine), and thoroughly investigated the phenotype of their NK-cell subpopulations using mass cytometry (Fig. 5a).

The frequencies (Fig. 5b, left panel) and absolute counts (Supplementary Fig. 6) of NK cells were comparable between the different cohorts. One exception could be however noticed for AML patients in complete remission (AML CR), who displayed a significantly lower frequency of total NK cells (Fig. 5b, left panel) and median absolute counts that fell under the normal range observed in healthy donors (Supplementary Fig. 6b). Frequencies of NKG2A(+) and NKG2C(+) NK-cell subpopulations were also comparable between the different cohorts (except for newly diagnosed ALL patients, *P* value <0.0001) with a majority of patient being NKG2^A>C^ (92% of patients observed across diagnostic, complete remission and relapse/refractory status were NKG2^A>C^; Fig. 5b, middle and right panels). Therefore, these results suggest that HLA-E expression at the surface of ΔTRAC_CAR_ΔB2M_HLAE_ T cells is likely to inhibit NK cells from most ALL and AML patients, regardless of the stage of their disease.

As described earlier for healthy donors (Fig. 3g and Supplementary Fig. 3b), some NKG2^C>A^ outlier patients could be identified in the different cohorts studied. Indeed, these patients were identified in 4 out of 27 (15%) and 2 out of 12 (17%) of newly diagnosed and relapsed refractory AML patients, respectively (Fig. 5b, right panel). Because we observed that NK cells from NKG2^C>A^ healthy donors depleted ΔTRAC_CAR_ΔB2M_HLAE_ T cells (Fig. 3 and Supplementary Fig. 3), we hypothesized that NKG2^C>A^ patient NK cells would exhibit similar behavior.

To predict the cytolytic activity of NKG2^C>A^ patient NK cells toward ΔTRAC_CAR_ΔB2M_HLAE_ T cells, we further dissected the phenotypic characteristics of their NKG2C(+) NK cells. To do so, we investigated the fitness of NKG2C(+) NK-cell subpopulations by quantitatively analyzing their maturation, activation/migration, and proliferation markers (Fig. 5c, d). Deep phenotyping analysis performed on several extracellular and intracellular relevant markers, indicated that the NKG2C(+) subpopulation was similar in overall fitness and maturation in AML and ALL patients compared to healthy donors. Indeed, we observed conventional expression of CD56, NKG2A, KIRs, CD57, and of the triggering receptors NKp30, NKp46, and NKG2D (Fig. 5d). These triggering receptors were markedly more prevalent in NKG2C(+) NK cells than in NKG2C(−) NK cells, used here as a control subpopulation. The NKG2C(−) control subpopulation was altered in newly diagnosed AML patients, as evidenced by the lower expression of NKP30, NKP46, and NKG2D activating receptors compared to healthy donors (Fig. 4c, left panel; compare the density plots of a healthy donor and AML patients). This trend was consistent with a former report^33^, validating the robustness and reproducibility of our dataset. Finally, we observed no difference in the intrinsic fitness and maturation profile of NKG2C(+) cells in both AML and ALL cohorts after chemotherapy, suggesting that NKG2C(+) NK cells lack the classical changes described in individuals undergoing chemotherapy (Fig. 5c)^33^. Altogether, our deep phenotyping results indicate that NKG2^C>A^ outlier patients may be equipped to deplete ΔTRAC_CAR_ΔB2M_HLAE_ T cells, although they represent a minority among the studied cohorts.

To validate our phenotypical investigation, we assessed the cytolytic activity of NK cells from AML patients against ΔTRAC_CAR123_ΔB2M_HLAE_. To do so, we co-cultivated ΔTRAC_CAR123_ΔB2M_HLAE_ or ΔTRAC_CAR123_ΔB2M with or without human peripheral blood mononuclear cells (PBMCs) from healthy donors and AML patients for 24 h and determined their absolute number at the end of the culture (Fig. 5e). Our results showed that ΔTRAC_CAR123_ΔB2M_HLAE_ was significantly more enriched than ΔTRAC_CAR123_ΔB2M at the end of the co-culture (Fig. 5f). This HLA-E-driven enrichment was correlated with a decrease of NK-cell activation probed by IFNγ release, although the number of PBMCs specimens tested was too low to reach statistical significance. These results confirmed that HLA-E expression at the surface ΔTRAC_CAR123_ΔB2M_HLAE_ could inhibit the missing self-mediated cytolytic activity of NK cells from AML patients. Taken together, these findings confirm that ΔTRAC_CAR_ΔB2M_HLAE_ T cells are widely hypoimmunogenic toward primary NK cells isolated from multiple donors, including cancer patients.

### ΔTRAC_CAR_ΔB2M_HLAE_ T cells resist NK-cell attacks in the hIL-15 NOG mouse model

**Fig. 6:**
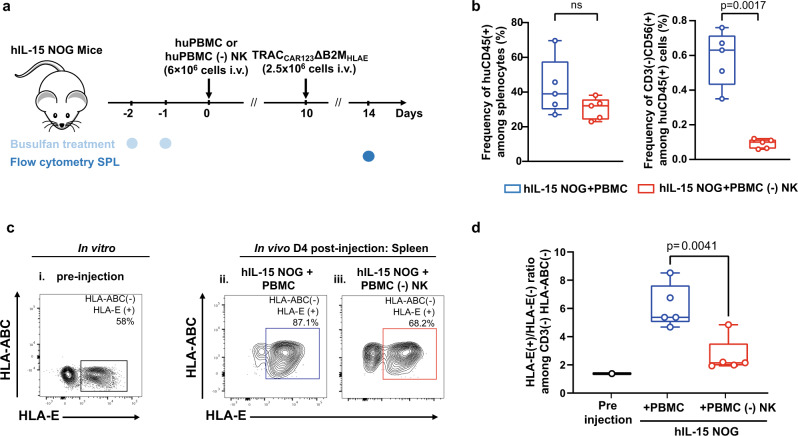
The targeted insertion of HLA-E at the B2M locus allows for efficient engraftment of ΔTRAC_CAR_ΔB2M_HLAE_ in hIL-15 NOG mice adoptively transferred with human NK cells. **a** Strategy for assessing the resistance of ΔTRAC_CAR123_ΔB2M_HLAE_ T cells to NK cells in a xenotransplantation model using hIL-15 NOG mice intravenously injected with human PBMCs. **b** Quantitation of human CD45^+^ immune cell and CD56^+^ NK-cell engraftment in the spleen of hIL-15 NOG mice injected with human PBMCs (*n* = 1 PBMC donor, *n* = 5 mice) or NK-depleted human PBMCs (*n* = 1 PBMC donor, *n* = 5 mice). **c** Representative flow-cytometry plots showing the expression of HLA-E by ΔTRAC_CAR123_ΔB2M_HLAE_ T cells at the time of injection and 4 days post intravenous injection in PBMC-engrafted hIL-15 NOG mice. **d** Box plots representing the ratio of HLA-E(+)/HLA-E(−) computed from the CD3(−)HLA-ABC(−) subpopulations observed in (**c**) (*n* = 5). For quantitative comparisons, data from (**b**, **d**), were analyzed using a two-sided parametric unpaired *t* test with a confidence interval of 95%. On each box plot, the central mark indicates the median, the bottom and top edges of the box indicate the interquartile range (IQR), and the whiskers represent the maximum and minimum data point. Each point represents the HLA-E(+)/HLA-E(−) among CD3(−)HLA-ABC(−) subpopulations obtain in one mouse. *P* values are indicated on the figures. Nonsignificant *P* values are indicated as “ns”. Source data are provided as a Source Data file.

To confirm the HLA-E inhibits NK cells in a more complex model, we evaluated the persistence and enrichment of ΔTRAC_CAR123_ΔB2M_HLAE_ in hIL-15 NOG mice engrafted with PBMCs from a healthy allogeneic donor. We first verified that this in vivo model supported efficient engraftment and expansion of human NK cells as described earlier^34^. To do so, we intravenously injected hIL-15 NOG mice with either PBMCs or PBMCs depleted of NK cells as negative control (Fig. 6a and Supplementary Fig. 7a). Flow cytometric analysis confirmed the successful engraftment of human CD45(+) immune cells and CD56(+) NK cells (Fig. 6b and Supplementary Fig. 7b) in agreement with data reported in ref. ^34^. We then confirmed that the mouse cohort injected with PBMCs partially depleted of NK cells displayed significantly lower NK-cell engraftment than did the cohort injected with PBMCs (Fig. 6b). As expected, this difference in NK levels did not impact the composition and engraftment of other immune cell compartments (Supplementary Fig. 7b).

In parallel, we engineered T cells from a different donor using suboptimal HLA-E knock-in conditions to generate ΔTRAC_CAR123_ΔB2M_HLAE_ cells with both HLA-ABC(−) HLA-E(−) and HLA-ABC(−) HLA-E(+) subpopulations (in an approximate ratio 1:1; Fig. 6c, i). This allowed us to assess the relative enrichment of HLA-E(+) over HLA-E(−) subpopulations in mice engrafted with allogeneic PBMCs. Four days after CAR T-cell engraftment, we observed an enrichment of HLA-ABC(−) HLA-E(+) ΔTRAC_CAR123_ΔB2M_HLAE_ cells in the spleen relative to their HLA-ABC(−) HLA-E(−) counterparts (Fig. 6c and Supplementary Fig. 7d). Quantification of this phenomenon translated into a fourfold increase of HLA(+)/HLA(−) CAR T-cell frequency ratio compared to the one observed pre-injection (Fig. 6d, compare black and blue circles). This enrichment was significantly lower in the mice engrafted with NK cells-depleted PBMCs (1.5-fold, Fig. 6d, compare black and red circles), indicating (i), that the depletion of HLA-ABC(−)HLA-E(−) subpopulation of CAR T cells is mediated by the cytolytic activity of engrafted NK cells and (ii), that HLA-E expression protects HLA-ABC(−) CAR T cells from NK cells attack, in agreement with the earlier results obtained in vitro.

Together, our results show the ΔTRAC_CAR_ΔB2M_HLAE_ can be generated by precise TALEN-mediated gene insertion of a CAR and a HLA-E construct at the TRAC and B2M loci in primary T cells. This process generates TCRαβ- and HLA-ABC-deficient engineered T cells expressing the CAR construct and the NK inhibitor HLA-E. Engineered CAR T cell displays immune-evasive properties toward alloreactive T cells and NK cells and shows efficient antitumor activity even in the presence of cytolytic levels of NK cells.

## Discussion

The goal of this work was to develop an immune-evasive universal CAR T-cell scaffold that is able to resist to both NK-cell and alloreactive T-cell attacks and that would be compatible with adoptive cell transfer in an allogeneic setting. Using a combination of TALEN-mediated gene editing and AAV6-dependent gene insertion, we developed a hypoimmunogenic universal CAR T-cell scaffold that we designated ΔTRAC_CAR_ΔB2M_HLAE_. ΔTRAC_CAR_ΔB2M_HLAE_ is devoid of TCRαβ and HLA Class-I expression and endowed with an engineered surface-exposed HLA-E. These three features enable CAR T cells to prevent GvH reaction and to evade the cytolytic activities from alloresponsive T cells and NK cells. While we confirmed that HLA-ABC(−) ΔTRAC_CAR_ΔB2M_HLAE_ overcame alloresponsive T-cell attack, we demonstrated their ability to evade NK cells attack by the significant enrichment of HLA-ABC(−) HLA-E(+) ΔTRAC_CAR_ΔB2M_HLAE_ in the presence of primary NK cells from healthy donors and AML patients. As a corollary, this feature enabled to prolong the antitumor activity of CAR T cells in the presence of supra stoichiometric levels of cytotoxic NK cells in vitro, corroborating the very recent results obtained with hypoimmunogenic T cells derived from iPS cells^35^. We also showed that ΔTRAC_CAR_ΔB2M_HLAE_ T cells exhibit antitumor activity in vivo, similar to that of ΔTRAC_CAR_ T cells, demonstrating that their additional hypoimmunogenic features do not affect their cytolytic functions. Finally, because NK cells from healthy donors display similar phenotypic and fitness characteristics to NK cells from AML patients and ALL patients, whatever the stage of their disease, we forecast that expression of HLA-E is likely to protect ΔTRAC_CAR_ΔB2M_HLAE_ from NK cells in the majority of AML and ALL patients.

Because of the complexity and exquisite efficacy of the human immune system, engineering hypoimmunogenic cellular scaffolds that would enable adoptive cell transfer in an allogeneic setting, has long been a daunting goal. However, the recent development of gene-editing tools and a growing understanding of allograft rejection mechanisms have enabled the emergence of different engineering solutions. For instance, hypoimmunogenic cellular scaffolds have been proposed in the field of human-induced pluripotent stem cell (hiPSC) and human embryonic stem cell (hESC) transplantation^17–19,36^. One of them consists of overexpressing PD-L1, HLA-G, and CD47 at the surface of pluripotent stem cells specifically depleted for HLA-A, HLA-B, HLA-C, and HLA class II^18,19^. This immune cloaking strategy was elegantly proven to efficiently prevent NK-cell-, T cell-, and macrophage-dependent depletion of engineered human pluripotent stem cells. These results were confirmed by a similar study showing that the overexpression of CD47 on the surface of induced pluripotent stem cells lacking HLA class-I and II surface receptors, allowed them to evade immune rejection^18^. Immune rejection of hESC was also efficiently prevented by the overexpression of PD-L1 and soluble CTLA4 immunoglobulin without relying on the inactivation of HLA class I and II^37^.

While these strategies are appealing and manageable for stem cell engineering, they require multiple iterative gene editing and antibiotic-dependent selection steps that are not well suited for the CAR T-cell manufacturing processes. In addition, the expression of certain cloaking factors including PD-L1, HLA-G, and others, may be detrimental to the cytolytic functions of CAR T cells, due in part, to these factors’ propensity to engage with inhibitory receptors (PD1 and ILT-2/4^38^) expressed at the surface of T cells. Furthermore, because they are tailored to maximize the long-term engraftment of pluripotent stem cells, these strategies aim at blocking all possible allograft rejection pathways including the T cell- (HLA class I and II/TCR), NK-cell- (missing HLA class-I/NKG2A) and macrophage-dependent (CD47) rejection of engineered cells. This high degree of hypoimmunogenicity may not be required for efficient CAR T-cell antitumor functions because transient CAR T-cell activity correlates with positive therapeutic outcome^39–43^. Furthermore, long-term engraftment of CAR T cells may be associated with different adverse events including the B-cell aplasia and bacterial infection usually observed in CD19 and CD22 autologous CAR T-cell therapies^44–46^. Thus, rather than engineering CAR T cells for long-term maintenance in allogenic settings, our strategy sought to extend CAR T-cell persistence by inhibiting the most important allograph rejection pathways.

Allograft rejection is mainly driven by CD8(+) T-cell, CD4(+) T cells, NK cells and, to a lesser extent, by macrophages^47^. However, in the context of CAR T-cell therapy, the relative contribution of these cell types to allograft rejection may vary depending on their absolute numbers and reconstitution kinetics following preconditioning regimen. Indeed, clinical data obtained from three independent patient cohorts (ALL and Large B-cell malignancies) showed significant differences in CD8(+), CD4(+) T cells, and NK-cell reconstitution kinetics following autologous CD19 CAR T-cell treatment and preconditioning regimen (Cyclophosphamide and fludarabine)^48–50^. Interestingly, CD8(+) T cells and NK cells quickly recovered to their initial levels in 3–4 weeks, whereas CD4(+) T cells showed a significantly slower recovery rate and, in some patients, had not returned to their initial level, 1 to 2 years after treatment onset. Thus, host CD8(+) T cells and NK cells are expected to play a key role in controlling the length of the allogeneic CAR T-cell therapeutic window by being the first and primary contributors to rejection.

Although CD8(+) T cells need to go through a clonal expansion to mount an efficient alloresponse, they may be the first subset to reject allogeneic CAR T cells. In this way, the inactivation of HLA class I at the surface of CAR T cells could efficiently blunt such rejection and offer an initial therapeutic window to eradicate cancer cells. The subsequent reconstitution of host NK cells may complexify this scenario by recognizing and depleting HLA class-I-deficient CAR T cells without the need for clonal expansion. Embedding an NK inhibitor within CAR T cells could further extend their persistence. We therefore focused on engineering a CAR T cell that could evade the cytolytic activities of NK cells and alloresponsive CD8(+) T cells. To achieve this goal, we aimed to prevent HLA Class-I expression in CAR T cells by inactivating B2M and to endow these cells with a surface-exposed NK inhibitor (HLA-E). We also considered the inactivation of TCRαβ to prevent GvHD, a major risk of allograft transplants. We reasoned that TRAC and B2M genes could be concomitantly inactivated and used as landing pads for transgene insertion (CAR and HLA-E). We forecasted that this strategy would simplify and speed up the CAR T-cell production process and mitigate potential genetic adverse events. The resulting cellular scaffold, ΔTRAC_CAR_ΔB2M_HLAE_, can be efficiently generated via a simultaneous double knockout by knock-in strategy. The combination of TRAC and B2M TALEN treatment along with an optimized design of AAV6 repair matrices enabled us to obtain a high frequency of TCRαβ(−) HLA-ABC(−) T cells expressing CAR and HLA-E. This engineering strategy is compatible with standard universal CAR T-cell production processes that already includes TCRαβ(+) T-cell depletion, does not require any HLA-E (+) CAR T-cell enrichment or antibiotic-dependent selection step and thus, appears suitable for clinical product manufacturing.

Co-treatment of T cells with TRAC and B2M TALEN did not result in significant off-site cleavage activity. This was demonstrated by an unbiased OCA assay and high-throughput DNA sequencing. As expected, this co-treatment induced translocations between Chr14 and Chr15, to an extent similar to the one reported previously with different gene-editing tools^2,25,51,52^. A search of gene fusion databases, including the Atlas of Genetics and Cytogenetics in Oncology and Hematology, did not show any record of pathogenic translocation between the TRAC and B2M loci (14q11 and 15q21.1, respectively), suggesting that serious adverse events associated with such gene fusion are unlikely. Although this limitation could be mitigated by further process development, consisting in uncoupling TRAC and B2M editing by two to three days, further preclinical studies should be carried out to assess the potential toxicity of such translocations before moving to clinical studies. In addition, the homology-dependent insertion of AAV6 repair matrices was specific to the TRAC and B2M loci and correlated with robust transgene expressions. Nevertheless, we found rare homology-independent insertions of HLA-E_m_ and CAR_m_ at the TRAC and B2M loci, respectively. Because both repair matrices are devoid of promoter, these improper insertions would not be expected to lead to transgene expression unless they inserted in frame with the edited gene. We did not observe CAR expression at the B2M locus (Supplementary Fig. 1c, TLA sample 4), but we did find low but detectable expression of HLA-E at the TRAC locus (Supplementary Fig. 1c, TLA sample 2). Although we do not envision critical adverse events related to HLA-E expression by the TRAC locus, this dataset points out one of the limitations of this multiplex gene-editing strategy that must be considered in the development of other cell therapy products. It is noteworthy that this AAV6-mediated insertion approach was markedly more specific than random transgene insertion mediated by lentivirus particles (Supplementary Fig. 1c, TLA sample 8).

We further report that efficiently targeted insertion of HLA-E_m_ at the B2M locus leads to a robust HLA-E surface expression that inhibits the cytolytic activity of NK cells. Consistent with former reports^16,22^, this outcome results from the structure and identity of the different domains of the engineered HLA-E. The inhibitory potency of HLA-E toward NK cells is intimately linked to the nature of the nonameric peptide it exposes. Its sequence was shown to significantly influence the binding affinity of HLA-E complex to the inhibitory receptor NKG2A and the activating receptor NKG2C, and thus to strongly alter the fine balance controlling NK activation and inhibition^20,21^. We chose to expose at the surface of the HLA-E engineered construct, the nonameric sequence VMAPRTLIL, because it promotes stronger engagement of NKG2A than NKG2C, as opposed to other conventional peptides including VMAPRTLFL (HLA-G-pep)^20–22^. By design, our in vitro results showed that our construct efficiently inhibits NKG2A(+) NK cells from multiple healthy donors and from AML patients. However, despite our choice of peptide, HLA-E can still activate NKG2C(+) NK cells, leading to the swift depletion of ΔTRAC_CAR_ΔB2M_HLAE_ T cells in NKG2^C>A^ donors. For the reasons described earlier, we believe that one way to mitigate this activation would be to identify a noncanonical nonameric peptide displaying orthogonal specificity for NKG2A and substitute it for VMAPRTLIL within the engineered HLA-E construct.

Nevertheless, in a clinical perspective, our results suggest that the HLA-E-mediated protection of ΔTRAC_CAR_ΔB2M_HLAE_ is likely to occur in the vast majority of AML and ALL patients. Indeed, most of these patients are NKG2^A>C^ (like healthy donors) and thus harbor a greater frequency of NKG2A(+) NK cells than NKG2C(+) NK cells. This imbalance, observed at different stages of disease (92% of NKG2^A>C^ patients observed across diagnostic, complete remission and relapse refractory status, Fig. 5b), is expected to promote evasion of ΔTRAC_CAR_ΔB2M_HLAE_ from NK cells and thus, potentially extend their overall persistence, although this needs to be demonstrated in clinical settings. Determining the NKG2A(+)/NKG2C(+) ratio prior to patients injection would be beneficial for fully exploiting the therapeutic potential of ΔTRAC_CAR_ΔB2M_HLAE_.

Expressing HLA-E on the surface of HLA-ABC(−) CAR T cells is not the only strategy to evade the cytolytic activity of NK cells. Indeed, one alternate approach would be to prevent the expression of key T-cell surface receptors involved in NK-cell activation. These receptors include MIC-A/B, SLAM family receptors CD48 and CD229, NKP46 ligand, B7H3 and CD155, different receptors known to activate NK cells through the engagement of their cognate receptors CD244, CD229, NKP46, IL20ra and KIR2DL5A, respectively^36,53–56^. Genetic abrogation or downregulation of these receptors may dampen the activation of NK cells triggered by the absence of MHC Class I. Although further work is needed to evaluate the robustness and clinical translatability of this approach, a recent report demonstrated that overexpression of the Human Herpes Virus-8 ubiquitin ligase E5 in K562 cell lines or T cells, could mitigate their depletion by NK cell through an hypothetical downregulation of MIC-A/B^57^. Another alternate strategy would be to endow engineered T cells with cytolytic activity toward NK cells. This strategy was elegantly explored by Mo et al*.*^58^ who recently showed that engineering CAR T cells with an alloimmune defense receptor (ADR) specific for 4-1BB, enabled them to efficiently blunt the HvG reaction by actively targeting activated NK cells and alloresponsive T cells.

The ultimate goal of a universal CAR T-cell product is to allow for efficient and specific depletion of cancer cells in allogeneic settings with minimal biological and toxicological footprints. Our strategy was designed to mitigate such footprints by allowing CAR T cells to evade the host immune system passively and locally without relying on active, systemic, and prolonged lymphodepletion. One potential advantage over the previously described strategies^2,9,58^ is to spare endogenous immune effectors and allow them to work in concert with CAR T cells in the fight against cancer cells. Such collaboration could be especially useful in the context of solid tumor treatments, where endogenous immune effectors, including tissue-resident memory cells^59,60^, tumor-infiltrating lymphocytes^61^ and other cellular subsets are already equipped and poised to deplete tumor antigen- and neoantigen-expressing cells. The maintenance of functional endogenous immune effectors could be also a key advantage to improve the potency of CAR T-cell therapies by allowing combination therapies with oncolytic viruses^62^, vaccine boosting agents^63^, bispecifc engagers^64^, or other antibody-based immunotherapies^65^. We believe ΔTRAC_CAR_ΔB2M_HLAE_ could allow for multiple relevant combination therapies that will leverage the full potential of the human immune system and improve the therapeutic outcome of adoptive cell therapies in allogeneic settings.

In summary, we report here the development of an immune-evasive universal CAR T-cell scaffold that is deficient for TCRαβ and HLA Class I and endowed with a surface-exposed HLA-E NK inhibitor. These features render it compatible with adoptive cell transfer in allogeneic settings by preventing GvHD and allowing it to evade the cytolytic activities of NK cells and alloresponsive CD8(+) T cells, the two major actors of HvG rejection. These hypoimmunogenic properties could potentially extend the persistence of universal CAR T cells and therefore, increase their antitumor potency in an immune-competent host, although it must be demonstrated in clinical settings. Our engineering strategy is efficient and specific, transportable to different CAR constructs and adaptable to conventional CAR T-cell manufacturing processes. We believe this next generation of universal CAR T cell has the potential to improve the therapeutic outcome of off-the-shelf therapeutic T-cell products and to allow their large-scale utilization against multiple malignancies for the benefit of a broader range of patients.

## Methods

### Materials

Cryopreserved human PBMCs were acquired from ALLCELLS (cat# PB006F). PBMCs were cultured in CTS OpTmizer media (obtained from Gibco, cat# A1048501), containing IL-2 (obtained from Miltenyi Biotec, cat# 130-097-748) or IL-7 and IL-15 (obtained from Miltenyi Biotec, cat#130-095-361 and #130-095-764), human serum AB (obtained from Seralab, cat# GEM-100-318), and CTS Immune Cell SR (obtained from Gibco, cat# A2596101). Human T Cell TransAct from Miltenyi Biotec (cat# 130-111-160) was used to activate T cells. Antibody staining was carried out with antibodies summarized in Supplementary Table 7. Luminescence of tumor cell lines was assessed in vitro using NANO-Glo and oneGlo reagents (Promega, cat# N1110 and cat# E6110, respectively) and in vivo using XenoLight D-luciferin (obtained from PerkinElmer, cat#770504). PBMCs from ALL and AML patients were obtained from the HEMATOBIO cohort (NCT02320656). PBMCs were cryopreserved in 90% albumin/10% DMSO. Samples of human origin and associated data were obtained from the IPC/CRCM/UMR 1068 Tumour Bank, that operates under the authorization # AC-2007-33 granted by the French Ministry of Research (Ministère de la Recherche et de l’Enseignement Supérieur). Prior to the scientific use of samples and data, patients were appropriately informed and asked to consent in writing, in compliance with French and European regulations. The project was approved by the IPC Institutional Review Board (Comité d’Orientation Stratégique, COS) as well as the Committee for the Protection of Persons South Mediterranean I (#2013-AO1437-38).

### Cell lines

MOLM13-nanoLuc-GFP and RAJI-Luc-GFP were engineered out of MOLM13 and RAJI cells (DSMZ, cat# ACC 554 and ATCC, cat# CCL-86, respectively) using an in house rLV encoding NanoLuc_T2A_EGFP construct and AMSbio cat# LVP323-PBS, respectively, using the manufacturer's protocols.

### Targeted integration of CAR and HLA-E constructs

Targeted insertion of CAR and HLA-E at the TRAC and B2M loci were performed as described previously^23^ with minor variations. Briefly, PBMCs were thawed, washed, resuspended, and cultivated in CTS OpTmizer complete media (reconstituted CTS OpTmizer, 5% human AB serum, 20 ng/mL IL-2). One day later, the cells were activated with Human T Cell TransAct (25 µL of beads/10^6^ CD3 positive cells) and cultivated at a density of 10^6^ cells/mL for 3 days in CTS OpTmizer complete media at 37 °C in the presence of 5% CO_2_. The cells were then split into fresh complete media and transduced/transfected the next day according to the following procedure. On the day of transduction-transfection, the cells were washed twice in Cytoporation buffer T (BTX Harvard Apparatus, Holliston, Massachusetts), and resuspended at a final concentration of 28 × 10^6^ cells/mL in the same solution. The cellular suspension (5 × 10^6^ cells) was mixed with 5 µg mRNA encoding each TRAC TALEN arm in the presence or absence of 5 µg of mRNA encoding each arm of B2M TALEN in a final volume of 180 µl. The cellular suspension was transfected in 0.4 cm^2^ cuvettes using Pulse Agile technology. The electroporation consisted of two 0.1 mS pulses at 3000 V/cm followed by four 0.2 mS pulses at 325 V/cm. Immediately after electroporation, T cells were transferred to a 12-well plate containing 2 mL of prewarmed CTS OpTmizer serum-free media and incubated at 37 °C for 15 min. Half of this cellular suspension was concentrated in 250 µL of the same media in the presence or absence of AAV6 particles (MOI = 2.5 × 10^5^ vg/cells) containing the CAR_m_ and/or HLA-E_m_ matrices and seeded in 48-well plates. After 2 h of culture at 30 °C, 250 µL of CTS OpTmizer media supplemented by 10% human AB serum and 40 ng/ml IL-2 was added to the cell suspension, and the mix was incubated overnight under the same culture conditions. The following day, cells were seeded at a density of 10^6^ cells/mL in complete CTS OpTmizer media and cultivated at 37 °C in the presence of 5% CO_2_. On day 6 after thawing, IL-2 was replaced with IL-7 and IL-15 (2800 IU/ml and 44 IU/ml final concentration, respectively). On day 8, the cells were resuspended in fresh complete medium supplemented with IL-7 and IL-15 (2800 IU/ml and 44 IU/ml final concentration, respectively) and 5% CTS Immune Cell SR. The cells were seeded in GREX10 at 0.125 × 10^6^ cell/ml and cultivated in the same media according to the manufacturer’s guidelines.

### Identification and detection of candidate off-site targeting by oligo capture assay and high-throughput DNA sequencing

Oligo capture assays (OCA)^23^ were used to assess the specificity of B2M and TRAC TALEN activity. Briefly, primary T cells were co-transfected with mRNA encoding TRAC and B2M TALEN and double-strand oligodeoxynucleotide (dsODN) and expanded for 6 days. Genomic DNA was recovered, sheared, end-repaired/A-tailed, processed, and analyzed by high-throughput DNA sequencing as described previously^23^. The resulting sequences were mapped onto the human genome (GRCh38) to identify potential off-site candidates. The frequency of insertion and deletion events (indels) generated at potential off-site candidates were then quantitatively assessed using high-throughput DNA sequencing of candidate off-site-specific PCR amplicons obtained from T cells co-treated with TRAC and B2M TALEN (without dsODN) and TCRαβ(−) cells enriched by TCRαβ negative purification as described previously^9^. Quantification of indels frequency shows background noise due to the non-negligible error rate of the Illumina sequencing process. This error rate varies depending on the sequencing run and on the sequence itself. To quantitatively assess this error rate, sequencing datasets from 647 TALEN assayed against non-relevant targets (TALEN-transfected) were retrieved from our internal database, as were the corresponding controls performed without TALEN (mock-transfected). The difference in indels frequency between the TALEN-treated samples and the control samples was computed for each dataset. These differences followed an approximately Gaussian distribution with a mean (m) of 0.0095%, and a standard deviation (σ) of 0.04922%. We defined an off-site candidate as confirmed when the difference in indels frequency of the TALEN- and Mock-transfected T cells was larger than a threshold (T) defined as T = m + 3 σ = 0.16%.

### Detection of translocations generated by simultaneous TRAC and B2M TALEN treatment in the presence or absence of CAR_m_ and HLA-E_m_ AAV6 matrices

Translocation events between the TRAC and B2M loci were detected via quantitative PCR using the translocation-specific primers described in Supplementary Table 4. Reactions were performed using PowerUp SYBR Green Master Mix (ThermoScicence Cat # A25742) and the Bio-rad CFX qPCR instrument (Bio-Rad) on genomic DNA extracted from each experimental group. Amplification efficiencies and copy numbers were determined using reference control matrices designed to mimic the expected translocation events (Supplementary Table 3). Genomic DNA and control matrices were quantified using PicoGreen dsDNA quantitation assay (Thermo Fisher). Within these matrices, an XhoI restriction site was introduced at breakpoints between B2M and TRAC TALEN target sites to control for potential contaminations of experimental samples from control matrices. The four potential translocations (T1–T4) were quantified in technical quadruplets, using sets of genomic DNA obtained from two independent T-cell donors. The two donors were either mock-treated (negative control), co-treated with mRNA encoding the TRAC and B2M TALEN (positive control), or co-treated with mRNA encoding the TRAC and B2M TALEN and transduced by AAV6 encoding CAR_m_ and HLA-E_m_.

### Targeted locus amplification assay

TLA analysis of engineered T cell was performed by Cergentis (Utrecht, Netherlands) as described^23,24^ using the engineered T-cell groups described in Supplementary Table 5 and Supplementary Fig. 1f and a primers described in Supplementary Table 6. Two or three specific primer sets were used for each engineered T-cells group. PCR products were purified, and library prepped using the Illumina Nextera flex protocol and sequenced on an Illumina sequencer. Reads were mapped using BWA-SW, version 0.7.15-r1140, settings bwasw -b 7. The NGS reads were aligned to the matrix sequences and host genome. Human genome build hg19 was used as a host reference genome sequence. Integration sites were detected based on a coverage peaks in the genome and on the identification of fusion-reads between the matrices sequence and the host genome as described in ref. ^23^.

### In vitro T-cell antitumor activity assay

The antitumor activity of the engineered T cells was assessed using cytotoxicity assays. Engineered T cells were mixed with a suspension of 5 × 10^4^ MOLM13-Nano-Luc or RAJI-Nano-Luc tumor cells at an effector to target ratio (E:T) ranging from 10:1 to 0.16:1 in a final volume of 0.1 mL of CTS OpTmizer media supplemented with 5% human AB serum. The mixture was incubated for 24 h, and cells were lysed using Zwittergent^®^ solution according to the manufacturer protocol. The luminescence of the remaining viable RAJI-Nano-Luc or MOLM13-Nano-luc cells was determined after incubating the cell lysate with NANO-Glo reagent at a 1:1 volume ratio for 3 min. The nano-luciferase signal obtained after co-culture was normalized to the one obtained for tumor cells fully depleted by Zwittergent^®^ to determine the frequency of tumor cells lysis reported in “Results”.

### In vitro NK-cell cytotoxicity assay with NK cells purified from healthy donors

After thawing, cryopreserved purified human CD56^+^ NK cells (AllCells) (1 × 10^6^ cells/mL) in a complete medium (NK MACS medium supplemented with 1% NK MACS supplement (Miltenyi Biotec) and 5% human AB serum) were cultured in a 24-well plate (500 μL/well) and incubated overnight at 37 °C, 5% CO_2_. On day 1, IL-2 (60 ng/mL) was added to NK cells. After an additional 5 h of incubation at 37 °C, 5% CO_2_, the NK cells were washed and placed in NK expansion medium (complete medium containing 40 ng/mL IL-2). Target cells (Mock-transduced T cells, ΔTRAC_CAR_ T cells, ΔTRAC_CAR_ΔB2M T cells, and ΔTRAC_CAR_ΔB2M_HLAE_ T cells), labeled with CFSE (1 mM) according to the manufacturer’s instructions, in NK expansion medium were co-cultured with IL-2 pretreated NK cells in a U-bottom 96-well plate (100 μL/well) at effector to target (5 × 10^4^ cells) ratios of 4:1 (CD123-specific CAR T cells) or 1:1 (CD22-specific CAR T cell) for 84 h (CD123-specific CAR T-cell) or 72 hrs (CD22-specific CAR T cells) at 37 °C, 5% CO_2_. Donor NK cells used in assays targeting CD22-specific CAR T cells were pre-activated with NK Activation/Expansion Kit (Miltenyi Biotec) according to the manufacturer’s instructions. Cells were then analyzed using flow cytometry. Data were expressed as the ratio of HLA-E(+) to HLA-E(−) subpopulations among remaining HLA-ABC(−) ΔTRAC_CAR_ΔB2M T cells (i.e., the frequency of HLA-E(+) T cells among total HLA-ABC(−) T cells/the frequency of HLA-E(−) T cells among total HLA-ABC(−) T cells).

### In vitro expansion and activation of NK cells used in the serial killing assay

To perform an in vitro serial killing assay in the presence of NK cells (see below), a large-scale activation and expansion of NK cells were set up according to the following method. After thawing, cryopreserved purified human CD56^+^ NK cells, negatively selected (AllCells) (5 × 10^6^ cells) were resuspended at 1 × 10^6^ cells/mL in expansion NK MACS medium (NK MACS medium supplemented with 1% NK MACS supplement (Miltenyi Biotec), 5% human AB serum, IL-2 (500 IU/mL), and IL-15 (140 IU/mL)). Cells were then cultured in a 24-well plate (700 μL/well) and incubated at 37 °C, 5% CO_2_ undisturbed for the first 5–6 days. At day 5 or 6, 300 μL expansion NK MACS medium was added without disturbing the cells. On day 7, a fresh expansion NK MACS medium was added to cells to dilute to a final concentration of 5 × 10^5^ cells/mL and cells were cultured in a six-well plate (2.5 mL/well). Starting on day 10, a fresh expansion NK MACS medium was added every 2 days to dilute cells to a final concentration of 5 × 10^5^ cells/mL and cells were cultured in T75 flasks (>7 mL/flask) or T175 flasks (>30 mL/flask). Expanded cells were utilized in serial killing assays starting at day 13 or 14.

### In vitro serial killing assay in the presence of activated NK cells

To assess the persistence of ΔTRAC_CAR_ΔB2M_HLAE_ T cells in vitro, a serial killing assay was performed. ΔTRAC_CAR22_ T cells, ΔTRAC_CAR22_ΔB2M T cells, and ΔTRAC_CAR22_ΔB2M_HLAE_ T cells were co-cultured with RAJI-luc tumor cells (1 × 10^5^) at CAR T to RAJI ratio = 5:1 and 2.5:1 in the presence or absence of in vitro-expanded and activated NK cells at a CAR T cell to NK-cell ratio of 1:1 and 0.5:1 in a total volume of 500 μL of Xvivo-15 media supplemented with 5% AB serum in a 48-well plate. The cell mixture was incubated for 24 h before determining the luminescence of 25 μL of cell suspension using 25 μL of ONE-Glo reagent (Promega). The cell mixture was then spun down, the supernatant was discarded and replaced by 250 μL of fresh complete Xvivo-15 media containing 1 × 10^5^ RAJI-Luc cells, additional 250 μL of media was added to each well with or without NK cells depending on the experimental group. The resulting cell mixture was incubated for 24 h. This protocol was repeated for 4 days. An additional 20 μL from the cell culture was used for flow-cytometry analysis at days 2, 3, and 4. Flow panel included the following anti-human antibodies: CD22EC-mFc (Lakepharma, 1/300), APC-Goat Anti-Mouse IgG (1/100, Jackson, cat#115135164), PE-TCRab (1/100, Miltenyi, cat# 130113539), PECy7-HLA-E (1/100,Biolegend, cat# 342608), VioBlue-HLA-ABC (1/100, Miltenyi, cat# 130120435), PercPCy5.5-CD56 (1/100, Biolegend, cat# 362526), FITC-CD19 (1/50, BDbioscience, cat# 555412). Samples were acquired using the NovoCyte Penteon flow cytometer (Agilent), which enables direct volumetric absolute count without the need for reference counting beads.

### Flow-cytometry analysis

Cells in U-bottom 96-well plate were spun down and washed with PBS (150 μL/well) at 300 × *g* for 2 min. Prior to surface staining, cells were stained with Fixable Viability Dye eFluor 450 or eFluor 780 (eBiosciences) according to the manufacturer’s instructions. The cells were then stained with antibodies diluted in FACS buffer (4% FBS + 5 mM EDTA + 0.05% azide in PBS, 20 μL/well) for at least 15 min in the dark at 4 °C. Cells were washed with PBS (150 μL/well), spun at 300×*g* for 2 min, and resuspended in fix buffer (4% paraformaldehyde in PBS, 100 μL/well). Sample collection was performed on a MacsQuant (Miltenyi), FACSCanto II cytometer (BD) or NovoCyte Penteon flow cytometer (Agilent), and data were analyzed using FlowJo V.10.6.1 (Treestar).

### Mouse models

All procedures involving animals were approved by The Mispro Institutional Animal Care and Use Committee and were performed e in accordance with the guidelines of the PHS (Public Health Service) Policy on Humane Care and Use of Laboratory Animals, OLAW (Office of Laboratory Animal Welfare), and the USDA (United States Department of Agriculture) AWA (Animal Welfare Act). Experimental/control animals were co-housed. The method for euthanasia was CO2 asphyxiation followed by cervical dislocation to assure death. Humane endpoint criteria for tumor models were (i), weight loss greater than or equal to 20% from baseline, (ii), abnormal gait, paralysis, or inability to ambulate properly, (iii), respiratory distress/labored breathing, (iv), lethargy or persistent recumbency, and (v), loss of righting reflex or other abnormal neurological behaviors.

Sixty 8-weeks-old, female NOD.Cg-Prkdcscid Il2rgtm1Wjl/SzJ (NSG) mice and 30 8-weeks-old, female NOD.Cg-Prkdcscid Il2rgtm1Sug Tg(CMV-IL-2/IL-15)1-1Jic/JicTac (hIL-15 NOG) mice were obtained from The Jackson Laboratory (Stock # 005557) and Taconic Biosciences (Stock # 13683-F), respectively. Animals were housed in SPF animal facility. Mouse room light cycles were on a 12 h on/off (on from 6 am to 6 pm and off from 6 pm to 6 am), temperature reading was maintained between 68 and 79 F, and humidity between 30 and 70%.

### In vivo antitumor activity of ΔTRAC_CAR_ΔB2M_HLAE_

To assess the antitumor activity of ΔTRAC_CAR123_ΔB2M_HLAE_ in vivo, 7-week-old NSG mice were adoptively transferred at day 0 with MOLM13-Luc-GFP leukemia cell line (2.5 × 10^5^ cells/mouse in 100 μL of PBS via i.v. injection). On day 7, the mice randomly received a single-dose treatment of mock-transduced T cells, ΔTRAC_CAR_T cells, ΔTRAC_CAR_ΔB2M T cells, ΔTRAC_CAR_ΔB2M_HLAE_ T cells or no T cells; experimental groups received a total of 7 × 10^6^ viable CAR(+) cells/mouse in 100 μL of PBS via intravenous injection. The mice were then monitored for health, weighed at least twice weekly, and followed to measure survival. Disease progression was monitored on a weekly basis by in vivo bioluminescence imaging starting at day 15. Mice were intraperitoneally injected using a XenoLight D-luciferin (PerkinElmer, 200 μL/mouse, 15 mg/mL stock solution) prior to data acquisition. All live imaging was performed on Spectrum-CT apparatus (PerkinElmer) and Living Image software (Caliper). The endpoint was defined as the presence of ≥20% weight loss, hind limb paralysis, labored respiration, or inability to eat/drink. Average radiance was determined on dorsal and ventral settings, averaged, and plotted as a function of time to depict tumor growth.

### In vivo enrichment of ΔTRAC_CAR_ΔB2M_HLAE_ in hIL-15 NOG mice engrafted with human PBMCs

To assess the persistence of ΔTRAC_CAR_ΔB2M_HLAE_ T cells in vivo, cryopreserved human PBMCs acquired from ALLCELLS (cat # PB006F) were thawed as described above. NK-cell depletion in freshly thawed PBMCs was performed by two successive rounds of CD56 positive selection using a NK Cell Isolation kit (Miltenyi Biotec, # 130-092-657) at twice the recommended reagent volumes. Immediately after thawing and NK depletion, cells were adoptively transferred to 7-weeks-old hIL-15 NOG mice (6 × 10^6^ cells/mouse in 100 µl PBS via tail vein injection). At day 12, mice within the two cohorts were randomly injected intravenously with ΔTRAC_CAR_ΔB2M_HLAE_ T cells (2.5 × 10^6^ cells/mouse in 100 µl PBS). On day 16, spleens harvested from humanely euthanized mice were processed to single-cell suspensions. Cells were suspended in 2% FBS/PBS, passed through a 70-µm strainer, and treated with RBC lysis buffer (eBioscience). Residual cells were labeled with monoclonal antibodies in 2% FBS/PBS buffer for 30 min at 4°, washed with 2%FBS/PBS, and subsequently fixed in 4% PFA. Sample collection was performed on FACSCanto II cytometer (BD Biosciences) and data were analyzed using FlowJo v10.6.1 (Treestar).

### Mass cytometry analysis of NK cells from AML, ALL, and healthy donors

PBMCs were processed as previously described with slight modifications^33^. PBMCs were thawed and washed with RPMI 1640 medium supplemented with 10% fetal calf serum (FCS) and incubated in RPMI 1640 with 2% FCS and 1/10000 Pierce^®^ Universal Nuclease 5kU (Thermo Fisher Scientific, Waltham, MA, USA) at 37 °C with 5% CO_2_ for 30 min. Cells were incubated with cisplatin 0.1 M to stain dead cells. Nonspecific epitopes were blocked using 0.5 mg/mL Human Fc Block (BD Biosciences, San Jose, CA, USA). PBMCs were stained for 45 min at 4 °C with a mix of extracellular antibodies (see Supplemental Table 7) and barcoded with the Cell-ID™ 20-Plex Pd *Barcoding Kit* (Fluidigm, San Francisco, CA, USA) according to the manufacturer’s recommendations. Cells were washed with Maxpar® cell staining buffer (CSM) (Fluidigm) and samples were combined and stained with metal-labeled anti-phycoerythrin secondary antibodies for 30 min at 4 °C. After centrifugation, cells were washed with CSM and permeabilized with Foxp3 Staining Buffer (eBioscience, San Diego, CA, USA) for 40 min at 4 °C. Intracellular nonspecific epitopes were blocked using 0.5 mg/mL Human Fc Block for 40 min at 4 °C and then incubated with a mix of intracellular antibodies for 40 min at 4 °C in Foxp3 Staining Buffer (Supplemental Table 7). Cells were then washed and labeled overnight with 125 nM iridium intercalator (Fluidigm) in Cytofix (BD Biosciences). Finally, cells were diluted in EQTM four-element calibration beads (Fluidigm) and analyzed using a mass cytometer (Helios®, Fluidigm).

### Algorithm-based high-dimensional analysis

NK cells were manually defined as CD13-CD33-CD34-CD45 + CD3-CD19-CD56 +  and exported using FlowJo V10.6.2. Consensus files were generated for NKG2C(+) NK cells and NKG2C(−) NK cells from healthy volunteers and AML or ALL patients with a fixed number of NK cells. Data were arcsinh-transformed with a cofactor of 5. NK-cell populations were automatically defined using the optimized parameters for T-distributed stochastic neighbor embedding (opt-SNE) algorithm. The frequency of each NK cell subpopulation (among total lymphocytes as well as lymphocytosis (G/L) was used to determine the absolute counts of NK-cell subsets using the total cell number per volume unit obtained for each patient.

### Functional characterization of the cytolytic activity of AML patients, healthy donor PBMCs toward ΔTRAC_CAR_ΔB2M_HLAE_

Seven AML patients from Institut Paoli Calmettes (Marseille, France) entered this study after informed consent, obtained from all participants in accordance with the Declaration of Helsinki. Peripheral blood was collected at the time of diagnosis and the mononuclear cells (<30% of blasts) were isolated by density gradient centrifugation (Lymphoprep; AbCys) and cryopreserved in RPMI 1640 supplemented with 10% heat-inactivated FCS (Eurobio) containing 10% of DMSO (Sigma-Aldrich). PBMCs from healthy donors (HD) were obtained from blood samples provided by the Etablissement Français du Sang (EFS, Marseille, France), after isolation by density gradient centrifugation (Lymphoprep; AbCys).

In vitro cytotoxicity assays were performed with PBMCs from HD donors or AML patients and engineered CAR T cells according to the following procedure. Cryopreserved anti CD123 CAR T cells (ΔTRAC_CAR123_ΔB2M_HLAE_ or ΔTRAC_CAR123_ΔB2M_HLAE_), engineered according to the process delineated earlier, were thawed and reactivated on CD123-mFC coated plate during 4 days and then cultured in CTS OpTmizer media supplemented with 5% human AB serum and 40 ng/ml IL-2 for 4 additional days before cytotoxicity assay. Reactivated CAR T cells were labeled with Celltrace Violet dye according to the manufacturer’s instructions. PBMCs effectors and labeled CAR T-cell targets (effector:target ratio 10:1) were co-incubated for 24 h at 37 °C in a final volume of 0.2 mL of CTS OpTmizer media supplemented with 5% human AB serum and 40 ng/ml IL-2. Cells were then collected, washed twice, and stained for surface markers (CD45, CD3, CD56, HLA-E), and viability dye (LIVE/DEAD fixable Near-IR). Regarding intracellular stainings, cells were fixed and permeabilized (BD Cytofix/Cytoperm™) according to the manufacturer’s instructions and stained with anti-IFN-γ antibodies. Cells were finally resuspended in PBS and analyzed on a BD FACS LSRII. The antibodies and reagents used for cytometry are listed in Supplementary Table 8.

### Reporting summary

Further information on research design is available in the Nature Research Reporting Summary linked to this article.

## Supplementary information

Supplementary Information

Reporting Summary

## Data Availability

The authors declare that all relevant data are available in the Source Data file provided in the Supplementary Information files. Source data are provided with this paper.

## Source data

Source Data
