# Supplementary material for: Endowing universal CAR T-cell with immune-evasive properties using TALEN-gene editing
Source: Nat Commun. 2022 Jun 30;13:3453. doi: 10.1038/s41467-022-30896-2 (PMC9247096; doi:10.1038/s41467-022-30896-2)
Supplement: Supplementary file 1 — Supplementary Information [file 41467_2022_30896_MOESM1_ESM.pdf]

Supplementary Materials

Endowing Universal CAR T-cell with Immune-Evasive Properties using TALEN-Gene Editing.

Sumin Jo[‡1], Shipra Das[‡1], Alan Williams[‡1], Anne-Sophie Chretien[2], Thomas Pagliardini[2], Aude Le Roy[2], Jorge Postigo Fernandez[1], Diane Le Clerre[3], Billal Jahangiri[1], Isabelle Chion-Sotinel[3], Sandra Rozlan[3], Emilie Dessez[3], Agnes Gouble[3], Mathilde Dusséaux[3], Roman Galetto[3], Aymeric Duclert[3], Emanuela Marcenaro[4], Raynier Devillier[2], Daniel Olive[*2], Philippe Duchateau[3], Laurent Poirot[1,3], Julien Valton[*1]

[1] Cellectis, Inc., 430 East 29th Street, New York, NY 10016, USA

[2] Institut Paoli Calmettes, 27 Bd Leï Roure, CS 30059, 13273 Marseille Cedex 09, France

[3] Cellectis, 8 rue de la Croix Jarry, 75013 Paris, France

[4] CEBR, University of Genova, Viale Benedetto XV, 9 piano -1 della Palazzina di Clinica Oculistica 16132 Genova, Italy

[‡] Co-first Authors

[*] Corresponding authors: daniel.olive@inserm.fr and julien.valton@cellectis.com

Inventory of supporting information

-Supplementary figure 1. Specificity of TALEN-mediated TRAC and B2M double knock out and CAR_m/$HLAE_m$ targeted insertion.

-Supplementary figure 2. Depletion of HLA-ABC from the surface of T-cell prevents their depletion by alloresponsive T-cells.

-Supplementary figure 3. Targeted insertion of HLA-E at the B2M locus efficiently prevents NK cell-mediated depletion of $\Delta TRAC_{CAR}\Delta B2M_{HLAE}$ cells *in vitro*.

-Supplementary figure 4. Investigation of NK cells activation/degranulation in the presence of engineered CAR T-cell $\Delta TRAC_{CAR123}\Delta B2M$ and $\Delta TRAC_{CAR123}\Delta B2M_{HLAE}$.

-Supplementary figure 5. Targeted insertion of HLA-E at the B2M locus enables efficient and prolonged antitumor activity of $\Delta TRAC_{CAR}\Delta B2M_{HLAE}$ in the presence of activated NK cells *in vitro*.

-Supplementary figure 6. Absolute counts of NK cells in AML and ALL patients undergoing standard induction chemotherapy.

-Supplementary figure 7. Targeted insertion of HLA-E at the B2M locus efficiently prevents NK cell-mediated depletion of $\Delta TRAC_{CAR}\Delta B2M_{HLAE}$ cells *in vivo*.

-Supplementary Table 1.

-Supplementary Table 2.

-Supplementary Table 3.

-Supplementary Table 4.

-Supplementary Table 5.

-Supplementary Table 6.

-Supplementary Table 7.

-Supplementary Table 8.

Supplemental figures

a

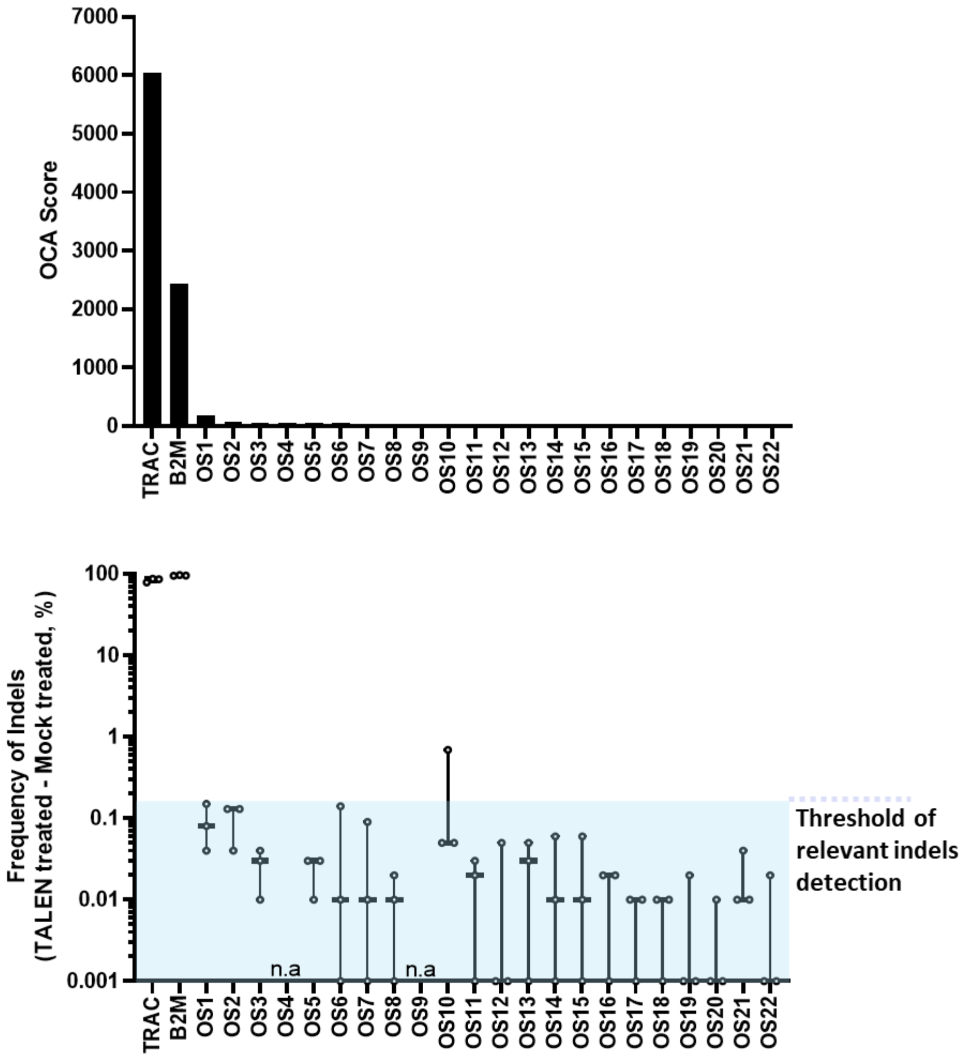

b

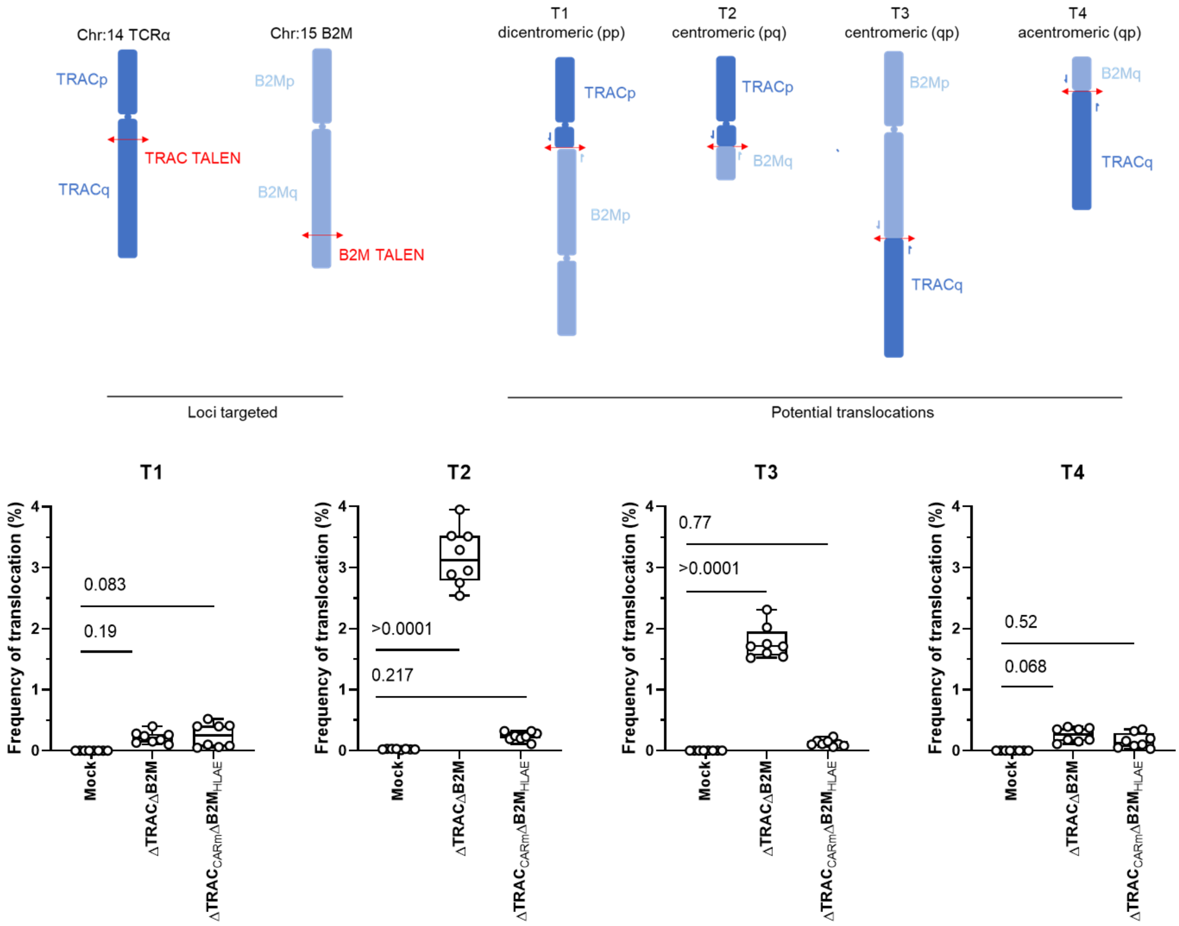

Chr:14 TCRα
Chr:15 B2M
TRACp
TRACq
TRAC TALEN
B2Mp
B2Mq
B2M TALEN
T1 dicentromeric (pp)
T2 centromeric (pq)
T3 centromeric (qp)
T4 acentromeric (qp)
Loci targeted
Potential translocations
T1
T2
T3
T4
Frequency of translocation (%)
0.083
0.19
>0.0001
0.217
0.77
>0.0001
0.52
0.068
Mock
ΔTRACΔB2M
ΔTRAC$_{CARm}$ΔB2M$_{HLAE}$

c

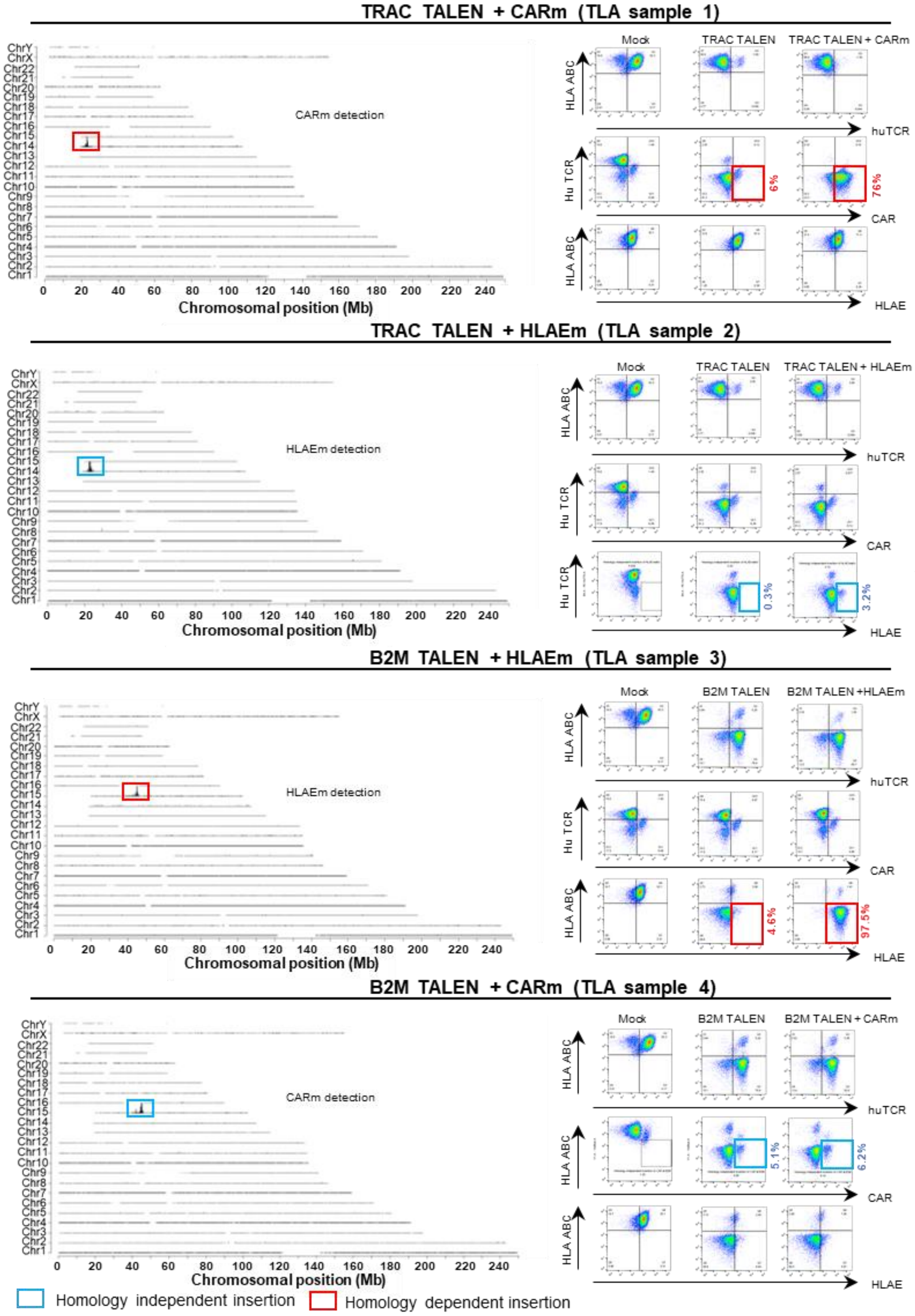
TRAC TALEN + CARm (TLA sample 1)
CARm detection
Chromosomal position (Mb)
Mock
TRAC TALEN
TRAC TALEN + CARm
HLA ABC
huTCR
Hu TCR
CAR
6%
76%
HLAE
TRAC TALEN + HLAEm (TLA sample 2)
HLAEm detection
TRAC TALEN + HLAEm
0.3%
3.2%
B2M TALEN + HLAEm (TLA sample 3)
B2M TALEN
B2M TALEN +HLAEm
4.6%
97.5%
B2M TALEN + CARm (TLA sample 4)
B2M TALEN + CARm
5.1%
6.2%
Homology independent insertion
Homology dependent insertion

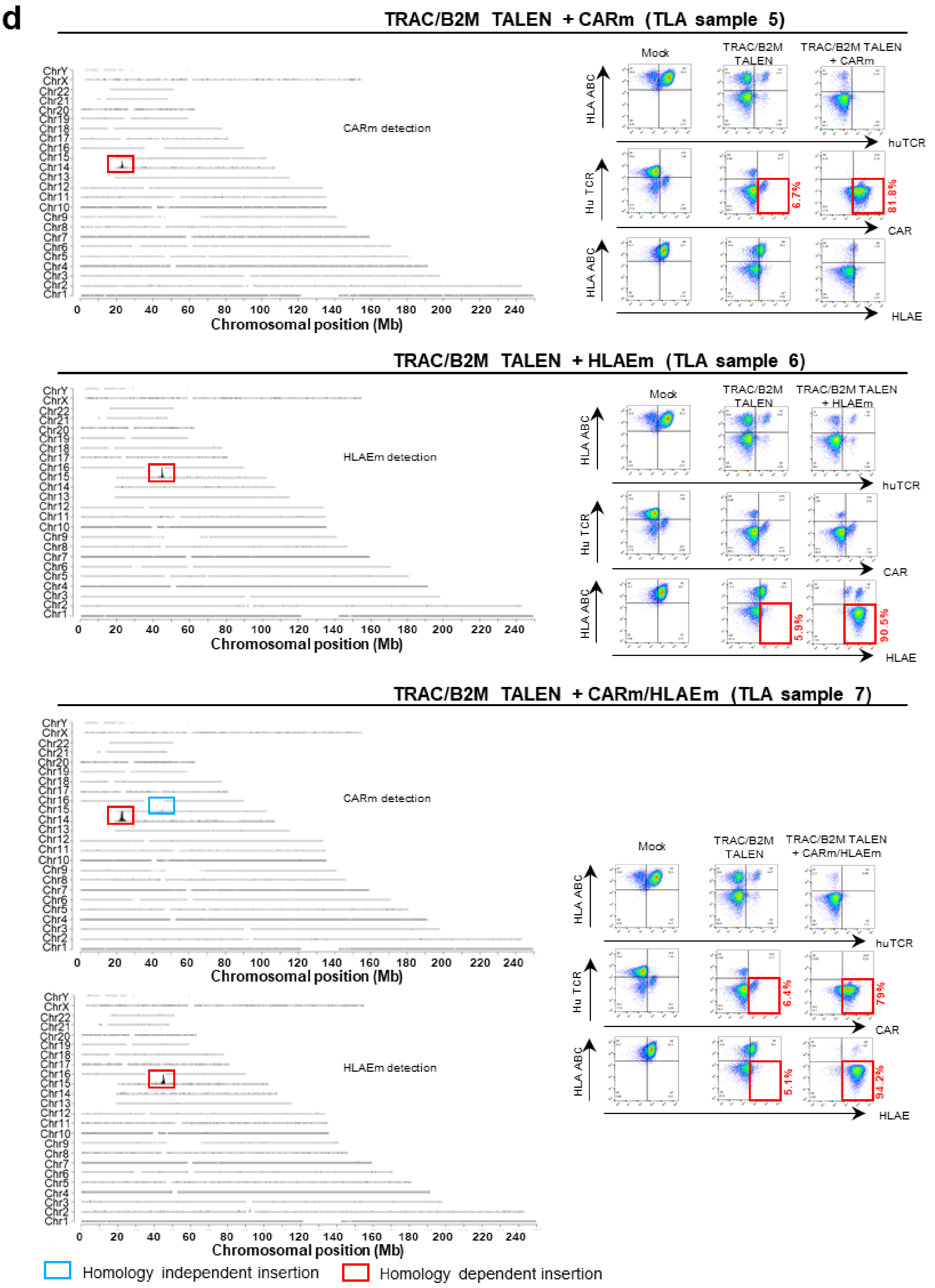
d
TRAC/B2M TALEN + CARm (TLA sample 5)
CARm detection
Chromosomal position (Mb)
Mock
TRAC/B2M TALEN
TRAC/B2M TALEN + CARm
HLA ABC
huTCR
Hu TCR
CAR
HLAE
6.7%
81.8%
TRAC/B2M TALEN + HLAEm (TLA sample 6)
HLAEm detection
TRAC/B2M TALEN + HLAEm
5.9%
90.5%
TRAC/B2M TALEN + CARm/HLAEm (TLA sample 7)
TRAC/B2M TALEN + CARm/HLAEm
6.4%
79%
5.1%
94.2%
Homology independent insertion
Homology dependent insertion

e

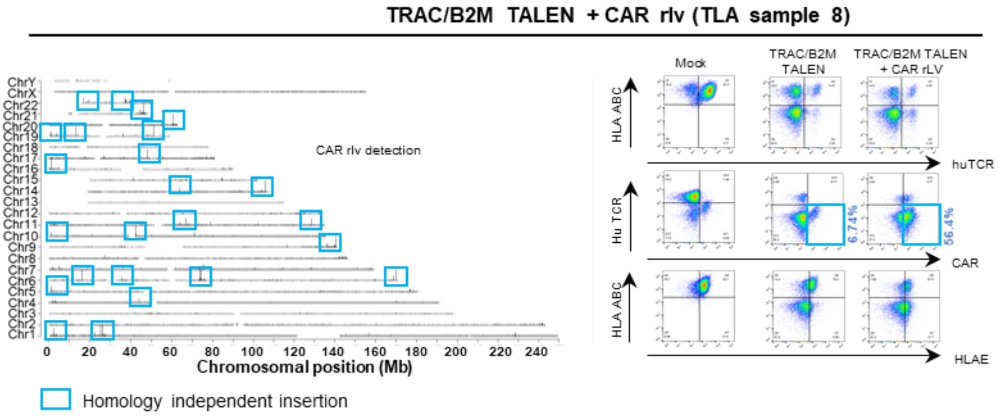

f

	Experiental layout			
**Sample name**	**TALEN Modifications**		**AAV6 matrix insertion**	
**Sample 1**	TRAC KO	-	CARm123	-
**Sample 2**	TRAC KO	-	-	HLAEm
**Sample 3**	-	B2M KO	-	HLAEm
**Sample 4**	-	B2M KO	CARm123	-
**Sample 5**	TRAC KO	B2M KO	CARm123	-
**Sample 6**	TRAC KO	B2M KO	-	HLAEm
**Sample 7**	TRAC KO	B2M KO	CARm123	HLAEm
**Sample 8**	TRAC KO	B2M KO	rlv CAR-2A-HLA-E	-

Supplementary figure 1. Specificity of TALEN-mediated TRAC and B2M double knock out and CAR_m/$HLAE_m$ targeted insertion. **a** Top panel, Oligo Capture Assay (OCA) identifying on-site and candidate off-site TALEN activity in T-cells (n=2 donors) co-transfected with mRNA encoding TRAC and B2M TALEN. OCA score obtained for the two onsites (TRAC and B2M) as well as the 22 first candidate off-sites, are indicated. **a** Bottom panel, frequencies of indels obtained by high-throughput DNA sequencing of TRAC and B2M TALEN on-sites and of the 22 first candidate off-sites identified by OCA, except for OS4 and OS9, that could not be PCR-amplified (n.a). Frequencies of insertion and deletions (indels) obtained in TALEN treated T-cells (n=3 donors) subtracted from those obtained in mock treated T-cells (n=3 donors) are indicated. Each point represents the frequency of indels obtained in one independent experiment performed with a given T-cell donor and the horizontal bar represents the median frequency of indels. The blue frame indicates the data points falling below the threshold (T) of significant indels detection (T=0.16%). **b**, qPCR analysis of canonical T1, T2, T3 and T4 translocation expected to occur between the TRAC and B2M loci targeted by their respective TALEN. **b top panel**, schemes illustrating the TRAC and B2M loci on chromosomes 14 and 15,

respectively and the potential translocation generated after co-treatment of T-cells with the TRAC and B2M TALEN . **b bottom panel**, frequencies of translocation events detected by qPCR are shown for $\Delta TRAC_{CAR123}\Delta B2M_{HLAE}$ and relevant controls T-cells (negative control, mock treated T-cell and positive control, TRAC and B2M TALEN treated T-cell, ΔTRACΔB2M) engineered out of 2 donors (n=4 technical replicates). In each box plot, the central mark indicates the median, the bottom and top edges of the box indicate the interquartile range (IQR), and the whiskers represent the maximum and minimum data point. Two-way ANOVA was used to compute statistics, and p values are indicated. **c**, **d, e left panels**, Targeted locus amplification (TLA) results. **c**, TLA results obtained from T-cells transfected with either TRAC or B2M TALEN and transduced with CAR_m or $HLAE_m$ AAV6 particles. **d**, TLA results obtained from T-cells co-transfected with TRAC and B2M TALEN and transduced with CAR_m or $HLAE_m$ AAV6 or co-transduced with CAR_m and $HLAE_m$ AVV6 particles. **e**, TLA results obtained from T-cells co-transfected with TRAC and B2M TALEN and transduced with rlv particles encoding a CAR123-2A-HLAE construct. Homology dependent- (specific targeted integration) and homology independent- (non-specific integration) integration of AAV6 matrices are indicated in the red and blue boxes, respectively. **c**, **d, e right panels**, Flow cytometry analysis illustrating the frequency of TCR, HLA ABC, CAR and HLA-E expression within samples 1-8 analyzed by TLA and those used as relevant controls. Specific targeted expression and non-specific expression of CAR or HLA-E are indicated in the flow plots as red and blue boxes, respectively. **f,** Experimental layout to generate engineered CAR T-cells analyzed by TLA. Source data are provided as a Source Data file.

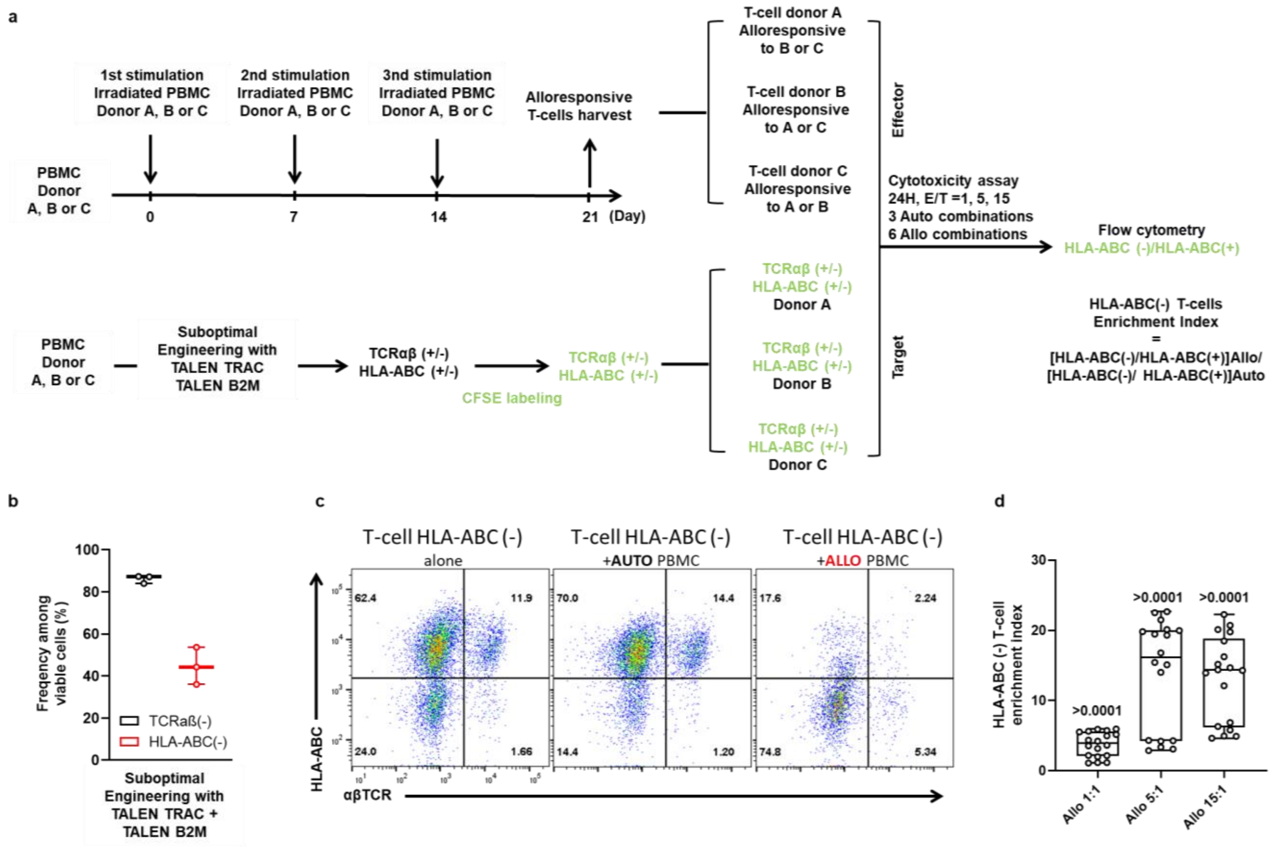

Supplementary figure 2. Depletion of HLA-ABC from the surface of T-cell prevents their depletion by alloresponsive T-cells. a Schematic illustrating the generation of alloresponsive T-cells and subsequent Mix lymphocyte reaction (MLR) performed with TCRαβ/B2M double knock out T-cells. PBMC from donors A, B and C were thawed and mixed at a 1:1 ratio with irradiated PBMC from donor A, B or C at day 0. Cells were co-cultivated for 7 days before being re-stimulated with irradiated PBMC from donor A, B or C at day 7. The same procedure was reiterated one more time before harvesting alloresponsive T-cells at day 21. In parallel, TCRαβ/B2M double knock out T-cells were generated from donor A, B and C (target), labeled with CFSE dye and eventually mixed with alloresponsive or autologous T-cells (effector) at a target to effector ratio of 1:1, 1:5 and 1:15. Cells were recovered after 24 hours of co-culture and analyzed by flow cytometry to determine the frequency of CFSE(+) HLA-ABC(+) and CFSE(+) HLA-ABC(-) T-cell targets remaining. An HLA-ABC(-) enrichment index was determined to quantify the resistance of CFSE(+) HLA-ABC(-) engineered T-cell to alloresponsive T-cells. **b** Efficiency of TALEN-mediated depletion of TCRαβ and HLA-ABC surface expression (n=3 T cell donors). Each point represents the frequency of TCRαβ and HLA-ABC knock-out obtained in a

given donor. **c** representative flow cytometry plots showing the evolution of TCRαβ(+/-)HLA-ABC(+/-) T-cell targets when co-cultured in the presence of autologous PBMC (Auto PBMC) or in the presence of allogeneic PBMC (Allo PBMC) for 24 hours. **d** Box plots representing the HLA-ABC (-) enrichment index obtained when engineered TCRαβ(+/-)HLA-ABC(+/-) T-cell targets were co-cultured with autologous and alloresponsive T-cells at different ratios (3 technical replicates, with 3 donors for T-cell effector and 3 donors for engineered T-cell targets). *p*-value obtained from an unpaired t-test performed to assess the statistical significance of the HLA-ABC(-) enrichment index, are documented on the top of each box plot. On each box plot, the central mark indicates the median, the bottom and top edges of the box indicate the interquartile range (IQR) and the whiskers represent the maximum and minimum data point. Source data are provided as a Source Data file.

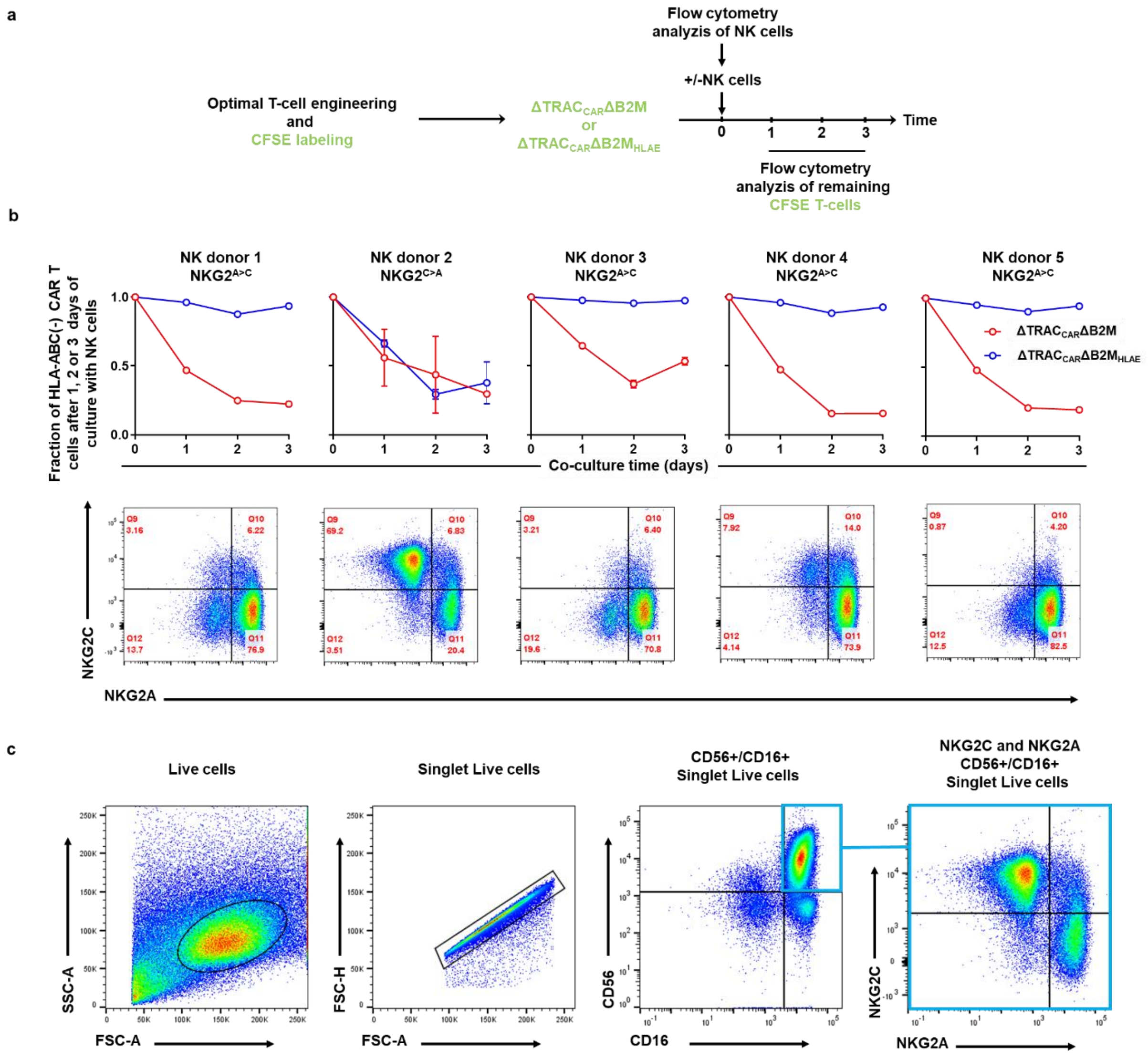

Supplementary figure 3. Targeted insertion of HLA-E at the B2M locus efficiently prevents NK cell-mediated depletion of ΔTRAC$_{CAR}$ΔB2M$_{HLAE}$ cells *in vitro*. a Schematic of the experimental design to investigate the susceptibility of ΔTRAC$_{CAR}$ΔB2M$_{HLAE}$ cells, engineered with optimal conditions, to NK cell-dependent depletion *in vitro*. CFSE labelled-target cells, engineered to obtain a majority of HLA-ABC (-) or HLA-ABC (-) HLA-E(+) subpopulations, were cultured for 3 days in the presence or absence of NK cells coming from 5 different donors. **b top panel**, Plots showing the evolution of the fraction of HLA-ABC(-) cells of ΔTRAC$_{CAR22}$ΔB2M and ΔTRAC$_{CAR22}$ΔB2M$_{HLAE}$ T-cells from 0, 1, 2 or 3 days of co-culture (n=5 NK donors, n=1 T cell donor with 2 technical duplicates). Data are presented as mean values +/- SD. **b bottom panel**, Flow cytometry plots showing the frequency of the NKG2A(+), NKG2C(+) subpopulation among viable CD56(+), CD16(+) double-positive NK cells used to perform the

NK cytotoxicity kinetics illustrated in the top panel (NK from healthy donors, n = 5). **c** Gating strategy used to obtain the flow cytometry plot of **3b**. Source data are provided as a Source Data file.

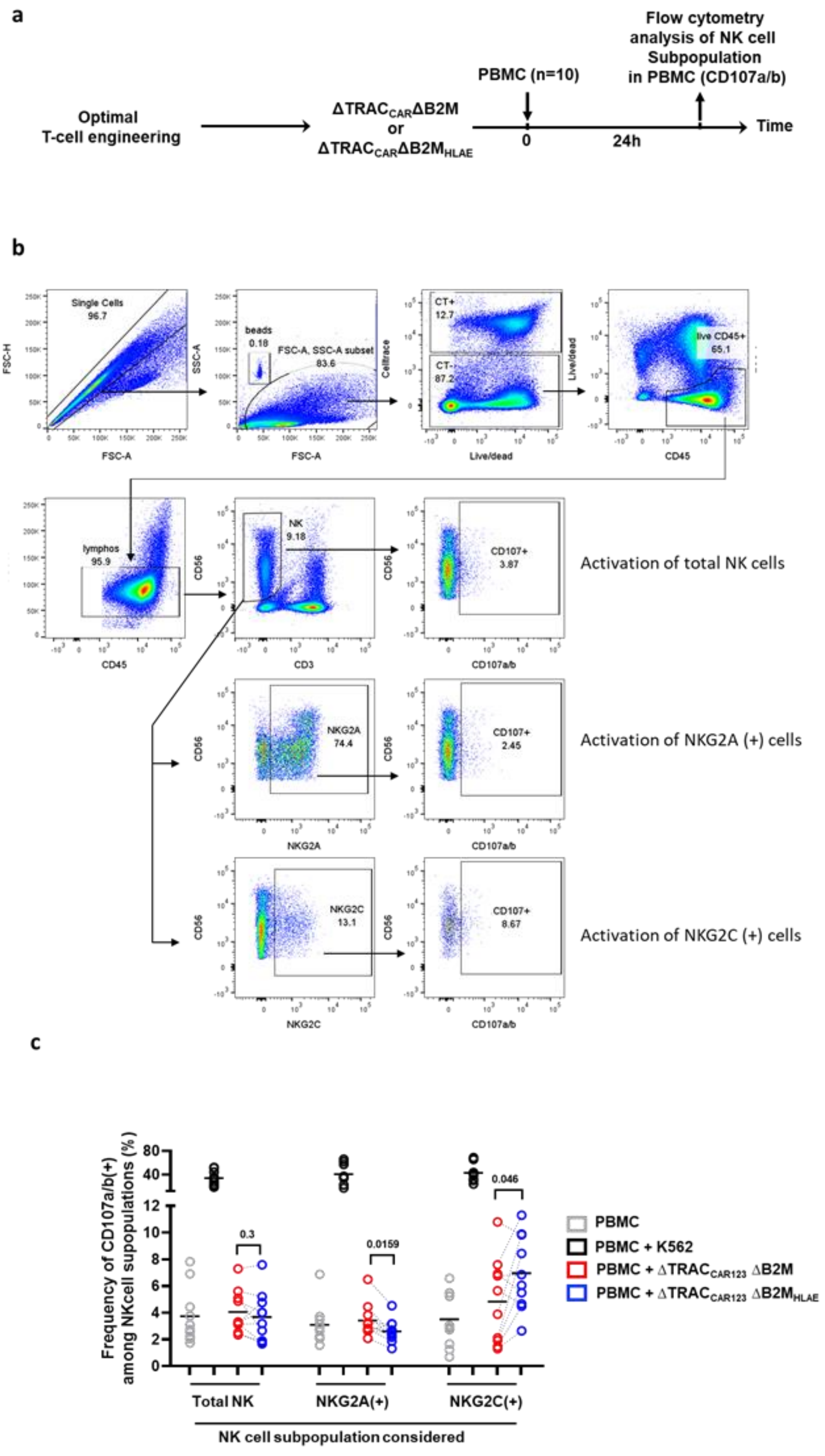
a
Optimal
T-cell engineering
ΔTRACCARΔB2M
or
ΔTRACCARΔB2MHLAE
PBMC (n=10)
0
24h
Flow cytometry
analysis of NK cell
Subpopulation
in PBMC (CD107a/b)
Time
b
Single Cells
96.7
FSC-H
FSC-A
beads
0.18
FSC-A, SSC-A subset
83.6
SSC-A
CT+
12.7
CT-
87.2
Celltrace
Live/dead
live CD45+
65.1
CD45
lymphos
95.9
NK
9.18
CD56
CD3
CD107+
3.87
CD107a/b
Activation of total NK cells
NKG2A
74.4
CD107+
2.45
Activation of NKG2A (+) cells
NKG2C
13.1
CD107+
8.67
Activation of NKG2C (+) cells
c
Frequency of CD107a/b(+)
among NKcell supopulations (%)
0.3
0.0159
0.046
Total NK
NKG2A(+)
NKG2C(+)
NK cell subpopulation considered
PBMC
PBMC + K562
PBMC + ΔTRACCAR123 ΔB2M
PBMC + ΔTRACCAR123 ΔB2MHLAE

Supplementary figure 4. **Investigation of NK cells activation/degranulation in the presence of engineered CAR T-cell ΔTRAC$_{CAR123}$ΔB2M and ΔTRAC$_{CAR123}$ΔB2M$_{HLAE}$.** 0.15^{E}6 ΔTRAC$_{CAR123}$ΔB2M or ΔTRAC$_{CAR123}$ΔB2M$_{HLAE}$ were mixed with 1.5^{E}6 PBMCs sourced from 10 different healthy donors and co-cultured for 24 hours. Cell mixture was then analyzed by flow cytometry to determine the level of activation of NK cell, NKG2A(+) and NKG2C(+) NK cell subpopulations. K562 is used as a positive control of NK activation. **a**, Scheme representing the experimental protocol. **b**, Flow cytometry gating strategy used to decipher NK cell, NKG2A(+) and NKG2C(+) NK cell populations and determine their level of CD107a/b degranulation. **c**, Summary plot illustrating the frequency of activation of NK cell, NKG2A(+) and NKG2C(+) NK cell subpopulations (CD107a/b degranulation) obtained after co-culture with K562, ΔTRAC$_{CAR123}$ΔB2M or with ΔTRAC$_{CAR123}$ΔB2M$_{HLAE}$. Each point represents one experiment performed with a given PBMC batch obtained from a healthy donor (n=10) and the black bar indicate the mean of CD107a/b frequency obtained for each experimental group. Dotted lines indicate results obtained with the same PBMC donor and ΔTRAC$_{CAR123}$ΔB2M or ΔTRAC$_{CAR123}$ΔB2M$_{HLAE}$. The *p*-value obtained from a paired t-test is indicated on the figure.

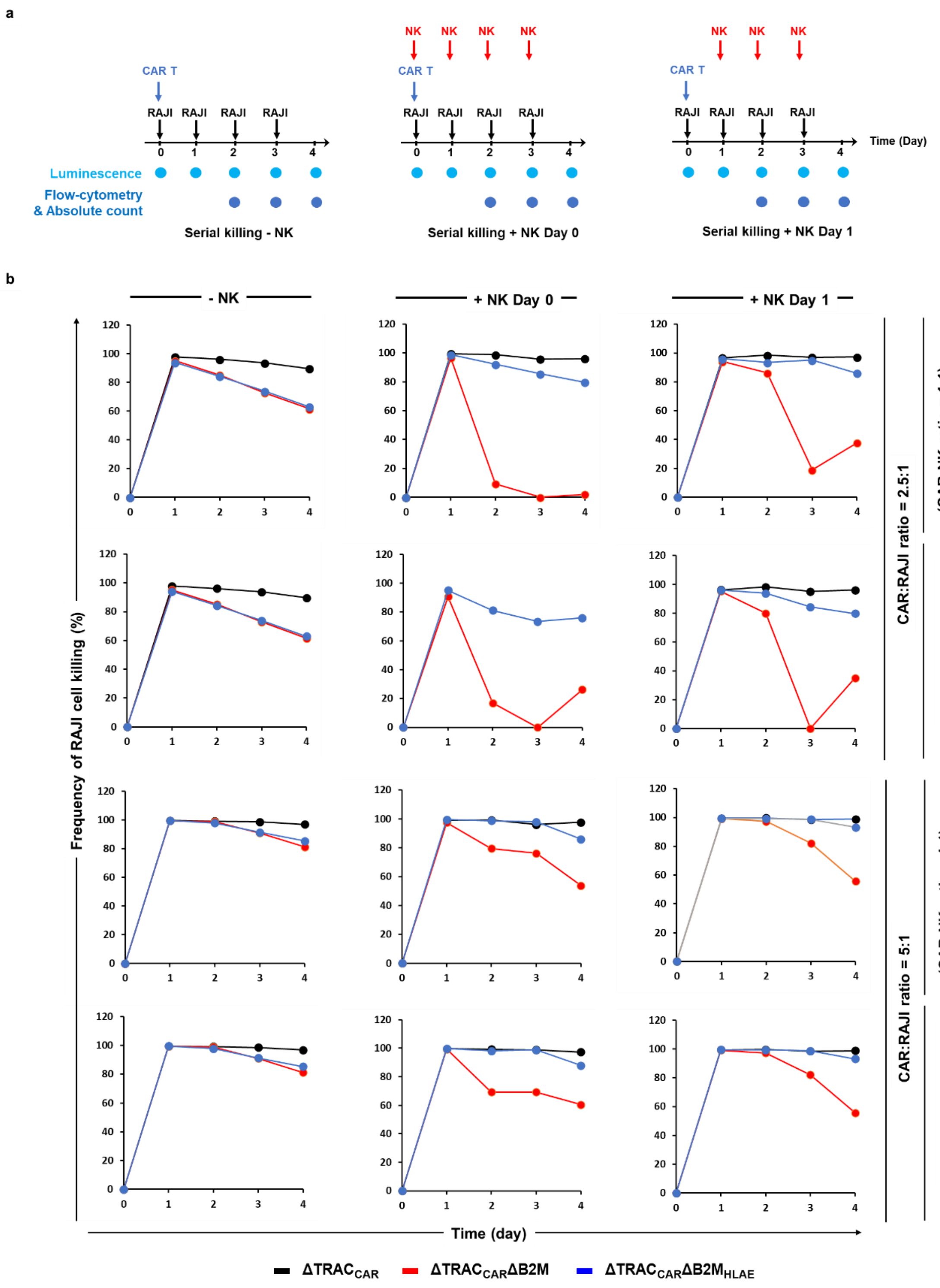
a
CAR T
NK
RAJI
Time (Day)
Luminescence
Flow-cytometry
& Absolute count
Serial killing - NK
Serial killing + NK Day 0
Serial killing + NK Day 1
b
- NK
+ NK Day 0
+ NK Day 1
Frequency of RAJI cell killing (%)
Time (day)
CAR:RAJI ratio = 2.5:1
CAR:RAJI ratio = 5:1
(CAR:NK ratio = 1:1)
(CAR:NK ratio = 1:2)
ΔTRAC$_{CAR}$
ΔTRAC$_{CAR}$ΔB2M
ΔTRAC$_{CAR}$ΔB2M$_{HLAE}$

c

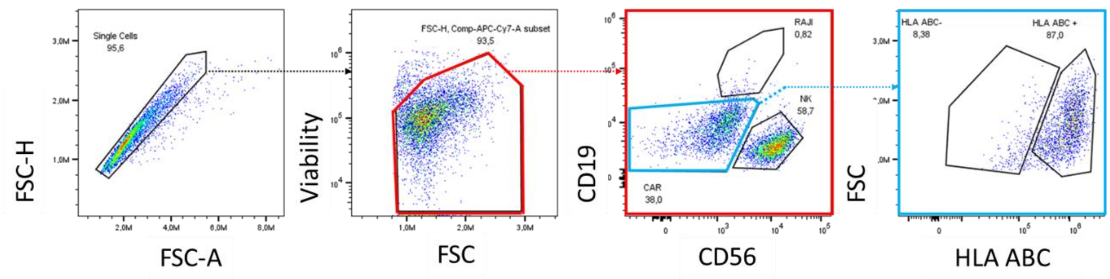
Single Cells
95,6
FSC-H
FSC-A
FSC-H, Comp-APC-Cy7-A subset
93,5
Viability
FSC
RAJI
0,82
NK
58,7
CAR
38,0
CD19
CD56
HLA ABC-
8,38
HLA ABC +
87,0
FSC
HLA ABC

d

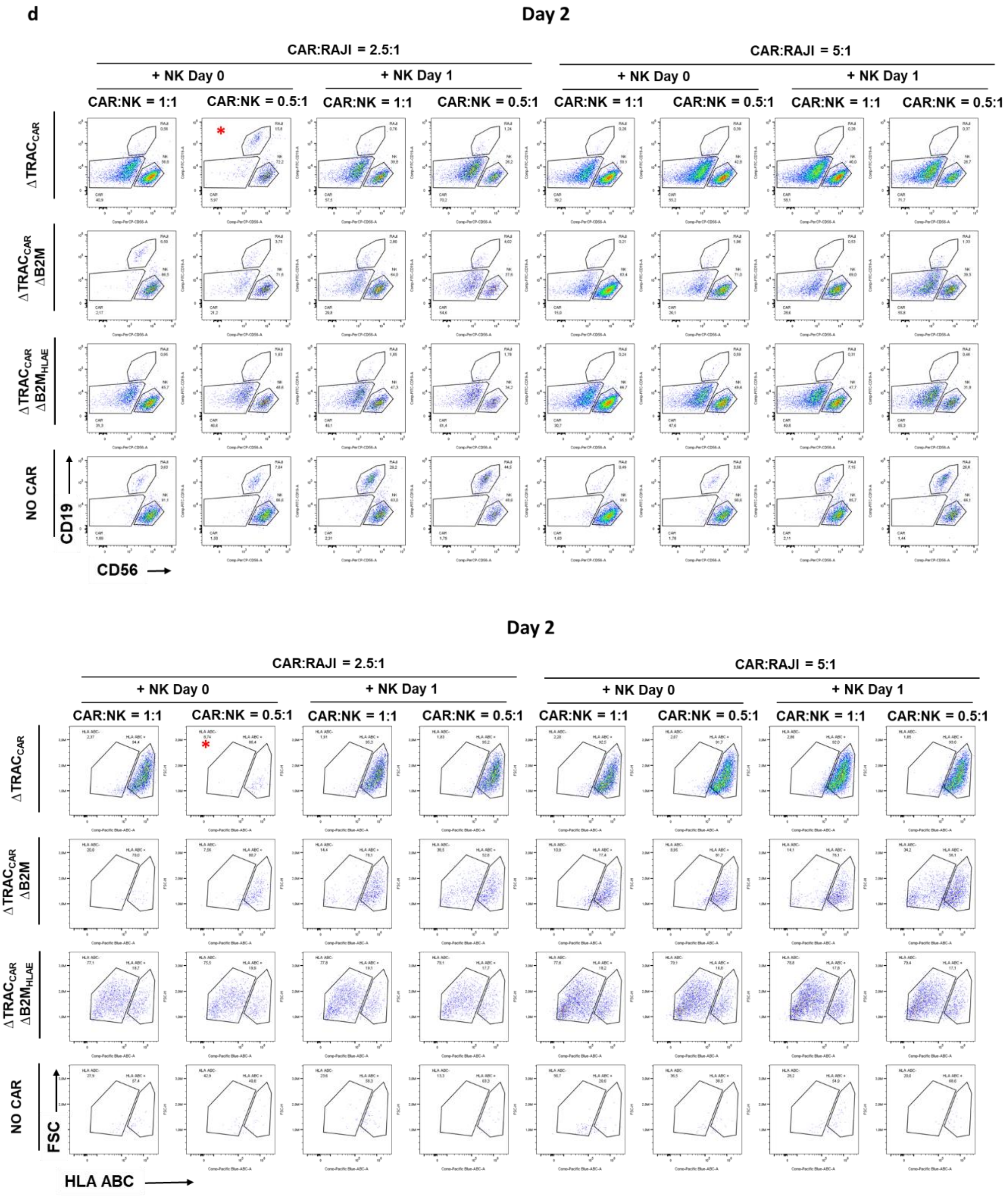
Day 2
CAR:RAJI = 2.5:1
CAR:RAJI = 5:1
+ NK Day 0
+ NK Day 1
CAR:NK = 1:1
CAR:NK = 0.5:1
ΔTRACCAR
ΔTRACCAR ΔB2M
ΔTRACCAR ΔB2MHLAE
NO CAR
CD19
CD56
Day 2
CAR:RAJI = 2.5:1
CAR:RAJI = 5:1
+ NK Day 0
+ NK Day 1
CAR:NK = 1:1
CAR:NK = 0.5:1
ΔTRACCAR
ΔTRACCAR ΔB2M
ΔTRACCAR ΔB2MHLAE
NO CAR
FSC
HLA ABC

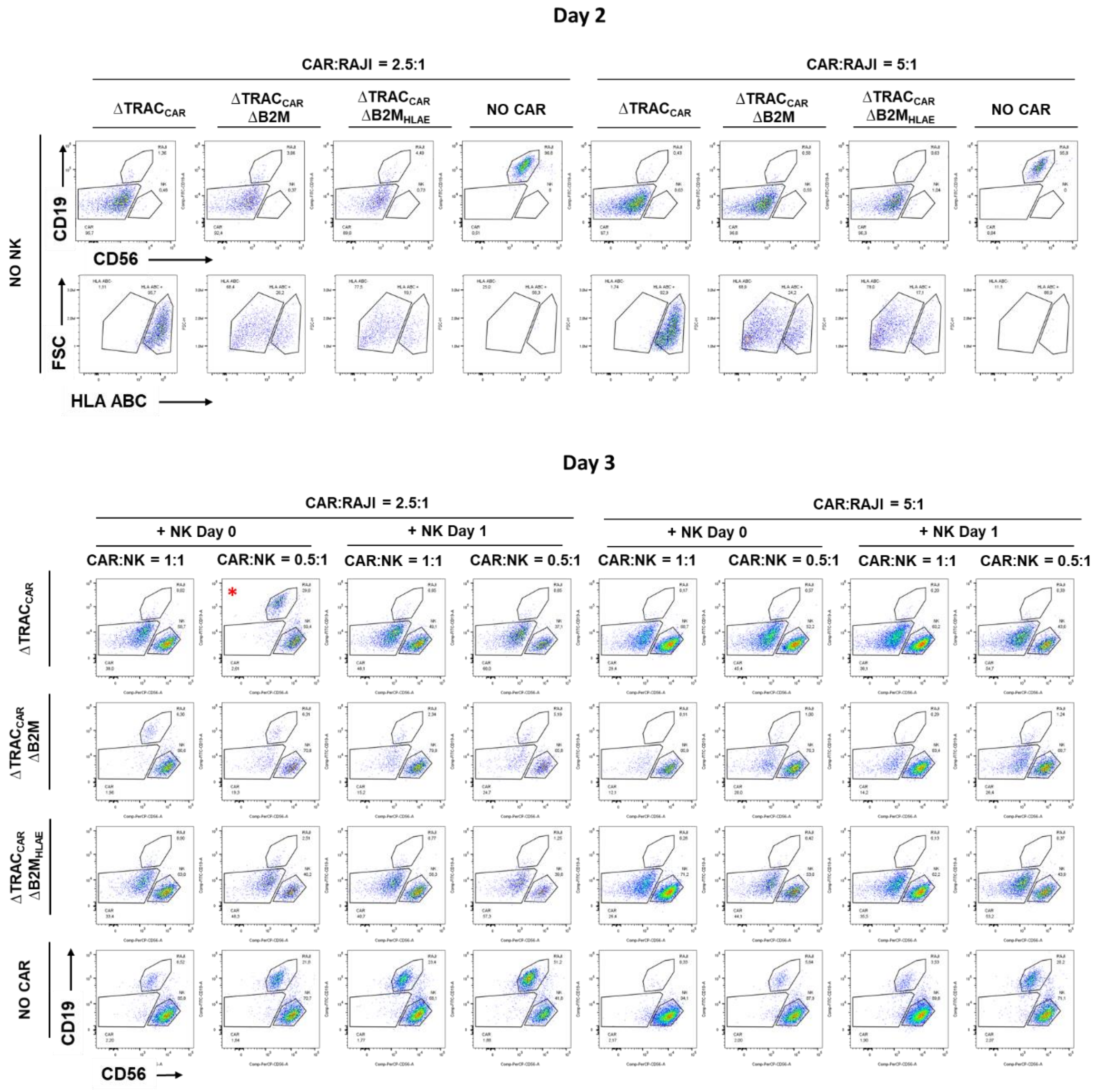
Day 2
CAR:RAJI = 2.5:1
CAR:RAJI = 5:1
ΔTRAC$_{CAR}$
ΔTRAC$_{CAR}$ ΔB2M
ΔTRAC$_{CAR}$ ΔB2M$_{HLAE}$
NO CAR
NO NK
CD19
CD56
FSC
HLA ABC
Day 3
+ NK Day 0
+ NK Day 1
CAR:NK = 1:1
CAR:NK = 0.5:1
*

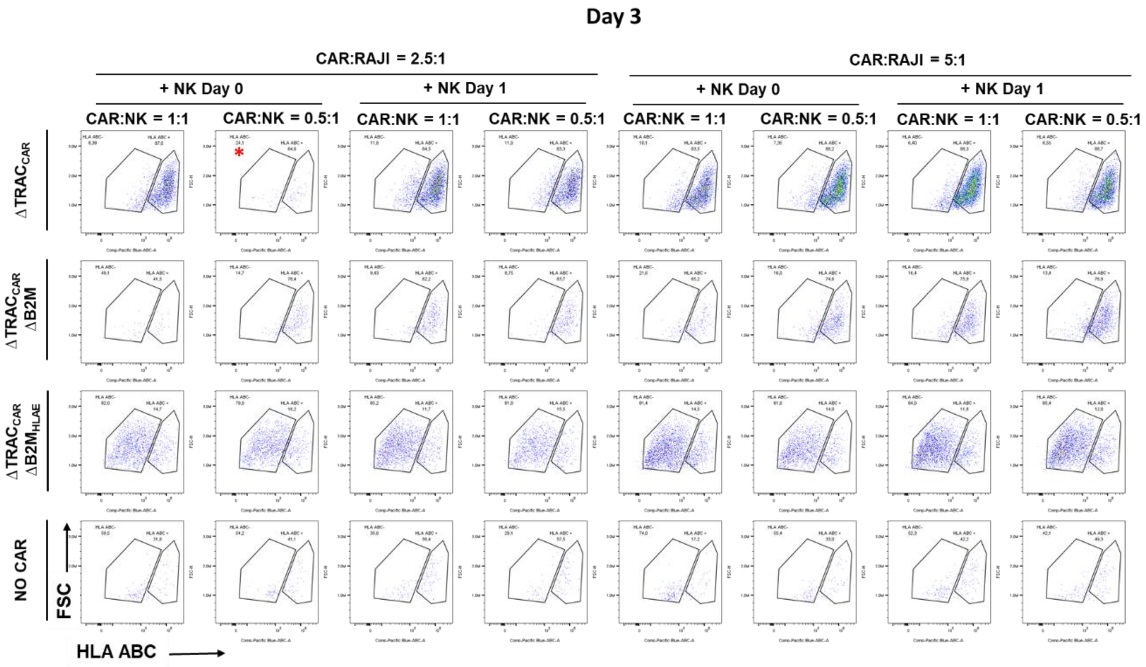
Day 3
CAR:RAJI = 2.5:1
CAR:RAJI = 5:1
+ NK Day 0
+ NK Day 1
CAR:NK = 1:1
CAR:NK = 0.5:1
$\Delta TRAC_{CAR}$
$\Delta TRAC_{CAR}$ $\Delta B2M$
$\Delta TRAC_{CAR}$ $\Delta B2M_{HLAE}$
NO CAR
FSC
HLA ABC

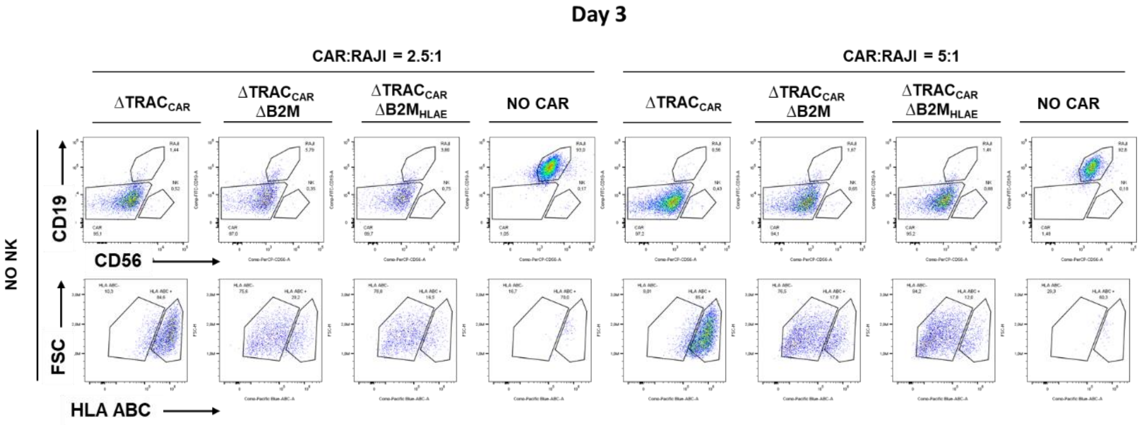
Day 3
CAR:RAJI = 2.5:1
CAR:RAJI = 5:1
$\Delta TRAC_{CAR}$
$\Delta TRAC_{CAR}$ $\Delta B2M$
$\Delta TRAC_{CAR}$ $\Delta B2M_{HLAE}$
NO CAR
NO NK
CD19
CD56
FSC
HLA ABC

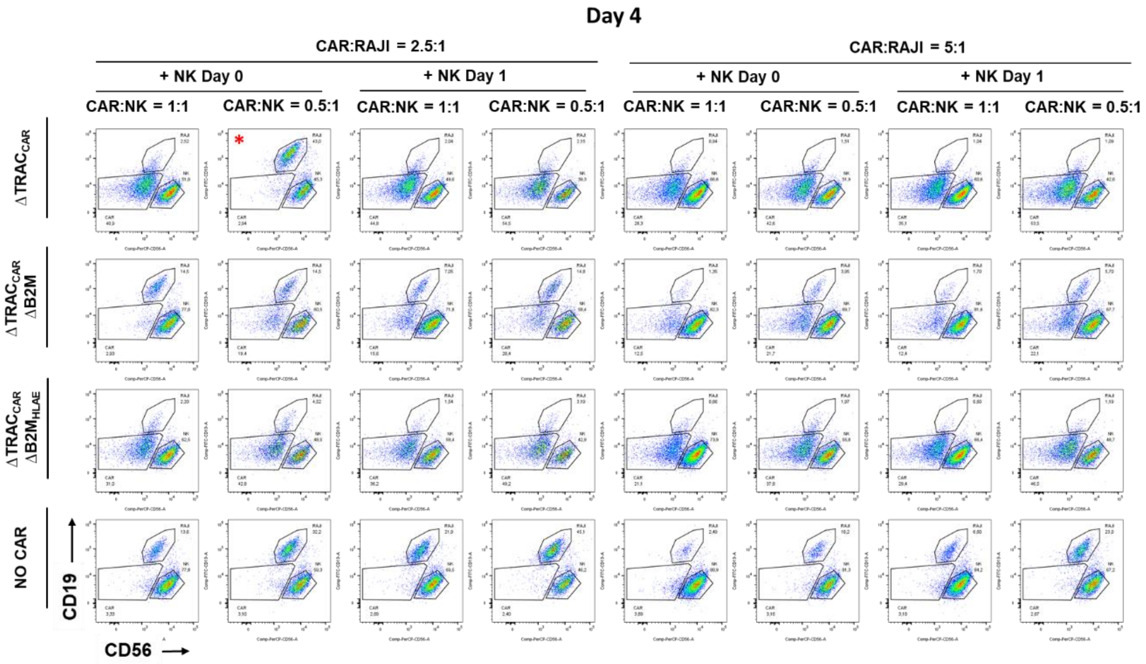
Day 4
CAR:RAJI = 2.5:1
CAR:RAJI = 5:1
+ NK Day 0
+ NK Day 1
CAR:NK = 1:1
CAR:NK = 0.5:1
$\Delta TRAC_{CAR}$
$\Delta TRAC_{CAR}$ $\Delta B2M$
$\Delta TRAC_{CAR}$ $\Delta B2M_{HLAE}$
NO CAR
CD19
CD56

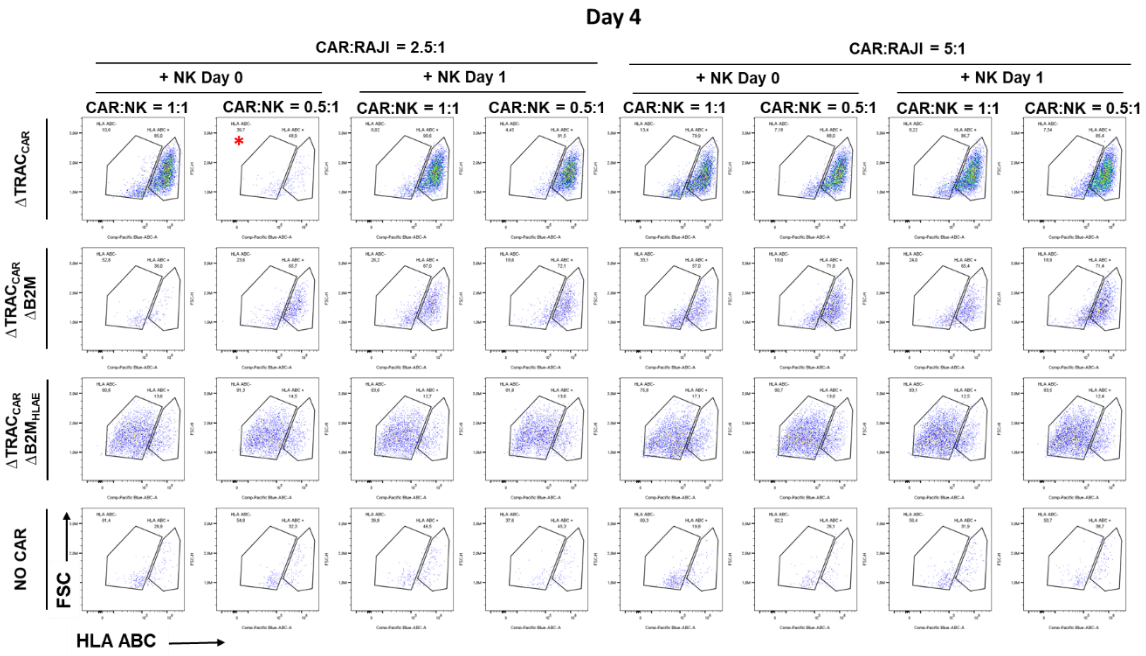
Day 4
CAR:RAJI = 2.5:1
CAR:RAJI = 5:1
+ NK Day 0
+ NK Day 1
CAR:NK = 1:1
CAR:NK = 0.5:1
ΔTRAC CAR
ΔTRAC CAR ΔB2M
ΔTRAC CAR ΔB2M HLAE
NO CAR
FSC
HLA ABC

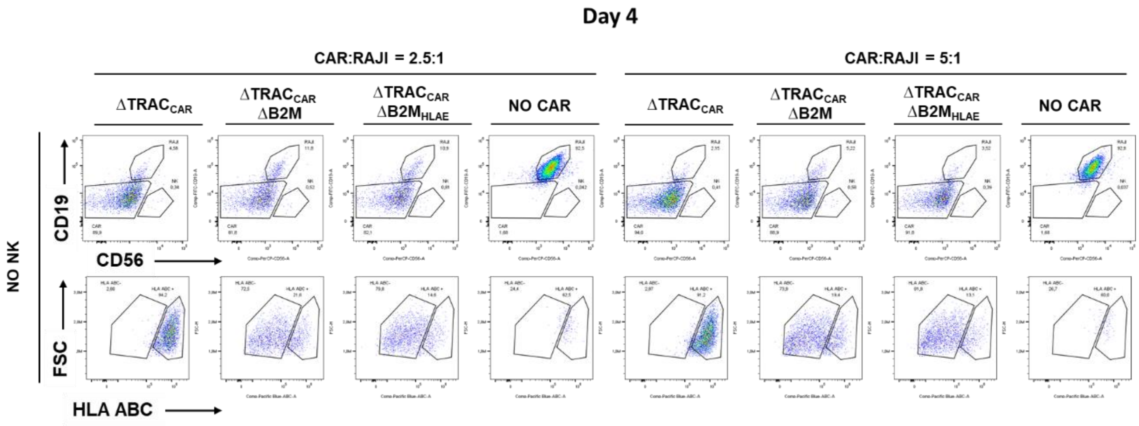
Day 4
CAR:RAJI = 2.5:1
CAR:RAJI = 5:1
ΔTRAC CAR
ΔTRAC CAR ΔB2M
ΔTRAC CAR ΔB2M HLAE
NO CAR
NO NK
CD19
CD56
FSC
HLA ABC

e

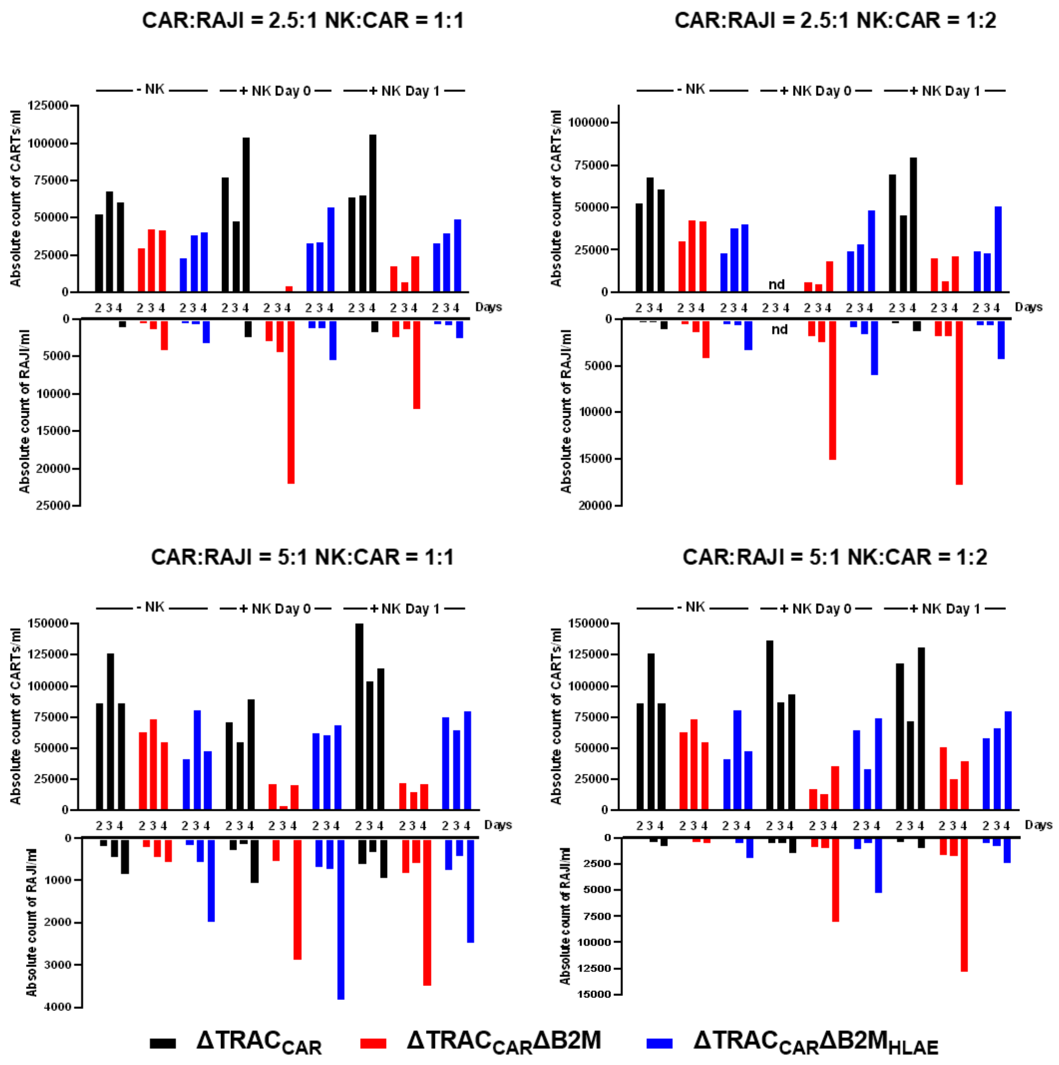

Supplementary figure 5. Targeted insertion of HLA-E at the B2M locus enables efficient and prolonged antitumor activity of $\Delta TRAC_{CAR}\Delta B2M_{HLAE}$ in the presence of activated NK cells *in vitro*. a Schematic showing the serial killing assay designed to investigate the long term antitumor activity of $\Delta TRAC_{CAR22}\Delta B2M_{HLAE}$ cells toward RAJI-Luc cells in the presence or absence of activated NK cells. The three different serial killing assay scenarios investigated are illustrated (No NK, NK Day 0 and NK Day 1). Black, blue and red arrows indicate addition of RAJI cells, CAR T-cells ($\Delta TRAC_{CAR22}$, $\Delta TRAC_{CAR22}\Delta B2M$ and $\Delta TRAC_{CAR22}\Delta B2M_{HLAE}$) and NK cells respectively. Luminescence and flow cytometry analysis of cell populations are indicated at the different measurment time points. **b** Frequency of RAJI-Luc cells killing observed by luminescence with the three different serial killing scenarios performed with a CAR T-cell to RAJI ratio of 2.5:1 and 5:1, in the absence or in the presence of NK cells with a NK to CAR T-

cell ratio of 1:1 and 0.5:1. Each point represents one experiment performed with a given T-cell donor. **c** flow cytometry gating strategy used to analyze cell mixtures at day 2, 3 and 4. **d** flow cytometry analysis of cell mixture obtained at day 2, 3 and 4 for all the experimental conditions described in **a**. **e** Absolute CAR T-cells and RAJI cells counts obtained by flow cytometry analysis (using the gating strategy described in **c**) for the experimental conditions described in **a**. Source data are provided as a Source Data file.

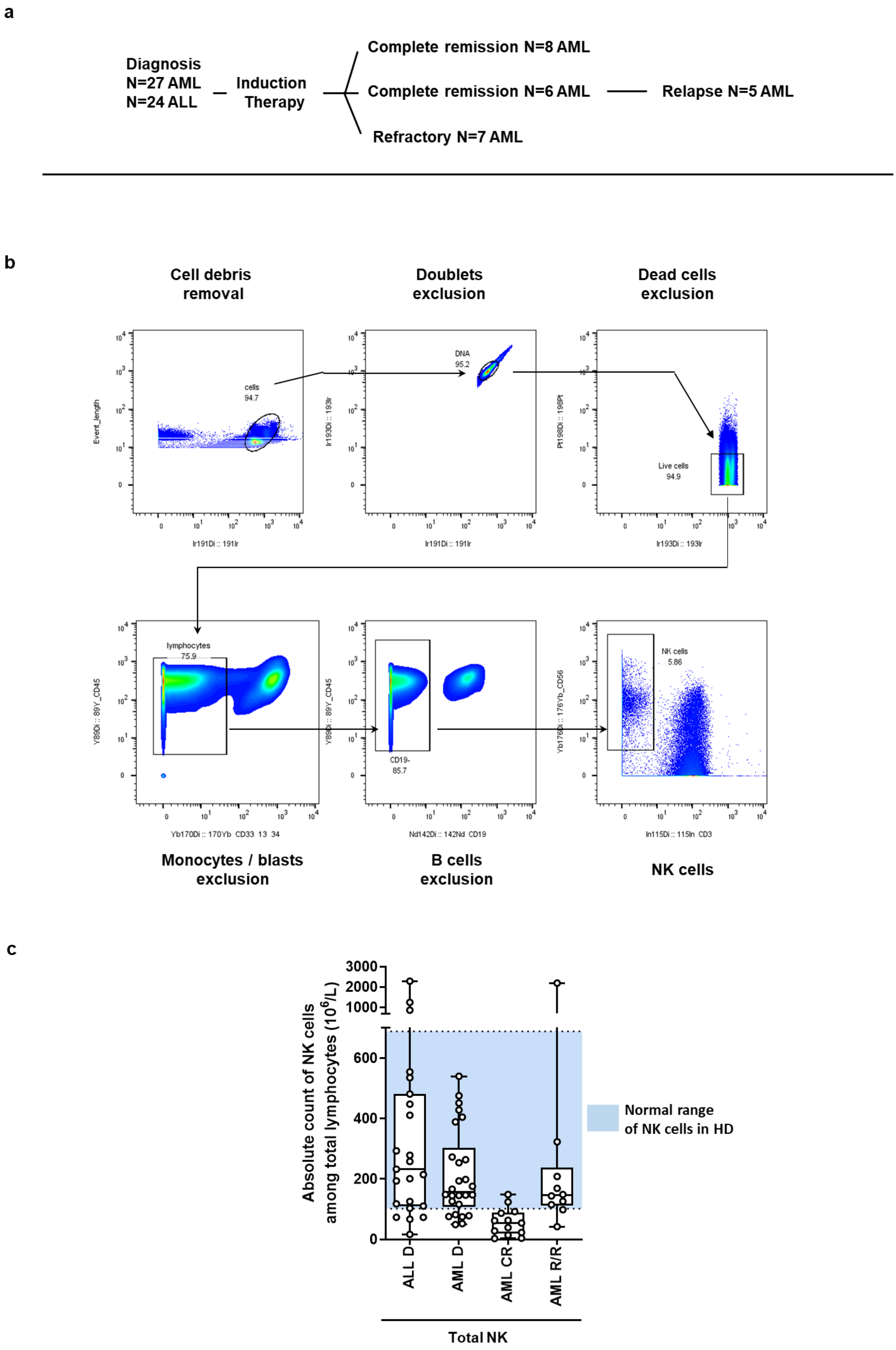
a
Diagnosis
N=27 AML
N=24 ALL
Induction
Therapy
Complete remission N=8 AML
Complete remission N=6 AML
Relapse N=5 AML
Refractory N=7 AML
b
Cell debris
removal
Doublets
exclusion
Dead cells
exclusion
cells
94.7
DNA
95.2
Live cells
94.9
lymphocytes
75.9
CD19-
85.7
NK cells
5.86
Monocytes / blasts
exclusion
B cells
exclusion
NK cells
c
Absolute count of NK cells
among total lymphocytes (10^6/L)
Normal range
of NK cells in HD
ALL D
AML D
AML CR
AML R/R
Total NK

Supplementary figure 6. Absolute counts of NK cells in AML and ALL patients undergoing standard induction chemotherapy. a Total lymphocytes from patients with newly-diagnosed ALL and AML as well as AML patients in complete remission and in relapse/refractory disease were characterized by mass cytometry. **b** Gating strategy used to characterize NK cells among PBMC for healthy donor, AML and ALL patients. PBMCs from HV, AML and ALL patients were stained with metal-conjugated antibodies and analyzed by mass cytometry. After removal of beads, cells were pre-gated as DNA+, cisplatin- cells. Monocytes and leukemic blasts were excluded based on expression of CD13, CD33, and CD34 and on low or absent CD45 expression; B cells and T cells were excluded based on CD19 and CD3 expression, respectively. NK cells were defined as CD3- CD56+ cells. **c** Absolute counts of NK cells among total lymphocyte obtained for AML and ALL patients at different disease stages. Absolute counts of NK cells were calculated using the frequency of NK cells per total lymphocytes obtained by mass cytometry and the absolute counts of total lymphocytes determined at the time of blood collection. The blue field represents the normal range of absolute counts of NK cell obtained in healthy donors (HD) by flow cytometry [1]. In each box plot, the central mark indicates the median, the bottom and top edges of the box indicate the interquartile range (IQR), and the whiskers represent the maximum and minimum data point.

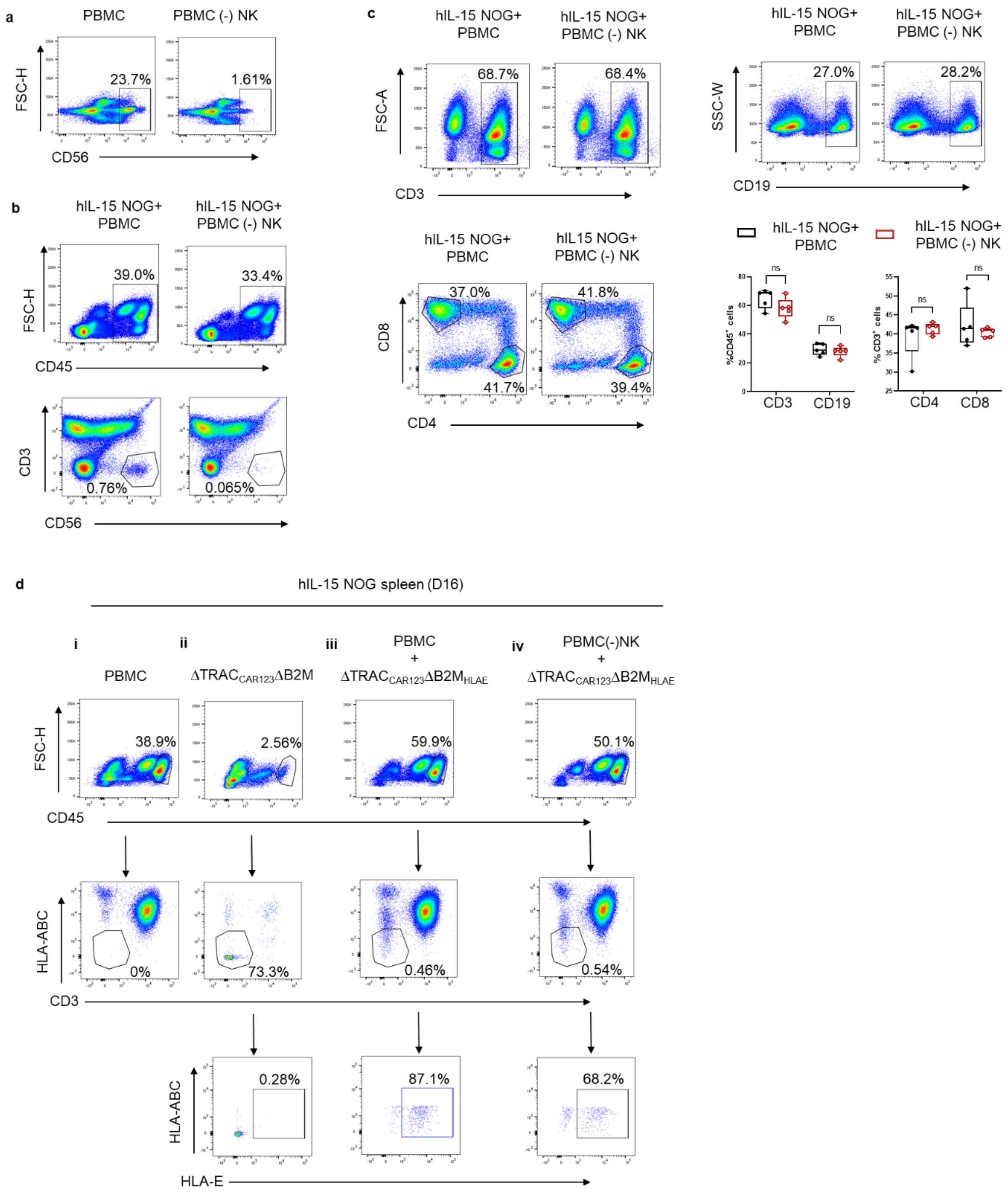

Supplementary figure 7. Targeted insertion of HLA-E at the B2M locus efficiently prevents NK cell-mediated depletion of ΔTRAC$_{CAR}$ΔB2M$_{HLAE}$ cells *in vivo*. a, Flow cytometry plots depicting the CD56(+) fraction of PBMCs injected into hIL-15 NOG mice with (n= 5 mice) or without (n= 5 mice) CD56(+) cell depletion. Live single cells gated on CD45(+) were analyzed for CD56(+) population in control PBMC samples and NK cell-depleted PBMCs prior to adoptive transfer. **b**, Representative flow cytometry plots depicting CD45(+) immune cell and CD3(-)CD56(+) NK cell engraftment in the spleen of hIL-15 NOG mice, 16 days post adoptive transfer. **c**,

Representative flow cytometry analysis of different immune compartments in the spleen of hIL-15 NOG mice, 16 days post injection of either whole PBMCs or NK cell-depleted PBMCs. **c, bottom right,** Box plot documenting the frequency of the different hCD45 (+) cell populations and illustrated in **c** and quantified from hIL-15 NOG mice injected with PBMC with (n= 5 mice) or without (n= 5 mice) CD56(+) cell depletion. In each box plot, the central mark indicates the median, the bottom and top edges of the box indicate the interquartile range (IQR), and the whiskers represent the maximum and minimum data point. For quantitative comparisons, data were analyzed using a two-sided paired t test with a confidence interval of 95%. Non-significant p-values are indicated as "ns". Source data are provided as a Source Data file. **d**, Representative flow cytometry plots depicting gating strategy for identifying injected $\Delta TRAC_{CAR123}\Delta B2M_{HLAE}$ T-cells in the spleen of hIL-15 NOG mice, 4 days post injection. Live single $CD45^{+}$ cells from the spleens of control hIL-15 NOG mice injected with $\Delta TRAC_{CAR123}\Delta B2M$ T-cells alone (i) were gated on CD3(-)HLA-ABC(-) cells-these are the engineered CAR-T cells injected, as evidenced by their absence in the spleen of hIL-15 NOG mice injected with allogeneic PBMCs alone (ii). Illustrated gates were then used to analyze $\Delta TRAC_{CAR123}\Delta B2M$ T-cells for HLA-E expression in samples (iii) and (iv).

Supplemental Tables

Supplementary Table 1. DNA sequences of CAR_m, $HLAE_m$ and rlv CAR-2A-HLAE used to package AAV6 and rlv.

**Construct name**	**DNA sequence and color coding**
**CAR_{mCD123}**	TRAC left homology arm_T2A_Signal peptide_Scfv_CD8 hinge and CD20 mimotopes_Transmembrane domain_41BB_ITAM_Bgh polyA_TRAC Right homology arm<br><br>AAGTAGCCCTGCATTTCAGGTTTCCTTGAGTGGCAGGCCAGGCCTGGCCGTGAACG<br>TTCACTGAAATCATGGCCTCTTGGCCAAGATTGATAGCTTGTGCCTGTCCCTGAGTC<br>CCAGTCCATCACGAGCAGCTGGTTTCTAAGATGCTATTTCCCGTATAAAGCATGAGA<br>CCGTGACTTGCCAGCCCCACAGAGCCCCGCCCTTGTCCATCACTGGCATCTGGACTC<br>CAGCCTGGGTTGGGGCAAAGAGGGAAATGAGATCATGTCCTAACCCTGATCCTCTT<br>GTCCCACAGATATCCAGTCCGGTGAGGGCAGAGGAAGTCTTCTAACATGCGGTGAC<br>GTGGAGGAGAATCCGGGCCCCGGATCCGCTCTGCCCGTCACCGCTCTGCTGCTGCC<br>ACTGGCCCTGCTGCTGCACGCCGCCAGACCCGAAGTCAAACTGGTGGAGTCTGGGG<br>GAGGACTGGTGCAGCCAGGAGGCTCACTGAGCCTGTCCTGCGCCGCTTCCGGCTTC<br>ACCTTCACCGACTACTATATGTCTTGGGTCCGCCAGCCACCTGGGAAGGCTCTGGAG<br>TGGCTGGCACTGATCCGGAGCAAAGCAGATGGATACACCACAGAATATTCTGCCAG<br>TGTGAAGGGCCGCTTCACACTGTCCCGAGACGATTCACAGAGCATTCTGTACCTGCA<br>GATGAACGCTCTGAGACCTGAGGACTCTGCCACTTACTATTGCGCTAGGGATGCAG<br>CCTACTATTCTTACTATAGTCCAGAAGGCGCCATGGACTACTGGGGGCAGGGAACA<br>AGCGTGACTGTCAGCTCCGGAGGAGGAGGATCCGGAGGAGGAGGATCTGGAGGA<br>GGAGGAAGTATGGCTGACTATAAGGATATCGTGATGACCCAGAGCCACAAGTTCAT<br>GTCCACATCTGTGGGCGACCGCGTCAACATTACCTGTAAGGCCTCACAGAATGTGG<br>ATAGCGCCGTCGCTTGGTACCAGCAGAAGCCTGGACAGAGCCCAAAAGCACTGATC<br>TATAGTGCCTCATACCGGTATAGTGGCGTGCCAGACAGATTCACAGGCAGGGGGTC<br>AGGAACTGATTTTACCCTGACAATTTCTAGTGTGCAGGCCGAGGATCTGGCTGTCTA<br>CTATTGCCAGCAGTACTATTCCACCCCCTGGACATTCGGGGGAGGCACAAAGCTGG<br>AAATCAAACGAGGAAGCGGAGGGGGAGGCAGCTGCCCCTACAGCAACCCCAGCCT<br>GTGCAGCGGAGGCGGCGGCAGCTGTCCTTATAGTAATCCAAGCCTGTGTAGCGGC<br>GGAGGGGGTAGCACAACCACACCAGCACCAAGACCACCTACCCCTGCACCAACAAT<br>CGCAAGCCAGCCACTGTCCCTGAGGCCTGAGGCCTGCAGACCAGCAGCAGGAGGA<br>GCAGTGCACACCAGGGGCCTGGATTTTGCCTGCGACATCTATATCTGGGCACCACT<br>GGCCGGAACATGTGGCGTGCTGCTGCTGTCACTGGTCATTACACTGTACTGTAAGC<br>GAGGCCGGAAGAAACTGCTGTATATTTTCAAACAGCCCTTTATGAGACCTGTGCAG<br>ACTACCCAGGAGGAAGACGGCTGCAGCTGTAGGTTCCCCGAGGAAGAGGAAGGCG<br>GGTGTGAGCTGAGGGTCAAGTTTAGCCGCTCCGCAGATGCCCCTGCTTACCAGCAG<br>GGGCAGAATCAGCTGTATAACGAGCTGAATCTGGGACGGAGAGAGGAATACGACG<br>TGCTGGATAAAAGGCGCGGGAGAGACCCCGAAATGGGAGGCAAGCCACGACGGA<br>AAAACCCCCAGGAGGGCCTGTACAATGAACTGCAGAAGGACAAAATGGCAGAGGC<br>CTATAGTGAAATCGGGATGAAGGGAGAGAGAAGGCGCGGCAAAGGGCACGATGG<br>CCTGTACCAGGGGCTGTCTACTGCCACCAAGGACACCTATGATGCTCTGCATATGCA<br>GGCACTGCCTCCAAGGTGATCTAGACTCGAGGTTTAAACCCGCTGATCAGCCTCGAC<br>TGTGCCTTCTAGTTGCCAGCCATCTGTTGTTTGCCCCTCCCCCGTGCCTTCCTTGACCC<br>TGGAAGGTGCCACTCCCACTGTCCTTTCCTAATAAAATGAGGAAATTGCATCGCATT<br>GTCTGAGTAGGTGTCATTCTATTCTGGGGGGTGGGGTGGGGCAGGACAGCAAGGG<br>GGAGGATTGGGAAGACAATAGCAGGCATGCTGGGGATGCGGTGGGCTCTATGACT

	AGTGGCGAATTCCCGTGTACCAGCTGAGAGACTCTAAATCCAGTGACAAGTCTGTCT<br>GCCTATTCACCGATTTTGATTCTCAAACAAATGTGTCACAAAGTAAGGATTCTGATGT<br>GTATATCACAGACAAAACTGTGCTAGACATGAGGTCTATGGACTTCAAGAGCAACA<br>GTGCTGTGGCCTGGAGCAACAAATCTGACTTTGCATGTGCAAACGCCTTCAACAACA<br>GCATTATTCCAGAAGACACCTTCTTCCCCAGCCCAGGTAAGGGCAGCTTTGGTGCCT<br>TCGCAGGCTGTTTCCTTGCTTCAGGAA
**HLAE**$_m$	B2M left homology arm_B2M signal sequence_HLA-G peptide_GS Linker_B2M (codon optimized)_GS linker_HLA-E_Bgh polyA B2M Right homology arms<br><br>CGCGCACCCCAGATCGGAGGGCGCCGATGTACAGACAGCAAACTCACCCAGTCTAG<br>TGCATGCCTTCTTAAACATCACGAGACTCTAAGAAAAGGAAACTGAAAACGGGAAA<br>GTCCCTCTCTCTAACCTGGCACTGCGTCGCTGGCTTGGAGACAGGTGACGGTCCCTG<br>CGGGCCTTGTCCTGATTGGCTGGGCACGCGTTTAATATAAGTGGAGGCGTCGCGCT<br>GGCGGGCATTCCTGAAGCTGACAGCATTCGGGCCGAGATGTCTCGCTCCGTGGCCT<br>TAGCTGTGCTCGCGCTACTCTCTCTTAGCGGCCTCGAAGCTGTTATGGCTCCGCGGA<br>CTTTAATTTTAGGTGGTGGCGGATCCGGTGGTGGCGGTTCTGGTGGTGGCGGCTCC<br>ATCCAGCGTACGCCCAAAATTCAAGTCTACAGCCGACATCCTGCAGAGAACGGCAA<br>ATCTAATTTCCTGAACTGCTATGTATCAGGCTTTCACCCTAGCGATATAGAAGTGGA<br>CCTGCTGAAAAACGGAGAGAGGATAGAAAAGGTCGAACACAGCGACCTCTCCTTTT<br>CCAAGGACTGGAGCTTTTATCTTCTGTATTATACTGAATTTACACCCACGGAAAAAG<br>ATGAGTATGCGTGCCGAGTAAACCACGTCACGCTGTCACAGCCCAAAATAGTAAAA<br>TGGGATCGCGACATGGGTGGTGGCGGTTCTGGTGGTGGCGGTAGTGGCGGCGGA<br>GGAAGCGGTGGTGGCGGTTCCGGATCTCACTCCTTGAAGTATTTCCACACTTCCGTG<br>TCCCGGCCCGGCCGCGGGGAGCCCCGCTTCATCTCTGTGGGCTACGTGGACGACAC<br>CCAGTTCGTGCGCTTCGACAACGACGCCGCGAGTCCGAGGATGGTGCCGCGGGCG<br>CCGTGGATGGAGCAGGAGGGGTCAGAGTATTGGGACCGGGAGACACGGAGCGCC<br>AGGGACACCGCACAGATTTTCCGAGTGAACCTGCGGACGCTGCGCGGCTACTACAA<br>TCAGAGCGAGGCCGGGTCTCACACCCTGCAGTGGATGCATGGCTGCGAGCTGGGG<br>CCCGACAGGCGCTTCCTCCGCGGGTATGAACAGTTCGCCTACGACGGCAAGGATTA<br>TCTCACCCTGAATGAGGACCTGCGCTCCTGGACCGCGGTGGACACGGCGGCTCAGA<br>TCTCCGAGCAAAAGTCAAATGATGCCTCTGAGGCGGAGCACCAGAGAGCCTACCTG<br>GAAGACACATGCGTGGAGTGGCTCCACAAATACCTGGAGAAGGGGAAGGAGACGC<br>TGCTTCACCTGGAGCCCCCAAAGACACACGTGACTCACCACCCCATCTCTGACCATG<br>AGGCCACCCTGAGGTGCTGGGCTCTGGGCTTCTACCCTGCGGAGATCACACTGACC<br>TGGCAGCAGGATGGGGAGGGCCATACCCAGGACACGGAGCTCGTGGAGACCAGG<br>CCTGCAGGGGATGGAACCTTCCAGAAGTGGGCAGCTGTGGTGGTGCCTTCTGGAG<br>AGGAGCAGAGATACACGTGCCATGTGCAGCATGAGGGGCTACCCGAGCCCGTCAC<br>CCTGAGATGGAAGCCGGCTTCCCAGCCCACCATCCCCATCGTGGGCATCATTGCTGG<br>CCTGGTTCTCCTTGGATCTGTGGTCTCTGGAGCTGTGGTTGCTGCTGTGATATGGAG<br>GAAGAAGAGCTCAGGTGGAAAAGGAGGGAGCTACTATAAGGCTGAGTGGAGCGA<br>CAGTGCCCAGGGGTCTGAGTCTCACAGCTTGTAACTGTGCCTTCTAGTTGCCAGCCA<br>TCTGTTGTTTGCCCCTCCCCCGTGCCTTCCTTGACCCTGGAAGGTGCCACTCCCACTG<br>TCCTTTCCTAATAAAATGAGGAAATTGCATCGCATTGTCTGAGTAGGTGTCATTCTAT<br>TCTGGGGGGTGGGGTGGGGCAGGACAGCAAGGGGGAGGATTGGGAAGACAATA<br>GCAGGCATGCTGGGGATGCGGTGGGCTCTATGTCTCTTTCTGGCCTGGAGGCTATC<br>CAGCGTGAGTCTCTCCTACCCTCCCGCTCTGGTCCTTCCTCTCCCGCTCTGCACCCTCT<br>GTGGCCCTCGCTGTGCTCTCTCGCTCCGTGACTTCCCTTCTCCAAGTTCTCCTTGGTG<br>GCCCGCCGTGGGGCTAGTCCAGGGCTGGATCTCGGGGAAGCGGCGGGGTGGCCTG<br>GGAGTGGGGAAGGGGGTGCGCACCCGGGACGCGCGCTACTTGCCCCTTTCGGCGG<br>GGAGCAGGGGAGACCTTTGGCCTACGGCGACGGGAGGGTCGGGACAAAG

**CAR-2A-HLA-E**	EF1alpha promoter_Signal peptide_ScFv_CD8 hinge and mimotopes_Transmembrane domain_41BB_P2A_B2M signal sequence_HLAG peptide_GS linker_B2M (codon optimized)_GS linker_HLA-E<br><br>CGTGAGGCTCCGGTGCCCGTCAGTGGGCAGAGCGCACATCGCCCACAGTCCCCGAG<br>AAGTTGGGGGGAGGGGTCGGCAATTGAACCGGTGCCTAGAGAAGGTGGCGCGGG<br>GTAAACTGGGAAAGTGATGTCGTGTACTGGCTCCGCCTTTTTCCCGAGGGTGGGGG<br>AGAACCGTATATAAGTGCAGTAGTCGCCGTGAACGTTCTTTTTCGCAACGGGTTTGC<br>CGCCAGAACACAGGTAAGTGCCGTGTGTGGTTCCCGCGGGCCTGGCCTCTTTACGG<br>GTTATGGCCCTTGCGTGCCTTGAATTACTTCCACGCCCCTGGCTGCAGTACGTGATTC<br>TTGATCCCGAGCTTCGGGTTGGAAGTGGGTGGGAGAGTTCGAGGCCTTGCGCTTAA<br>GGAGCCCCTTCGCCTCGTGCTTGAGTTGAGGCCTGGCCTGGGCGCTGGGGCCGCCG<br>CGTGCGAATCTGGTGGCACCTTCGCGCCTGTCTCGCTGCTTTCGATAAGTCTCTAGC<br>CATTTAAAATTTTTGATGACCTGCTGCGACGCTTTTTTTCTGGCAAGATAGTCTTGTA<br>AATGCGGGCCAAGATCTGCACACTGGTATTTCGGTTTTTGGGGCCGCGGGCGGCGA<br>CGGGGCCCGTGCGTCCCAGCGCACATGTTCGGCGAGGCGGGGCCTGCGAGCGCGG<br>CCACCGAGAATCGGACGGGGGTAGTCTCAAGCTGGCCGGCCTGCTCTGGTGCCTGG<br>CCTCGCGCCGCCGTGTATCGCCCCGCCCTGGGCGGCAAGGCTGGCCCGGTCGGCAC<br>CAGTTGCGTGAGCGGAAAGATGGCCGCTTCCCGGCCCTGCTGCAGGGAGCTCAAAA<br>TGGAGGACGCGGCGCTCGGGAGAGCGGGCGGGTGAGTCACCCACACAAAGGAAA<br>AGGGCCTTTCCGTCCTCAGCCGTCGCTTCATGTGACTCCACGGAGTACCGGGCGCCG<br>TCCAGGCACCTCGATTAGTTCTCGAGCTTTTGGAGTACGTCGTCTTTAGGTTGGGGG<br>GAGGGGTTTTATGCGATGGAGTTTCCCCACACTGAGTGGGTGGAGACTGAAGTTAG<br>GCCAGCTTGGCACTTGATGTAATTCTCCTTGGAATTTGCCCTTTTTGAGTTTGGATCT<br>TGGTTCATTCTCAAGCCTCAGACAGTGGTTCAAAGTTTTTTTCTTCCATTTCAGGTGT<br>CGTGAGTCGACGCCACCATGGCTCTGCCCGTCACCGCTCTGCTGCTGCCACTGGCCC<br>TGCTGCTGCACGCCGCCAGACCCGAAGTCAAACTGGTGGAGTCTGGGGGAGGACT<br>GGTGCAGCCAGGAGGCTCACTGAGCCTGTCCTGCGCCGCTTCCGGCTTCACCTTCAC<br>CGACTACTATATGTCTTGGGTCCGCCAGCCACCTGGGAAGGCTCTGGAGTGGCTGG<br>CACTGATCCGGAGCAAAGCAGATGGATACACCACAGAATATTCTGCCAGTGTGAAG<br>GGCCGCTTCACACTGTCCCGAGACGATTCACAGAGCATTCTGTACCTGCAGATGAAC<br>GCTCTGAGACCTGAGGACTCTGCCACTTACTATTGCGCTAGGGATGCAGCCTACTAT<br>TCTTACTATAGTCCAGAAGGCGCCATGGACTACTGGGGGCAGGGAACAAGCGTGAC<br>TGTCAGCTCCGGAGGAGGAGGATCCGGAGGAGGAGGATCTGGAGGAGGAGGAAG<br>TATGGCTGACTATAAGGATATCGTGATGACCCAGAGCCACAAGTTCATGTCCACATC<br>TGTGGGCGACCGCGTCAACATTACCTGTAAGGCCTCACAGAATGTGGATAGCGCCG<br>TCGCTTGGTACCAGCAGAAGCCTGGACAGAGCCCAAAAGCACTGATCTATAGTGCC<br>TCATACCGGTATAGTGGCGTGCCAGACAGATTCACAGGCAGGGGGTCAGGAACTG<br>ATTTTACCCTGACAATTTCTAGTGTGCAGGCCGAGGATCTGGCTGTCTACTATTGCC<br>AGCAGTACTATTCCACCCCCTGGACATTCGGGGGAGGCACAAAGCTGGAAATCAAA<br>CGAGGAAGCGGAGGGGGAGGCAGCTGCCCCTACAGCAACCCCAGCCTGTGCAGCG<br>GAGGCGGCGGCAGCTGTCCTTATAGTAATCCAAGCCTGTGTAGCGGCGGAGGGGG<br>TAGCACAACCACACCAGCACCAAGACCACCTACCCCTGCACCAACAATCGCAAGCCA<br>GCCACTGTCCCTGAGGCCTGAGGCCTGCAGACCAGCAGCAGGAGGAGCAGTGCAC<br>ACCAGGGGCCTGGATTTTGCCTGCGACATCTATATCTGGGCACCACTGGCCGGAAC<br>ATGTGGCGTGCTGCTGCTGTCACTGGTCATTACACTGTACTGTAAGCGAGGCCGGA<br>AGAAACTGCTGTATATTTTCAAACAGCCCTTTATGAGACCTGTGCAGACTACCCAGG<br>AGGAAGACGGCTGCAGCTGTAGGTTCCCCGAGGAAGAGGAAGGCGGGTGTGAGC<br>TGAGGGTCAAGTTTAGCCGCTCCGCAGATGCCCCTGCTTACCAGCAGGGGCAGAAT<br>CAGCTGTATAACGAGCTGAATCTGGGACGGAGAGAGGAATACGACGTGCTGGATA

	AAAGGCGCGGGAGAGACCCCGAAATGGGAGGCAAGCCACGACGGAAAAACCCCC<br>AGGAGGGCCTGTACAATGAACTGCAGAAGGACAAAATGGCAGAGGCCTATAGTGA<br>AATCGGGATGAAGGGAGAGAGAAGGCGCGGCAAAGGGCACGATGGCCTGTACCA<br>GGGGCTGTCTACTGCCACCAAGGACACCTATGATGCTCTGCATATGCAGGCACTGCC<br>TCCAAGGGGAAGCGGAGCTACTAACTTCAGCCTGCTGAAGCAGGCTGGAGACGTG<br>GAGGAGAACCCTGGACCTATGTCTCGCTCCGTGGCCTTAGCTGTGCTCGCGCTACTC<br>TCTCTTAGCGGCCTCGAAGCTGTTATGGCTCCGCGGACTTTAATTTTAGGTGGTGGC<br>GGATCCGGTGGTGGCGGTTCTGGTGGTGGCGGCTCCATCCAGCGTACGCCCAAAAT<br>TCAAGTCTACAGCCGACATCCTGCAGAGAACGGCAAATCTAATTTCCTGAACTGCTA<br>TGTATCAGGCTTTCACCCTAGCGATATAGAAGTGGACCTGCTGAAAAACGGAGAGA<br>GGATAGAAAAGGTCGAACACAGCGACCTCTCCTTTTCCAAGGACTGGAGCTTTTATC<br>TTCTGTATTATACTGAATTTACACCCACGGAAAAAGATGAGTATGCGTGCCGAGTAA<br>ACCACGTCACGCTGTCACAGCCCAAAATAGTAAAATGGGATCGCGACATGGGTGGT<br>GGCGGTTCTGGTGGTGGCGGTAGTGGCGGCGGAGGAAGCGGTGGTGGCGGTTCC<br>GGATCTCACTCCTTGAAGTATTTCCACACTTCCGTGTCCCGGCCCGGCCGCGGGGAG<br>CCCCGCTTCATCTCTGTGGGCTACGTGGACGACACCCAGTTCGTGCGCTTCGACAAC<br>GACGCCGCGAGTCCGAGGATGGTGCCGCGGGCGCCGTGGATGGAGCAGGAGGGG<br>TCAGAGTATTGGGACCGGGAGACACGGAGCGCCAGGGACACCGCACAGATTTTCC<br>GAGTGAACCTGCGGACGCTGCGCGGCTACTACAATCAGAGCGAGGCCGGGTCTCA<br>CACCCTGCAGTGGATGCATGGCTGCGAGCTGGGGCCAGACAGGCGCTTCCTCCGCG<br>GGTATGAACAGTTCGCCTACGACGGCAAGGATTATCTCACCCTGAATGAGGACCTG<br>CGCTCCTGGACCGCGGTGGACACGGCGGCTCAGATCTCCGAGCAAAAGTCAAATGA<br>TGCCTCTGAGGCGGAGCACCAGAGAGCCTACCTGGAAGACACATGCGTGGAGTGG<br>CTCCACAAATACCTGGAGAAGGGGAAGGAGACGCTGCTTCACCTGGAGCCCCCAAA<br>GACACACGTGACTCACCACCCCATCTCTGACCATGAGGCCACCCTGAGGTGCTGGG<br>CTCTGGGCTTCTACCCTGCGGAGATCACACTGACCTGGCAGCAGGATGGGGAGGGC<br>CATACCCAGGACACGGAGCTCGTGGAGACCAGGCCTGCAGGGGATGGAACCTTCC<br>AGAAGTGGGCAGCTGTGGTGGTGCCTTCTGGAGAGGAGCAGAGATACACGTGCCA<br>TGTGCAGCATGAGGGGCTACCCGAGCCCGTCACCCTGAGATGGAAGCCGGCTTCCC<br>AGCCCACCATCCCCATCGTGGGCATCATTGCTGGCCTGGTTCTCCTTGGATCTGTGG<br>TCTCTGGAGCTGTGGTTGCTGCTGTGATATGGAGGAAGAAGAGCTCAGGTGGAAA<br>AGGAGGGAGCTACTATAAGGCTGAGTGGAGCGACAGTGCCCAGGGGTCTGAGTCT<br>CACAGCTTGTGA

Supplementary Table 2. List of candidate off-site targets identified by OCA analysis. The position (Genome GRCh38/hg38 version) and sequences of candidate off-sites targeted identified by OCA as well as their position within genes are documented.

Alias	Location	Position GRCh38/hg38 version	Putative hit sequence	Gene (cleavage position)
OS1	chr16	17344058-17344112	TTGCTCCCCAGAGATATTCGGCAATGTCCAGAGACATTTGTGAGACAA	**XYLT1 (Intron)**
OS2	chr14	74807773-74807816	TTGTCAACCAGATAACTGAAAGGAACAGAAGTGTGGAGGCTATCTA	**YLPM1 (Intron)**
OS3	chr1	52056997-52057032	ATAGTTGTCCCCTCGGGAGTGGTAACAATAGTTACCAGTAACTGTGTATCTTCTGTGA	**BTF3L4 (Intron)**
OS4	chr19	34927737-34927771	ATATTGCACAGAAATAGGACCCAGCTTTTTCTTAAGGTTCTTCA	**ZNF30 (Intron)**
OS5	chr10	123100518-123100546	TTCTCCCACAGAACTGGACCACATACTTCTGGGTATTAGTTGAGA	**None**
OS6	chr9	136864517-136864560	TAGTAACACAGAGATGACACATCTCATGGAAGCCTGCAGGCTCTCCA	**EDF1 (Intron)**
OS7	chr17	28610946-28610967	TCTCAGAAGGGACCTCTCTGTTCTAGATGGGGAGGAGATTTGGGGGACCA	**SGK494 (Intron)**
OS8	chr12	57535277-57535310	TAGTACCAAAGAACTGAGGACAGCTAGAGGGCTGGAGGCTCTCCA	**DCTN2 (Intron)**
OS9	chr20	58542177-58542452	ATGTGCGACTGACATTTGGGGCTGAGTCACTGGGGTGGTGGGGACAGATCTTTGGGGCTG	**APCDD1L-AS1 (Intron)**
OS10	chr5	168430996-168431008	TGAATAGCCTCCTCCATAGCTCACTGGTACTCATGCAGGGGCATCTGTGGGATAA	**WWC1 (Intron)**
OS11	chr9	34921696-34921731	GTGTTCCACAGATATACATGGTTTAACATGTGGTATCTGTGGGATAT	**YWHAZP6**
OS12	chr15	64422781-64422808	TAATCCCACAGAAATAAAGTAGGTGGTTTTGCCACATTTGTGGGACCA	**TRIP4 (Intron)**
OS13	chr12	121654150-121654180	TTGACACAAGGGTCAGTGTGGGACAAGAGTGTGGGGTGTGGCCTGTCTCTGTGGGACCA	**MORN3 (Intron)**
OS14	chr1	110162385-110162395	TCTCAACAGATATATGCTAGAGCTGAAGGGGGTCTTGGAGGCCATCCA	**SLC6A17 (Intron)**
OS15	chr8	66414344-66414367	TTATCCTATAAATGTTTTCTAAACTATATGTGTGTGTACCTTCTTATA	**LOC102724687 (Intron)**
OS16	chr7	99697290-99697295	TCTCTGTAGGAAAACCCATTAAGGCTGCTGCTCCCTATGGCAGGCCTGCTTGCAACTCA	**CYP3A51P**
OS17	chr1	155549665-155549686	TACAAGATGATACACATAGGTTATATCCAAGTAGTATACCATTTTATA	**ASH1L (Intron)**
OS18	chr10	102067803-102067835	TGACTACCCTTCAGGCATCAGAACACTCAGGGCCTGGAGGCTTGCTT	**HPS6 (exon non coding sequence)**
OS19	chr7	139889800-139889821	TTGTCCCCCAAAGACCCCACCTTCTAATACTGTCATCTGGGGGTTTA	**TBXAS1 (Intron)**
OS20	chr8	73665696-73665726	TCACTGCTGGTACAAATACAGTCATCCCTAGGTTATCTGTGGGGAAT	**STAU2 (Intron)**
OS21	chr1	1398382-1398442	ATGTTTGTCCCCAGCCACGGCGTCCTGACGGCCGCCAAAGGCTTGAGGGCTATTCA	**CCNL2 (CDS_exon)**
OS22	chr5	103834143-103834147	TGGATATCTCCCAGTAGGGTGTGGAAGGGTTTTTAGAGTAGGGCAA	**LOC105379107 (intron)**

Supplementary Table 3. Sequences of DNA matrices to quantity B2M/TRAC TALEN dependent translocations. A XhoI restriction site (underlined) was added at break points between the B2M and the TRAC sites (bold green and bold blue, respectively) to control for potential contaminations of experimental samples from control matrices

**Name of the translocation**	**DNA Sequence of matrices used to quantity B2M/TRAC TALEN dependent transloctions. XhoI restriction site (underligned) was added at break points between B2M and TRAC sites (bold green and bold blue, respectively) to control for potential contaminations of experimental samples from control matrices**
T1	TATTAAATAAAAGAATAAGCAGTATTATTAAGTAGCCCTGCATTTCAGGTTTCCTTGAGTGGCAGGCCAGGCCTGGCGTGAACGTTCACTGAAATCATGGCCTCTTGGCCAAGATTGATAGCTTGTGCCTGTCCCTGAGTCCCAGTCCATCACGAGCAGCTGGTTTCTAAGATGCTATTTCCCGTATAAAGCATGAGACCGTGACTTGCCAGCCCCACAGAGCCCCGCCCTTGTCCATCACTGGCATCTGGACTCCAGCCTGGGTTGGGGCAAAGAGGGAAATGAGATCATGTCCTAACC**CTGATCCTCTTGTCCCACAGATATAGAACCC**CTCGAG**GAGAGTAGCGCGAGCACAGCTAA**GGCCACGGAGCGAGACATCTCGGCCCGAATGCTGTCAGCTTCAGGAATGCCCGCCAGCGCGACGCCTCCACTTATATTAAACGCGTGCCCAGCCAATCAGGACAAGGCCCGCAGGGACCGTCACCTGTCTCCAAGCCAGCGACGCAGTGCCAGGTTAGAGAGAGGGACTTTCCCGTTTTCAGTTTCCTTTTCTTAGAGTCTCGTGATGTTTAAGAAGGCATGCACTAGACTGGGTGAGTTTGCTGTCTGTACATCGGCGCCCTCCGATCTGGGGTGCGCGCCCCAGCTTGGGACACCGGGCGCTCA
T2	TATTAAATAAAAGAATAAGCAGTATTATTAAGTAGCCCTGCATTTCAGGTTTCCTTGAGTGGCAGGCCAGGCCTGGCGTGAACGTTCACTGAAATCATGGCCTCTTGGCCAAGATTGATAGCTTGTGCCTGTCCCTGAGTCCCAGTCCATCACGAGCAGCTGGTTTCTAAGATGCTATTTCCCGTATAAAGCATGAGACCGTGACTTGCCAGCCCCACAGAGCCCCGCCCTTGTCCATCACTGGCATCTGGACTCCAGCCTGGGTTGGGGCAAAGAGGGAAATGAGATCATGTCCTAACC**CTGATCCTCTTGTCCCACAGATATAGAACCC**CTCGAG**CTTTCTGGCCTGGAGGCTATCCA**GCGTGAGTCTCTCCTACCCTCCCGCTCTGGTCCTTCCTCTCCCGCTCTGCACCCTCTGTGGCCCTCGCTGTGCTCTCTCGCTCCGTGACTTCCCTTCTCCAAGTTCTCCTTGGTGGCCCGCCGTGGGGCTAGTCCAGGGCTGGATCTCGGGGAAGCGGCGGGGTGGCCTGGGAGTGGGGAAGGGGGTGCGCACCCGGGACGCGCGCTACTTGCCCCTTTCGGCGGGGAGCAGGGGAGACCTTTGGCCTACGGCGACGGGAGGGTCGGGACAAAGTTTAGGGCGTCGATAAGCGTCAGAGCGCC
T3	CTCTGGGCAGAACCTGGCCATTCCTGAAGCAAGGAAACAGCCTGCGAAGGCACCAAAGCTGCCCTTACCTGGGCTGGGGAAGAAGGTGTCTTCTGGAATAATGCTGTTGTTGAAGGCGTTTGCACATGCAAAGTCAGATTTGTTGCTCCAGGCCACAGCACTGTTGCTCTTGAAGTCCATAGACCTCATGTCTAGCACAGTTTTGTCTGTGATATACACATCAGAATCCTTACTTTGTGACACATTTGTTTGAGAATCAAAATCGGTGAATAGGCAGACAGACTTGTCACTGGATTTAGAG**TCTCTCAGCTGGTACACGGCAGGGTC**CTCGAG**GAGAGTAGCGCGAGCACAGCTAA**GGCCACGGAGCGAGACATCTCGGCCCGAATGCTGTCAGCTTCAGGAATGCCCGCCAGCGCGACGCCTCCACTTATATTAAACGCGTGCCCAGCCAATCAGGACAAGGCCCGCAGGGACCGTCACCTGTCTCCAAGCCAGCGACGCAGTGCCAGGTTAGAGAGAGGGACTTTCCCGTTTTCAGTTTCCTTTTCTTAGAGTCTCGTGATGTTTAAGAAGGCATGCACTAGACTGGGTGAGTTTGCTGTCTGTACATCGGCGCCCTCCGATCTGGGGTGCGCGCCCCAGCTTGGGACACCGGGCGCTCA
T4	CTCTGGGCAGAACCTGGCCATTCCTGAAGCAAGGAAACAGCCTGCGAAGGCACCAAAGCTGCCCTTACCTGGGCTGGGGAAGAAGGTGTCTTCTGGAATAATGCTGTTGTTGAAGGCGTTTGCACATGCAAAGTCAGATTTGTTGCTCCAGGCCACAGCACTGTTGCTCTTGAAGTCCATAGACCTCATGTCTAGCACAGTTTTGTCTGTGATATACACATCAGAATCCTTACTTTGTGACACATTTGTTTGAGAATCAAAATCGGTGAATAGGCAGACAGACTTGTCACTGGATTTAGAG**TCTCTCAGCTGGTACACGGCAGGGTC**CTCGAG**CTTTCTGGCCTGGAGGCTATCCA**GCGTGAGTCTCTCCTACCCTCCCGCTCTGGTCCTTCCTCTCCCGCTCTGCACCCTCTGTGGCCCTCGCTGTGCTCTCTCGCTCCGTGACTTCCCTTCTCCAAGTTCTCCTTGGTGGCCCGCCGTGGGGCTAGTCCAGGGCTGGATCTCGGGGAAGCGGCGGGGTGGCCTGGGAGTGGGGAAGGGGGTGCGCACCCGGGACGCGCGCTACTTGCCCCTTTCGGCGGGGAGCAGGGGAGACCTTTGGCCTACGGCGACGGGAGGGTCGGGACAAAGTTTAGGGCGTCGATAAGCGTCAGAGCGCC

Supplementary Table 4. List of Translocation specific-oligonucleotides used to quantify potential translocations (T1, T2, T3 and T4) generated by the co-treatment of T-cell with TRAC and B2M TALEN.

Primer name	Sequence	target
In-T1R-T3F	GAGACCGTGACTTGCCAGCC	T1
In_B2Mp	TATAAGTGGAGGCGTCGCGC	
In-T1R-T3F	GAGACCGTGACTTGCCAGCC	T2
Ex_B2Mq	GAGATCCAGCCCTGGACTAG	
Ex-T2R-T4F	AGACCTCATGTCTAGCACAG	T3
Ex_B2Mp	TTGTCCTGATTGGCTGGGCA	
Ex-T2R-T4F	AGACCTCATGTCTAGCACAG	T4
Ex_B2Mq	GAGATCCAGCCCTGGACTAG	

Supplementary Table 5. Experimental layout used to investigate the specificity of CAR_m and $HLAE_m$ targeted insertion in T-cells by TLA analysis.

	Experiental layout				TLA analysis		
Sample name	TALEN Modifications		Insertion		Locus specific primer set		Construct specific primer set
**Sample 1**	TRAC KO	-	CARm123	-	Set 1, 2	-	Set 3, 4
**Sample 2**	TRAC KO	-	-	HLAEm	Set 1, 2	-	-
**Sample 3**	-	B2M KO	-	HLAEm	-	Set 7, 8	-
**Sample 4**	-	B2M KO	CARm123	-	-	Set 7, 8	Set 3, 4
**Sample 5**	TRAC KO	B2M KO	CARm123	-	Set 1, 2	Set 7, 8	Set 3, 4
**Sample 6**	TRAC KO	B2M KO	-	HLAEm	Set 1, 2	Set 7, 8	-
**Sample 7**	TRAC KO	B2M KO	CARm123	HLAEm	Set 1, 2	Set 7, 8	Set 3, 4
**Sample 8**	TRAC KO	B2M KO	rlv CAR-2A-HLA-E	-	Set 1, 2	Set 7, 8	Set 9, 10

Supplementary Table 6. List of primer sets used for TLA analysis of engineered CAR T-cells.

Primer set	Name/View point	Direction	Binding position	Sequence
1	TRAC Locus 1	Reverse	chr14:23,014,817	GTGTCAGACTGGAGAAGATC
		Forward	chr14:23,015,055	CTTCCCAGCAAAGGAACTAT
2	TRAC Locus 2	Reverse	chr14:23,018,088	CTCTTTGCTTTCTCATCCCT
		Forward	chr14:23,018,269	GGACTCTAGAATGAAGCCAG
3	CARm123 (Scfv)	Reverse	CARm123	TGACGGGCAGAGCGGATC
		Forward	CARm123	ACCTGAGGACTCTGCCACTT
4	CARm123 (ITAM)	Reverse	CARm123	AGTGTAATGACCAGTGACAG
		Forward	CARm123	GGAAGACAATAGCAGGCAT
5	HLAEm (Locus 1)	Reverse	HLAEm	GAACCGCCACCACCCATG
		Forward	HLAEm	CTGCGCGGCTACTACAAT
6	HLAEm (Locus 2)	Reverse	HLAEm	GCTTCCATCTCAGGGTGA
		Forward	HLAEm	GGAAGACAATAGCAGGCAT
7	B2M Locus 1	Reverse	chr13:45,002,707	TTAGTGAGAGTTTGGACTGC
		Forward	chr13:45,003,442	CCTGAAGTCCTAGAATGAGC
8	B2M Locus 2	Reverse	chr13:45,008,173	ATGTATTTGTGCAAGTGCTG
		Forward	chr13:45,008,336	TGGATTGGTATCTGAGGCTA
9	rlv CAR-2A-HLA-E (EF1a)	Reverse	CAR-2A-HLA-E	ACTAATCGAGGTGCCTGG
		Forward	CAR-2A-HLA-E	CCATTTCAGGTGTCGTGA
10	rlv CAR-2A-HLA-E (HLA-E)	Reverse	CAR-2A-HLA-E	GAACCGCCACCACCCATG
		Forward	CAR-2A-HLA-E	CTGCGCGGCTACTACAAT

Supplementary Table 7. Antibodies used to assess expression of relevant markers on the surface of engineered T-cells

Antibody	Dilution	Fluorophore	Manufacturer	Catalogue #
CD56	1:50	APC	Miltenyi	130-113-310
NKG2A	1:50	PE-Vio700	Miltenyi	130-113-567
NKG2C	1:50	PE	Miltenyi	130-119-776
HLA ABC	1:50	VioBlue	Miltenyi	130-120-435
HLA E	1:50	APC	Miltenyi	130-117-402
TCR α/β	1:25	PE-Vio770	Miltenyi	130-109-922
CD3	1:50	PE	Miltenyi	130-113-139
CD4	1:50	VioBlue	Miltenyi	130-113-258
CD8	1:50	APC	Miltenyi	130-110-679
Viability	1:1000	e780	eBiosciences	65-0865-14
Rituximab	1:10	FITC	R&D systems	FAB9575G

Supplementary Table 8. Antibodies and reagents used to assess the cytolytic activity of NK cells present in healthy donors or AML patients' PBMCs and to assess the extent of engineered $\Delta TRAC_{CAR}\Delta B2M_{HLAE}$ target enrichment with our without PBMC effectors.

Antibodies and Reagents	Dilution	Fluorophore	Manufacturer	Catalogue #
Anti-Human CD56	1:15	BV605	BD Biosciences	562780
Anti-Human CD45	1:12.5	BV786	BD Biosciences	563716
Anti-Human CD3	1:25	PerCP-Vio700	Miltenyi	130-113-141
Anti-Human HLA-E	1:40	Pe-Vio615	Miltenyi	130-117-403
Anti-Human IFN-γ	1:30	AF700	BD Biosciences	557995
LIVE/DEAD Fixable	1:100	Near -IR	Invitrogen	L10119
Celltrace	1:2500	Violet dye	Life Technologies	C34557
Golgistop	1:300	NA	BD Biosciences	51-2092KZ
Countbright absolute counting beads	1:100	APC	Life Technologies	C36950
BD Cytofix/Cytoperm	NA	NA	BD Biosciences	554714

Reference

1. Melzer, S. *et al.* Reference intervals for leukocyte subsets in adults: Results from a population-based study using 10-color flow cytometry. *Cytom. Part B - Clin. Cytom.* (2015) doi:10.1002/cyto.b.21234.
