# Supplementary material for: Endowing universal CAR T-cell with immune-evasive properties using TALEN-gene editing
Source: Nat Commun. 2022 Jun 30;13:3453. doi: 10.1038/s41467-022-30896-2 (PMC9247096; doi:10.1038/s41467-022-30896-2)
Supplement: Supplementary file 2 — Reporting Summary [file 41467_2022_30896_MOESM2_ESM.pdf]

nature research

Corresponding author(s): Julien Valton

Last updated by author(s): 13/04/22

Reporting Summary

Nature Research wishes to improve the reproducibility of the work that we publish. This form provides structure for consistency and transparency in reporting. For further information on Nature Research policies, see our Editorial Policies and the Editorial Policy Checklist.

Statistics

For all statistical analyses, confirm that the following items are present in the figure legend, table legend, main text, or Methods section.

n/a	Confirmed	
☐	☒	The exact sample size (*n*) for each experimental group/condition, given as a discrete number and unit of measurement
☐	☒	A statement on whether measurements were taken from distinct samples or whether the same sample was measured repeatedly
☐	☒	The statistical test(s) used AND whether they are one- or two-sided<br>*Only common tests should be described solely by name; describe more complex techniques in the Methods section.*
☒	☐	A description of all covariates tested
☒	☐	A description of any assumptions or corrections, such as tests of normality and adjustment for multiple comparisons
☐	☒	A full description of the statistical parameters including central tendency (e.g. means) or other basic estimates (e.g. regression coefficient) AND variation (e.g. standard deviation) or associated estimates of uncertainty (e.g. confidence intervals)
☐	☒	For null hypothesis testing, the test statistic (e.g. *F*, *t*, *r*) with confidence intervals, effect sizes, degrees of freedom and *P* value noted<br>*Give P values as exact values whenever suitable.*
☒	☐	For Bayesian analysis, information on the choice of priors and Markov chain Monte Carlo settings
☒	☐	For hierarchical and complex designs, identification of the appropriate level for tests and full reporting of outcomes
☒	☐	Estimates of effect sizes (e.g. Cohen's *d*, Pearson's *r*), indicating how they were calculated

Our web collection on statistics for biologists contains articles on many of the points above.

Software and code

Policy information about availability of computer code

Data collection	IIn vivo tumor cell line bioluminescence signal was aquired using perkin elmer Living Image software v4.5.2. Flow cytometry data were aquired using Macsquantify software v2.11 (Macsquant cytometer), BD FACS DIVA software v9.0 (BD CANTO cytometer), NovoExpress software v1.5.6 (Agilent). For measuring cell viability and concentration, data were aquired using ViCell XR software v2.06.2 and NucleoView™ software v1.2.0.0
Data analysis	Macsquantify software v2.11 (Macsquant cytometer), BD FACS DIVA software v9.0 (BD CANTO cytometer), FLowJo software FlowJo V.10.6.1 or FlowJo V10.6.2 (Treestar), GraphPad Prism software v9.2.0, LivingImage v4.5.2.

For manuscripts utilizing custom algorithms or software that are central to the research but not yet described in published literature, software must be made available to editors and reviewers. We strongly encourage code deposition in a community repository (e.g. GitHub). See the Nature Research guidelines for submitting code & software for further information.

Data

Policy information about availability of data

All manuscripts must include a data availability statement. This statement should provide the following information, where applicable:

- Accession codes, unique identifiers, or web links for publicly available datasets
- A list of figures that have associated raw data
- A description of any restrictions on data availability

The authors declare that the data supporting the findings of this study are available in the article, in the supplementary information files, and/or in the source data file included to the supplementary information files.

Field-specific reporting

Please select the one below that is the best fit for your research. If you are not sure, read the appropriate sections before making your selection.

☒ Life sciences ☐ Behavioural & social sciences ☐ Ecological, evolutionary & environmental sciences

For a reference copy of the document with all sections, see nature.com/documents/nr-reporting-summary-flat.pdf

Life sciences study design

All studies must disclose on these points even when the disclosure is negative.

Sample size	For experiment other than in vivo studies, analyses were performed on at least 3 independently obtained cell batches. No calculation was performed to predetermine sample size. Sample size was determined from analogous studies performed by us and others where the differences between groups were expected to be comparable and the same statistical methods could be applied.<br>For animal studies, sample size was determined by power analysis (Charan, J. & Biswas, T. Indian J Psychol Med 35, 121-126, 2013) and corroborated by numerous comparable published studies using equal number or fewer animals per groups.
Data exclusions	No data were excluded from analysis
Replication	The observations concerning the characterization of engineered cells were replicated by repeating the experiments on cell batches obtained as independently as possible. In general, batches were obtained by procedures performed at different times (at least a week apart) using shared reagents and most of the time by the same person. In some cases, different donors were engineered in parallel in the same procedures using the same batches of reagents. in less than 10% of the time, experiments failed to produce the desired cells to perform further experiment (due to low transduction efficiency or poor viability of growth of the cells). We confirm that once sufficient number of cells were generated from a given donor with the expected engineered characteristics, all replication attempts were successful.
Randomization	For animal studies, mice were not distributed randomly into different experimental groups. Bodyweight was measured for all mice and groups were determined to minimize the differences in average bodyweight between groups.
Blinding	For animal studies, acquisition of the data were blinded as mice were identified by a tag number rather and not directly by which biological group they belonged. Data analysis was not blinded as it was necessary to group the data belonging to the same experimental group to process the bioluminescence data.

Reporting for specific materials, systems and methods

We require information from authors about some types of materials, experimental systems and methods used in many studies. Here, indicate whether each material, system or method listed is relevant to your study. If you are not sure if a list item applies to your research, read the appropriate section before selecting a response.

Materials & experimental systems

n/a	Involved in the study
☐	☒ Antibodies
☐	☒ Eukaryotic cell lines
☒	☐ Palaeontology and archaeology
☐	☒ Animals and other organisms
☐	☒ Human research participants
☒	☐ Clinical data
☒	☐ Dual use research of concern

Methods

n/a	Involved in the study
☒	☐ ChIP-seq
☐	☒ Flow cytometry
☒	☐ MRI-based neuroimaging

Antibodies

Antibodies used

Antibody Dilution Fluorophore Manufacturer Catalogue #
CD56 1:50 APC Miltenyi 130-113-310
NKG2A 1:50 PE-Vio700 Miltenyi 130-113-567
NKG2C 1:50 PE Miltenyi 130-119-776
HLA ABC 1:50 VioBlue Miltenyi 130-120-435
HLA E 1:50 APC Miltenyi 130-117-402
TCR α/β 1:25 PE-Vio770 Miltenyi 130-109-922
CD3 1:50 PE Miltenyi 130-113-139
CD4 1:50 VioBlue Miltenyi 130-113-258
CD8 1:50 APC Miltenyi 130-110-679
Viability 1:1000 e780 eBiosciences 65-0865-14
Rituximab 1:10 FITC R&D systems FAB9575G
Anti-Human CD56 1:15 BV605 BD Biosciences 562780

CD22EC-mFc 1:300 Lakepharma, custom synthesis
Goat Anti-Mouse IgG 1:100 APC Jackson 115135164
TCRab 1:100 PE Miltenyi 130113539
HLA-E 1:100 PECy7 Biolegend 342608
HLA-ABC 1:100 VioBlue Miltenyi 130120435
CD56 1:100 PercPCy5.5 Biolegend 362526
CD19 1:50 FITC BD bioscience 555412
Anti-Human CD45 1:12.5 BV786 BD Biosciences 563716
Anti-Human CD3 1:25 PerCP-Vio700 Miltenyi 130-113-141
Anti-Human HLA-E 1:40 Pe-Vio615 Miltenyi 130-117-403
Anti-Human IFN-γ 1:30 AF700 BD Biosciences 557995
LIVE/DEAD Fixable 1:100 Near -IR Invitrogen L10119
Celltrace 1:2500 Violet dye Life Technologies C34557
Golgistop 1:300 NA BD Biosciences 51-2092KZ
Countbright absolute counting beads 1:100 APC Life Technologies C36950
BD Cytofix/Cytoperm NA NA BD Biosciences 554714

Validation

Commercial antibody validations were performed by suppliers. The detection capacity of the custom-made CD22EC-mFc protein from Lake was assessed by incubating 50K T-cell expressing the anti-CD22 CAR construct with different diluted solutions of CD22EC-mFc (from 1:1000, 1:750, 1:500, 1:300-1:100, 1:50 dilution factor) for 30 min at 4°C in FACS buffer (4% FBS + 5mM EDTA + 0.05% azide in PBS, 20 µL/well of 96 well plate). Cells were then washed with PBS (150 µL/well), spun at 300 x g for 2 minutes and resuspended in a solution of Goat Anti-Mouse IgG (APC) diluted 1:100 in FACS buffer for 15 min at 4°C. Cells were then washed with PBS (150 µL/well), spun at 300 x g for 2 minutes and resuspended in 100uL of fixating buffer (4% paraformaldehyde in PBS) and eventually analyzed by FACS. The 1:300 dilution of custom-made CD22EC-mFc protein gave us the best signal over noise ratio and the maximum mean fluorescence intensity difference with respect to the unlabeled control.

Eukaryotic cell lines

Policy information about cell lines

Cell line source(s)

MOLM13-nanoLuc-GFP and RAJI-Luc-GFP were engineered out of MOLM13 and RAJI cells (DSMZ, cat# ACC 554 and ATCC, cat# CCL-86, respectively) using an in house rLV encoding NanoLuc_T2A_EGFP construct and AMSbio cat# LVP323-PBS, respectively, using the manufacturers' protocols.

Authentication

The cell lines were authenticated by the provider (ATCC and DSMZ) by DNA profiling

Mycoplasma contamination

Cell lines have not been tested for mycoplasma contamination in the last 12 months.

Commonly misidentified lines
(See ICLAC register)

No cell line from the commonly misidentified line register was used in this study

Animals and other organisms

Policy information about studies involving animals; ARRIVE guidelines recommended for reporting animal research

Laboratory animals

6 to 8 week old female NOD.Cg-Prkdcscid Il2rgtm1Wjl/SzJ (NSG) mice and NOD.Cg-Prkdcscid Il2rgtm1Sug Tg(CMV-IL2/IL15)1-1Jic/JicTac (hIL-15 NOG) mice were obtained from The Jackson Laboratory and Taconic Biosciences, respectively and were used in this study to perform experiments documented in Fig. 2 and Fig. 5 of this manuscript

Wild animals

The study did not involve wild animals

Field-collected samples

The study did not involve samples collected from the field.

Ethics oversight

All procedures involving animals were performed in accordance with regulations and established guidelines and were reviewed and approved by the Cellectis Institutional Animal Care and Use Committee (IACUC) as well as by the Animal Ethical Committee at Mispro-Biotech (New York, NY) which hosts our animal studies.

Note that full information on the approval of the study protocol must also be provided in the manuscript.

Human research participants

Policy information about studies involving human research participants

Population characteristics

PBMCs from ALL and AML patients were obtained from the HEMATOBIO cohort (NCT02320656).

Recruitment

The PBMC sample from AML and ALL patients from the HEMATOBIO cohort (NCT02320656) were selected on the basis of the stage of their disease (Diagnostic/Complete response/Relapse-refractory). The source file documents the patient characteristics.

Ethics oversight

Samples of human origin and associated data were obtained from the IPC / CRCM / UMR 1068 Tumour Bank, that operates under the authorization # AC-2007-33 granted by the French Ministry of Research (Ministère de la Recherche et de l'Enseignement Supérieur). Prior to scientific use of samples and data, patients were appropriately informed and asked to

	consent in writing, in compliance with French and European regulations. The project was approved by the IPC Institutional Review Board (Comité d'Orientation Stratégique, COS) as well as the Committee for the Protection of Persons South Mediterranean I (#2013-AO1437-38).

Note that full information on the approval of the study protocol must also be provided in the manuscript.

Flow Cytometry

Plots

Confirm that:

☒ The axis labels state the marker and fluorochrome used (e.g. CD4-FITC).

☒ The axis scales are clearly visible. Include numbers along axes only for bottom left plot of group (a 'group' is an analysis of identical markers).

☒ All plots are contour plots with outliers or pseudocolor plots.

☒ A numerical value for number of cells or percentage (with statistics) is provided.

Methodology

Sample preparation	All flow cytometry samples were prepared from primary cells engineered in vitro from human PBMCs. Cells were harvested and stained in staining buffer (PBS, 0.1mM EDTA, 0.03% Na Azide). In general, staining consisted of 15 minute incubation with staining reagents at 4°C, followed by washes in excess volume of staining buffer. Samples were fixed in 0.1% PFA before aquisition.
Instrument	MacsQuant (Miltenyi) or BD CANTO analyzer (Becton Dickinson), NovoCyte Penteon (Agilent) for Flow cytometry and Helios® (Fluidigm) for Mass cytometry
Software	Flow cytometry data were aquired using Mancsquantify software v2.11 (Macsquant cytometer) or BD FACS DIVA software (BD CANTO cytometer) and analyzed using FLowJo software FlowJo V.10.6.1 or FlowJo V10.6.2 (Treestar).
Cell population abundance	No fluorescence activated cell sorting (FACS) was performed.
Gating strategy	Regarding conventional flow cytometry analysis, gating strategy generally consisted of first FSC/SSC gating, followed by doublet exclusion (using FSC-H vs. FSC-W dot plot), and dead cell exclusion using fixeable viability dye vs. FSC dot plot. The boundary for positive vs negative population was determined differently depending on what parameter was analyzed. For instance, for gating on TCR-negative cells, we used non gene edited T cells which are 95-100% TCR-positive to choose where TCR-negative cells should be. For most markers, we used "fluorescence minus one" controls where the same cells are stained with all but one antibody to determined above which fluorescence values cells should be considered positive.<br>Regarding mass cytometry analysis of NK cells, gating strategy used to characterize NK cells among PBMC for healthy donor, AML and ALL patients was performed as followed. PBMCs from HV, AML and ALL patients were first stained with metal-conjugated antibodies and analyzed by mass cytometry. After removal of beads, cells were pre-gated as DNA+, cisplatin-cells. Monocytes and leukemic blasts were excluded based on expression of CD13, CD33, and CD34 and on low or absent CD45 expression; B cells and T cells were excluded based on CD19 and CD3 expression, respectively. NK cells were defined as CD13-CD33-CD34-CD45+CD3-CD19-CD56+ and exported using FlowJo V10.6.2. Consensus files were generated for NKG2C(+) NK cells and NKG2C(-) NK cells from healthy volunteers and AML or ALL patients with a fixed number of NK cells. Data were arcsinh-transformed with a cofactor of 5. NK cell populations were automatically defined using the optimized parameters for T-distributed stochastic neighbor embedding (opt-SNE) algorithm.

☒ Tick this box to confirm that a figure exemplifying the gating strategy is provided in the Supplementary Information.
